# Microbial community structure and dynamics in thermophilic composting viewed through metagenomics and metatranscriptomics

**DOI:** 10.1038/srep38915

**Published:** 2016-12-12

**Authors:** Luciana Principal Antunes, Layla Farage Martins, Roberta Verciano Pereira, Andrew Maltez Thomas, Deibs Barbosa, Leandro Nascimento Lemos, Gianluca Major Machado Silva, Livia Maria Silva Moura, George Willian Condomitti Epamino, Luciano Antonio Digiampietri, Karen Cristina Lombardi, Patricia Locosque Ramos, Ronaldo Bento Quaggio, Julio Cezar Franco de Oliveira, Renata Castiglioni Pascon, João Batista da Cruz, Aline Maria da Silva, João Carlos Setubal

**Affiliations:** 1 Departamento de Bioquímica, Instituto de Química, Universidade de São Paulo, São Paulo, Brazil; 2 Programa de Pós-Graduação Interunidades em Bioinformática, Universidade de São Paulo, São Paulo, Brazil; 3 Escola de Artes, Ciências e Humanidades, Universidade de São Paulo, São Paulo, Brazil; 4 Fundação Parque Zoológico de São Paulo, São Paulo, Brazil; 5 Departamento de Ciências Biológicas, Universidade Federal de São Paulo, São Paulo, Brazil; 6 Biocomplexity Institute of Virginia Tech, Blacksburg, VA, USA

## Abstract

Composting is a promising source of new organisms and thermostable enzymes that may be helpful in environmental management and industrial processes. Here we present results of metagenomic- and metatranscriptomic-based analyses of a large composting operation in the São Paulo Zoo Park. This composting exhibits a sustained thermophilic profile (50 °C to 75 °C), which seems to preclude fungal activity. The main novelty of our study is the combination of time-series sampling with shotgun DNA, 16S rRNA gene amplicon, and metatranscriptome high-throughput sequencing, enabling an unprecedented detailed view of microbial community structure, dynamics, and function in this ecosystem. The time-series data showed that the turning procedure has a strong impact on the compost microbiota, restoring to a certain extent the population profile seen at the beginning of the process; and that lignocellulosic biomass deconstruction occurs synergistically and sequentially, with hemicellulose being degraded preferentially to cellulose and lignin. Moreover, our sequencing data allowed near-complete genome reconstruction of five bacterial species previously found in biomass-degrading environments and of a novel biodegrading bacterial species, likely a new genus in the order Bacillales. The data and analyses provided are a rich source for additional investigations of thermophilic composting microbiology.

Typical aerobic composting is a self-heating process in which microbial metabolism drives the temperature above 50 °C, followed by sustained high temperatures between 60–80 °C, and then followed by gradual cooling of the compost pile^1^,^2^. The biological decomposition of organic matter is performed by mesophilic and thermophilic microbial consortia with distinct physiological requirements and tolerances, consistent with the continuously changing environment throughout composting^3^,^4^,^5^,^6^,^7^. Bacterial phyla including Proteobacteria, Firmicutes, Bacteroidetes and Actinobacteria are routinely found in composting, being more or less abundant depending on the starting materials and the composting procedure^1^,^4^,^7^,^8^,^9^,^10^,^11^,^12^. Generally, fungi are not detected in composting piles above 65 °C, suggesting that their degradative activities during the thermophilic stages of composting are minor compared to that of bacteria^13^,^14^. Therefore, the current understanding is that bacteria are the dominant degraders in thermophilic composting processes^13^,^15^,^16^, while fungi come into play during the cooling and curing phases^17^.

Composting is widely considered as a promising source of new thermophilic bacteria^5^,^7^,^18^ and novel thermostable enzymes, particularly those related to biomass degradation, which have many advantages for industrial applications^15^,^19^,^20^,^21^,^22^,^23^. Despite intensive studies to investigate thermophilic composting ecosystems, using culture-dependent^16^,^24^ and culture-independent approaches^4^,^10^,^11^,^13^,^15^,^25^, information regarding functional aspects of associated microbiota is still limited. In this regard, metagenomics and metatranscriptomics are valuable approaches to expand the repertoire of known biodegrading microorganisms and their active functional metabolic potential during thermophilic composting.

The goal of this study was to perform a comprehensive investigation of the structure, dynamics, and metabolic functions of the microbiota in a thermophilic composting operation at the São Paulo Zoo Park, which is located within the urban area of the São Paulo city (Brazil) and includes a remnant Atlantic rain forest patch. This composting facility was designed to compost all organic waste produced in the park. In a previous study^13^, we have shown that the São Paulo Zoo composting harbors considerable microbial diversity. Here we extend the previous work in three important ways: first, in addition to shotgun sequencing data, we obtained 16S rRNA amplicon data; second, we also obtained metatranscriptomics (RNA-seq) data; and third, these data were generated based on several time-series samples of the 99-day-long process. To our knowledge, this is the first study combining all three data types generated from time-series samples from a full-scale composting operation.

## Results and Discussion

### Composting time-series sampling and high-throughput sequencing

We used shotgun DNA, 16S rRNA gene amplicon, and metatranscriptome high-throughput sequencing of time-series samples collected at the composting facility of São Paulo Zoo Park, São Paulo, Brazil. Supplementary Fig. S1 online shows the workflow used to generate the results we discuss here. We have collected nine time-series samples from composting cell ZC4 (days 1, 3, 7, 15, 30, 64, 67, 78 and 99) and five from composting cell ZC3 (days 1, 30, 64, 78 and 99). Sequence dataset accession numbers are listed in Supplementary Table S1. The main characteristics of samples collected from the two composting cells are summarized in Table 1 (ZC4) and Supplementary Table S2 (ZC3). Because ZC4 is the more extensive dataset, we base most of our discussion on it, referencing ZC3 when necessary.

ZC4 and ZC3 exhibited a sustained thermophilic profile (Supplementary Fig. S2), with average temperatures ranging from 50 °C to 75 °C. The amplitude of temperature variation between collection points in ZC4 and ZC3 cells, which in some days reached ~20 °C (Table 1 and Supplementary Table S2), suggests a heterogeneity of the semi-static composting process, reflecting differences in compaction and aeration of the pile as well as in the active microorganism populations. When the temperature dropped below 55 °C, turning of the pile was performed to restore aeration and thermophilic conditions (Supplementary Fig. S2). ZC4 and ZC3 presented C/N ratio in the range of 15:1 (Supplementary Tables S3 and S4), which is half of the recommended value (30:1)^1^ estimated when the composting cells were built. Nevertheless, the decrease in C/N ratio was within the recommended values (<0.75), reflecting an acceptable loss of carbon as CO_2_ as well as the recycling of nitrogen, instead of loss as ammonia gas or through leaching^1^,^2^,^26^.

Metagenomic DNA extracted from the time-series samples listed on Table 1 and Supplementary Table S2 online was submitted to shotgun and to 16S rRNA gene amplicon high-throughput sequencing. Eight of nine samples from ZC4 were also used for metatranscriptomic profiling aiming to reveal active metabolic pathways during thermophilic composting. Detailed metrics of sequencing and assembly data for ZC4 and ZC3 samples are presented in Supplementary Tables S5 and S6, respectively.

### Variation of microbial community composition and diversity during composting

All compost samples we have collected are dominated by bacterial species (Supplementary Tables S7 and S8), regardless of composting age. The bacteria domain represented nearly 100% of classified reads, and at least 84% of all reads, in all samples.

Analysis of the bacterial community structure at the levels of phylum and order by shotgun DNA as well as by 16S rRNA gene amplicon sequencing yields two main results: 1) shotgun DNA and 16S amplicon results by and large agree with each other; 2) the phyla and orders that are most abundant agree with those found in previous studies^1^,^4^,^7^,^8^,^9^,^10^,^11^,^12^. The four most abundant phyla throughout the composting process are Firmicutes, Proteobacteria, Bacteroidetes and Actinobacteria (Supplementary Fig. S3). These four phyla account for at least 85% of all classified reads in all samples.

**Figure 1:**
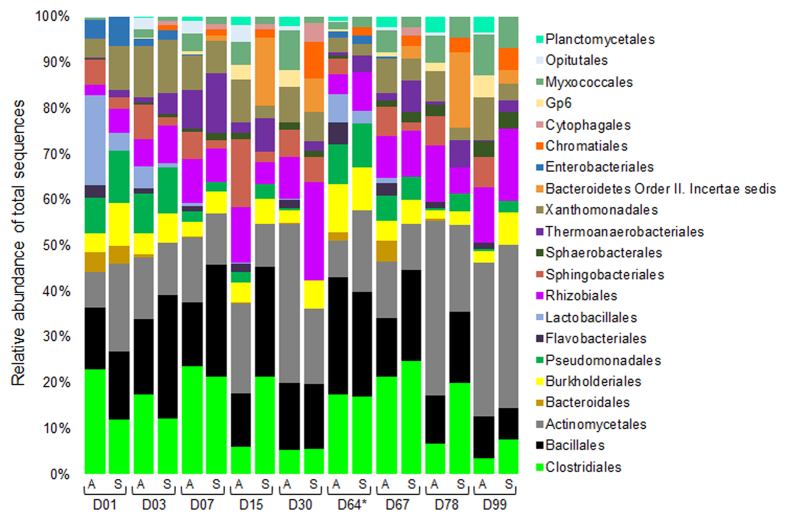
Relative abundance of bacteria in ZC4 samples at the order level. Taxonomic assignments were obtained from 16S rRNA amplicon (A) and shotgun (S) sequencing datasets using RDP classifier and MG-RAST (M5NR) database, respectively. Only orders with relative abundance ≥2% are shown. Unassigned 16S (10–30%) and DNA shotgun reads (10.1–14.6%) were excluded. Samples are referred to by the letter D followed by the collection day. Asterisk indicates one day after the turning procedure.

At the order level there is far more diversity within samples as well as significant variation among them (Fig. 1). Noteworthy is the relative high abundance of orders Clostridiales, Bacillales, and Actinomycetales in most samples, regardless of the sequencing dataset analyzed (16S amplicon or shotgun). Among the most abundant genera within these orders (Supplementary Table S9), we highlight *Clostridium, Symbiobacterium,* and *Thermaerobacter* for Clostridiales, and *Geobacillus, Bacillus*, and *Ureibacillus* for Bacillales. We also feature *Thermopolyspora*, *Thermobispora,* and *Thermomonospora* among the abundant genera within Actinomycetales. These genera comprise bacterial species that can survive the thermophilic stages of composting and are hypothesized to play an important role in biomass degradation during the composting process^4^,^12^,^13^,^16^,^25^,^27^,^28^,^29^,^30^.

Similar profiles for phylum (Supplementary Fig. S4) and order (Supplementary Fig. S5) were observed for ZC3. Indeed, ZC4 and ZC3 appear to share much the same groups of microorganisms according to distance comparisons of corresponding time-series samples (Supplementary Fig. S6), despite some differences in the relative abundance over time for a few groups, as exemplified by Clostridiales (more abundant in D01 in ZC3 but abundant throughout in ZC4; compare Fig. 1 and Supplementary Fig. S5). On the other hand, the distance analyses showed a significant variation for the more abundant groups among the time-series samples of the same cell, indicating the highly dynamic nature of the composting process.

**Figure 2:**
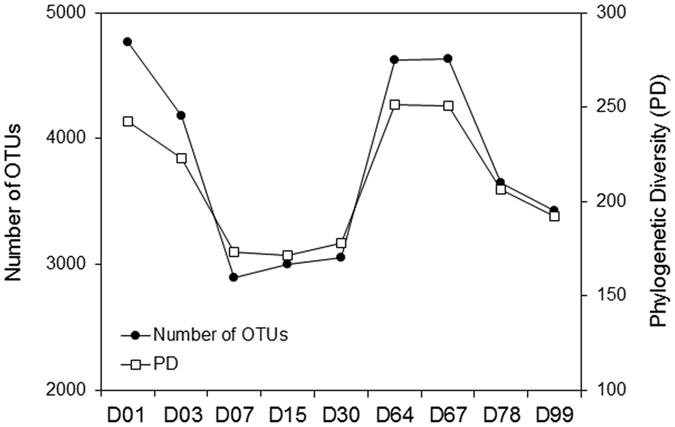
ZC4 phylogenetic diversity variation. Profile of the number of OTUs estimated by 16S rRNA amplicon sequencing and phylogenetic diversity index (alpha rarefaction) during ZC4 composting. Intervals on the horizontal axis do not represent chronological time. Samples are referred to by the letter D followed by the collection day.

The variation in number of Operational Taxonomic Units (OTUs) and phylogenetic diversity index over time based on 16S data (Fig. 2) shows that the highest diversity was found in sample D01 and in samples D64 and D67 (right after the turning procedure). These results suggest the existence of two stages in the composting process we analyzed: the first from the beginning (D01) to D30, and the second from D64 to the end (D99). The second stage seems to recapitulate the first in terms of these measures. Moreover, pairwise distances calculated using two distance metrics (Bray-Curtis and Unifrac) and clustered using average linkage revealed three groups: D01, D03 and D64; D07, D15 and D67; D30, D78 and D99 (Supplementary Fig. S7).

This separation in two stages is most likely caused by the turning procedure performed just before sample D64 was collected. We hypothesize that turning restores to a certain extent conditions prevalent at the beginning of composting. This is in line with the observation that aeration of compost piles impacts microbial activity and temperature^16^,^17^. At the beginning there is an abundance of easily degradable organic nutrients and oxygen^31^. These conditions favor organisms that are aerobic and can most efficiently use the nutrients available, such as members from the order Lactobacillales found in the samples D01 and D64^3^,^4^,^32^. As the degradation progresses, both oxygen and easily degradable nutrients would become progressively scarcer and/or more difficult to access, especially in deeper regions of the chamber. This environment probably constrains the microbial community, and likely increases selection of more specialized microbial populations for degradation of remaining lignocellulosic materials. When turning is performed, oxygen and easily degradable nutrients that were in outer layers of the composting pile become accessible, probably easing the selection constraint. Based on these results we hypothesize that nutrient access and oxygen availability are the major determinants of changes in the composting microbiota composition, which agrees with the concept that accessibility by decomposer organisms is a constraint on biomass decomposition^33^.

### Abundant bacterial species in composting based on shotgun data

At the level of species, using classifications provided by the program MyTaxa^34^ on shotgun DNA reads, the most abundant organisms identified in ZC4 were *Rhodothermus marinus* (2.5% of reads)*, Thermobispora bispora* (2.1%), *Symbiobacterium thermophilum* (1.5%), *Sphaerobacter thermophilus* (1.1%) and *Thermobifida fusca* (0.7%) (Supplementary Table S10). We reconstructed genomes for each of these species and obtained values for marker gene similarity between 86 and 100% (Supplementary Table S11).

All species listed in Supplementary Table S10 online have been found in biomass-degrading environments: *S. thermophilus*^20^,^35^,^36^, *T. bispora*^29^,^37^, *T. fusca*^38^,^39^, *S. thermophilum*^40^,^41^,^42^, and *R. marinus*. For the latter, IMG strains to which our sequences are more similar are described as “compost feedstock-adapted isolates” (Supplementary Table S12)^15^,^20^.

The variation in abundance over time of the five species described above shows interesting patterns (Supplementary Fig. S8). The species with the highest relative abundance is *R. marinus*, and its relative abundance variation agrees with the pattern of phylogenetic diversity variation shown in Fig. 2, albeit with a small lag, both at the beginning and right after the turning procedure (Supplementary Fig. S8a). The other four species present relative abundance variation curves with less obvious patterns (Supplementary Fig. S8b). *T. bispora* and *S. thermophilum* show a pronounced peak on D03 followed by a sharp decrease on the following sampling days, suggesting that conditions at the beginning of the process are favorable to these species. The one species that most deviates from the two-stage pattern is *T. fusca*, since it starts with a low relative abundance, and slowly increases until D64. This suggests that *T. fusca* is able to proliferate only after other species have consumed initially available nutrients. This species is widely identified in lignocellulosic compostings and is well-known as a cellulose and lignin degrader^43^.

Several of the most abundant species identified in ZC4 shotgun DNA reads were also identified as most abundant in metatranscriptome data using classifications provided by the program MyTaxa. Specifically, the top six most abundant species in metatranscriptome data (Supplementary Table S13) are also among the top ten most abundant according to shotgun metagenomics (data not shown). In addition, in the case of *R. marinus,* the variation in abundance over time is very similar in both DNA and mRNA data (Supplementary Fig. S9). These observations give us confidence that our results based on DNA shotgun data reflect actual microbial activity in the composting process.

### Partial genome recovery of a novel bacterial genus guided by 16S data

An analysis of 16S data at the genus level unveiled a microbial composition structure in stark contrast to that of the shotgun DNA just presented (Supplementary Table S14); none of the five most abundant OTUs seem to correspond to the species described in the previous section. We believe that the explanation for this discrepancy is that our analysis of shotgun DNA was entirely based on reference complete genomes. This means that organisms present in the composting process for which there are no complete genomes available (i.e. that are part of the so-called “microbial dark matter”) would not have been identified in the analyses of our shotgun DNA metagenomics dataset. However, the possibility still exists that we can uncover the DNA shotgun fragments of these abundant OTUs by some indirect method. This is what we attempted to do for the most abundant OTU, as described next.

**Figure 3:**
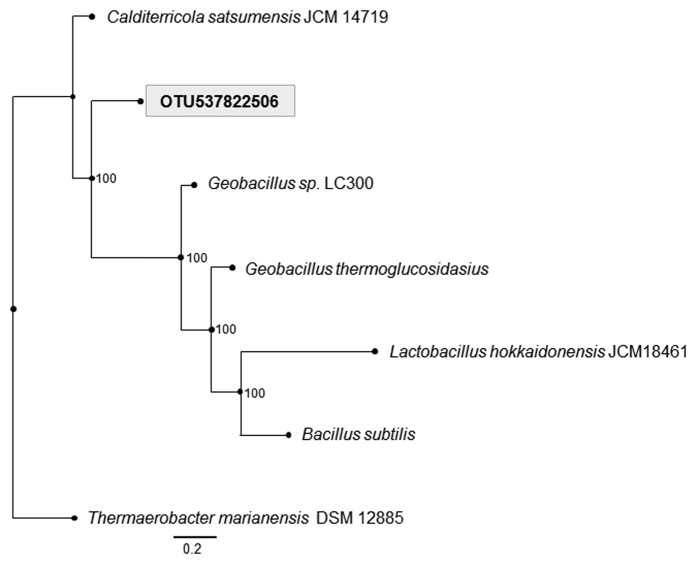
Maximum likelihood phylogenetic tree based on nucleotide sequences of 113 ortholog genes. Node numbers represent bootstrap support. Scale is number of substitutions per site. *Thermaerobacter marianensis* (clostridiales) was chosen as outgroup; all other species are members of the Bacillaceae family.

A BLAST analysis of the 16S fragment that represents OTU537822506 (the most abundant) indicated 100% identity with *Calditerricola yamamurae*, which is a Firmicutes in the family Bacillaceae. There is no complete or even draft genome available for this species. On the other hand, our analysis of shotgun data indicated that in all of our samples there was a fairly large fraction of reads (varying from 8% to 19%) identified as “unassigned Firmicutes”. Moreover, the variation in relative abundance over time for these reads closely followed that of OTU537822506 (data not shown). Based on this evidence, we applied a special assembly strategy (see Supplementary Methods) on these reads and succeeded in recovering what we estimate to be a near-complete (92%) genome of OTU537822506 (2,367,546 bp); average reads coverage for the assembled genome was 51.2 fold (Supplementary Table S15). A phylogenetic analysis based on 113 orthologous genes (Fig. 3) suggests that OTU537822506 is a new genus in the family Bacillaceae.

### Metabolic potential based on metagenomic and metatranscriptomic data

To obtain an overall profile of the gene functions throughout the composting process, coding sequences (CDSs) from ZC4 and ZC3 assembled shotgun reads were classified using COGs (Cluster of Orthologous Groups^44^). Among the 15 functions most abundant in the ZC3 and ZC4 time-series metagenomes (Supplementary Table S16) we found COGs associated with cell maintenance and proliferation (e.g. carbohydrate transport and metabolism; aminoacid transport and metabolism; DNA replication and repair; cell wall biogenesis), signal transduction (e.g. response regulator; histidine kinase) and defense mechanisms (e.g. efflux pump). These functions are present in both ZC3 and ZC4, despite some differences in their relative abundances in the time-series metagenomes.

**Figure 4:**
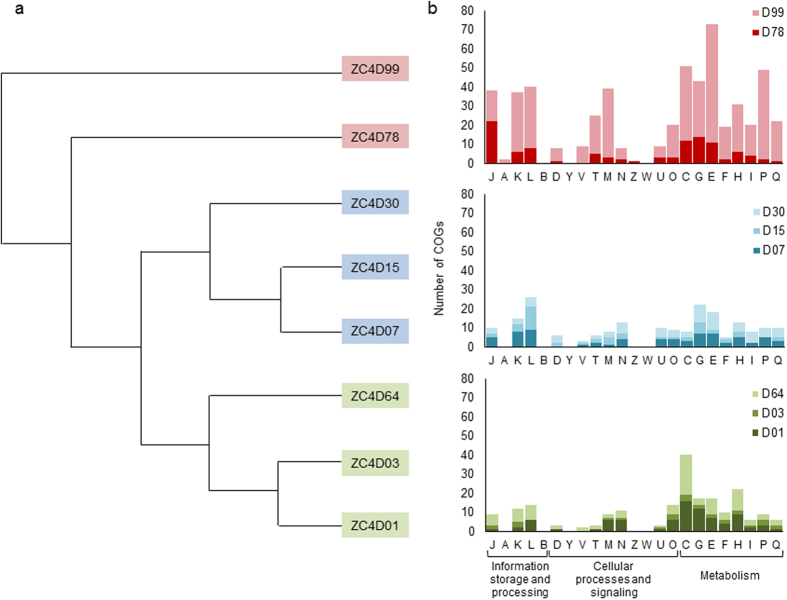
Functional profile of the metatranscriptomes based on COG categories. (**a**) Hierarchical clustering of metatranscriptome CDSs. The tree was generated using an IMG/M tool, selecting the COG pathways method. Samples are referred to by the letter D followed by the collection day. (**b**) Number of COGs (*y* axis) of each COG functional category (*x* axis) that had differential representation, based on relative abundance of CDSs. A COG functional category was considered differentially represented if the relative abundance value for a given sample was at least 1.5 times the interquartile range for the third quartile (i.e., only overrepresented outliers were noted) considering relative abundance values for that category in all samples. Each histogram corresponds to the same-color group of samples to its left.

Next, we analyzed the dynamics of COG functions during the composting process based on ZC4 metatranscriptomic data, to ensure that our results reflect actual functional activity. As a sanity check, we first mapped metatranscriptome reads onto shotgun DNA contigs; the vast majority of reads could be mapped (Supplementary Fig. S10), confirming that by and large the metatranscriptome reads are a subset of the shotgun DNA data. Using COGs, we classified CDSs found in contigs obtained from assembled metatranscriptome reads. Hierarchical clustering of ZC4 samples based on this COG classification resulted in three groups: D01, D03 and D64 (group 1), D07, D15 and D30 (group 2), and D78 and D99 (group 3) (Fig. 4a). These groups correspond to the groups obtained from pairwise distances mentioned previously (Supplementary Fig. S7). These results show that the effects of the turning procedure can be observed both in the microbial composition structure and in the functional profiles.

We interpret the three groups and their COG category differential abundance levels (Fig. 4b) as follows: group 1 represents the beginning (or restart) of the composting process and is characterized by energy production/conversion (COG category C), transport/coenzyme metabolism (H), carbohydrate transport/metabolism (G) and amino acid transport/metabolism (E). These categories include functions related to the microbial metabolism dedicated to degrading and utilizing easily degradable organic nutrients in an environment still rich and available in these compounds. Group 2, representing the middle of the process, is characterized by replication/recombination/repair (L), carbohydrate transport/metabolism (G) and amino acid metabolism (E) categories. At this stage, an intense depolymerizing activity has been occurring as well as the fast utilization of solubilized carbon compounds. When most of the easily degradable nutrients have been consumed, microorganisms able to degrade polymeric carbon become predominant. The higher expression levels of genes in category L could be explained by shifts in microbial composition between the beginning/restart and middle groups, also observed in the taxonomic analysis presented below. Group 3, representing the end of the composting process, is enriched in COGs related to amino acid metabolism (E), energy production/conversion (C), inorganic ion transport/metabolism (P), carbohydrate transport/metabolism (G), and categories dedicated to information, storage, and signaling processes (J, K, L and M). We hypothesize that the oxygen and nutrient limitations favor bacteria specialized in lignocellulosic recalcitrant material (such as lignin) degradation. It is interesting to note that, among abundant CDSs classified in category P, there are sequences encoding superoxide dismutases (SOD). Besides their important role in cellular antioxidant defense, some of these enzymes act as microbial lignin-oxidizers, as shown by manganese-SOD from *Sphingobacterium sp*. T2^45^.

### Transcriptional profile of genes predicted to be involved in lignocellulose degradation

**Figure 5:**
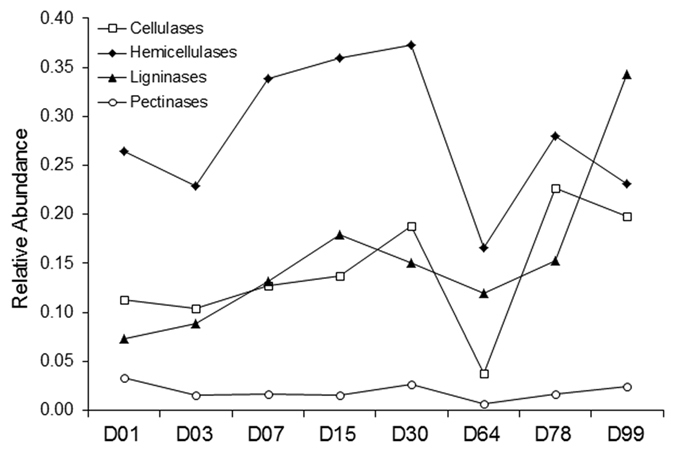
Analysis of CDSs involved in lignocellulosic degradation during ZC4 composting process. Relative abundance (%) of the CDSs annotated as hemicellulases, cellulases, ligninases and pectinases in the metatranscriptome contigs throughout the composting process, according to the IMG-M pipeline. Samples are referred to by the letter D followed by the collection day. Intervals on the horizontal axis do not represent chronological time.

Deconstruction of lignocellulosic biomass can be achieved by diverse mechanisms, and the repertoire of known enzymes and microbial species capable of doing so is rapidly increasing^46^,^47^. We investigated the presence and abundance variation of key lignocellulose-degrading enzymes as given by COG classifications of the ZC4 metatranscriptome dataset. Hemicellulases, cellulases, pectinases, and ligninases were detected (Supplementary Table S17), and variation over time in their respective relative abundance levels indicates that hemicellulose, cellulose, pectin, and lignin are degraded throughout, with turning causing a temporary slowdown of the degradation process (Fig. 5). This is in agreement with the two-stage process mentioned previously. Note that ligninase abundance reaches a peak only at the end (D99). These results indicate that lignocellulosic biomass deconstruction occurs synergistically and sequentially, with hemicellulose being degraded preferentially to cellulose and lignin, as also seen in previous studies^16^,^23^,^46^.

To explore the repertoire of the lignocellulose-degrading enzymes in more detail, we compared all CDSs of ZC4 metatranscriptome against sequences in the CAZy database^48^. We found hits to six carbohydrate-active enzyme classes and associated modules: glycoside hydrolases (GHs) (29.5–34.2%), carbohydrate-binding modules (CBM) (21.5–39.1%), glycosyl transferases (GTs) (15.6–27.1%), carbohydrate esterases (CEs) (10.3–15.3%), polysaccharide lyases (PLs) (0.8–2.4%) and auxiliary activities (AAs) (1.6–8.4%) (Supplementary Fig. S11). Among the most abundant enzymes and associated domains, we highlight the surface-layer homology (SLH) domain and CBM50. The SLH domain is present in cellulosome scaffolding proteins anchoring the degradation complex to the bacterial cell surface^49^ and in some secreted GHs (such as xyn-b39 and endo1,4β glucanase CelD)^50^,^51^. The CBM50 domain, which binds N-acetylglucosamine residues in bacterial peptidoglycans and in chitin, is found attached to enzymes from GH18, GH19, GH23, GH24, GH25, and GH73 families^48^. Along with these six GH families, endoglucanases (GH5 and GH9) and exo-cellobiohydrolases (GH6, GH48 and GH7), necessary to the complete hydrolysis of cellulose, are also represented in our metatranscriptome dataset (Supplementary Tables S17 and S18). Putative cellulase-encoding enzymes, mostly belonging to families GH5 and GH9, were also observed in bagasse, switchgrass-adapted compost, and in rice straw-adapted microbial consortia metagenomes^19^,^23^,^30^.

The ZC4 metatranscriptome also encodes ligninolytic enzymes and lytic polysaccharide mono-oxygenases (AA enzymes). The majority of these enzymes, found in all samples, belongs to classes AA2 (7–28% of all AAs) and AA6 (32–66%), followed by moderate amounts of AA3, 4, 7, 9 and 10 (Table S18). Sample D99 contains the highest number of CDSs assigned to this class.

Notably, we did not find any CDSs that could be annotated as fungal laccases, manganese peroxidases, versatile peroxidases, and lignin peroxidases. Wang *et al*.^30^, studying biomass-degrading consortia isolated from composting, have not found them either. This is additional evidence that lignocellulose degradation in a thermophilic composting process is primarily or even exclusively the result of bacterial enzymatic activity. Thus, lignocellulolytic enzymes of bacterial origin may be of special interest in industrial applications in which fungal enzymes cannot be used^30^,^52^,^53^.

### Taxonomic assignment of CDSs related to lignocellulosic biomass degradation

**Figure 6:**
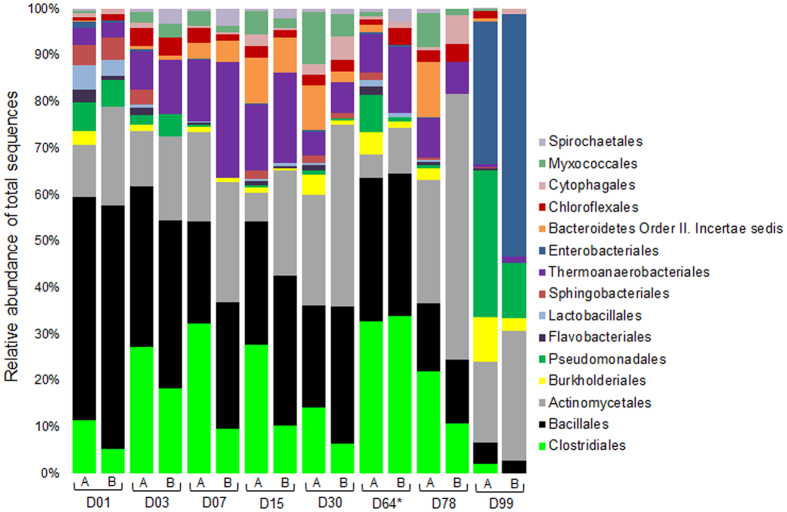
Taxonomic profile of the ZC4 metatranscriptome. Columns labeled by A show taxonomic profiles considering all CDSs annotated with COGs. Columns labeled by B show taxonomic profiles considering CDSs annotated with COGs related to plant biomass (lignocellulose and pectin) degradation. The CDSs were classified with program myTaxa. Samples are referred to by the letter D followed by the collection day. The asterisk indicates one day after the turning procedure.

Here we revisit the question of which microorganisms are mostly responsible for lignocellulosic biomass degradation based on metatranscriptome data. Figure 6 shows the most abundant orders. Except for D99, members from the orders Bacillales, Clostridiales, Actinomycetales, and Thermoanaerobacterales abound throughout the composting process, and most CDSs related to plant biomass degradation were classified as belonging to members of these orders. Actinomycetales are commonly found in compost, particularly in the thermophilic and mature stages^11^,^16^,^25^,^54^, and also in thermophilic microbial consortia enriched from compost^30^,^55^. Indeed, in our data the relative abundance of CDSs assigned to this order involved with lignocellulosic biomass degradation is largest at D30 and D78 (34.4% and 49%, respectively). Members of orders Clostridiales and Bacillales are known to have genes encoding enzymes involved in cellulose and hemicellulose degradation and were implicated as the major plant biomass degrading microbes in peat swamp forests^56^,^57^.

We can also observe orders that seem to play roles related to plant biomass deconstruction only during certain stages of the process. Members of Bacteroidetes Order II. incertae sedis are clearly present in the first stage (from D03 to D30), but fade thereafter. Members of Lactobacillales are more active at the beginning (D01) and after turning (D64). At the end of the process (D99), besides Actinomycetales, members of Enterobacteriales and Pseudomonadales are most abundant and seem to contribute to the continued degradation of remnant material. Enterobacter spp. are commonly described at the early stages of composting and are associated with lower temperatures ( < 60 °C)^32^,^58^. Indeed, high levels of CDSs assigned to this bacterial order become more prominent when temperature decreases to less than 50 °C (D99). Most (69.2%) of the D99 Enterobacteriales CDSs were assigned to a single species, *Klebsiella pneumoniae*, which is known to perform cellulose and hemicellulose degradation, nitrogen fixation, and has been associated with wood, termite gut, and composting ecosystems^59^,^60^. Other studies also recorded the marked presence of members of the orders Enterobacteriales, especially *Klebsiella* species, in wheat straw degrading microbial consortia^61^,^62^.

A COG-based analysis of the reconstructed genome of OTU537822506 revealed two CDSs related to lignin breakdown (COG1496 and COG2132-multicopper oxidases) and one to pectin hydrolysis (COG3866-pectate lyase). CAZy analysis revealed 63 genes encoding 12 CBMs, 11 GHs, 17 GTs, 10 CEs, 1 PL and 4 AAs (Supplementary Table S19). These results indicate that the genome of this organism encodes enzymes related to bioconversion of all components in plant biomass, with emphasis on hemicellulose degradation (represented by the following CAZy families: CBM13, CBM37, GH11, GH16, CE4 and CE1). Variation over time of the shotgun reads used to reconstruct the genome as well as metatranscriptome reads mapped to the OTU537822506 genome show high levels at the beginning of composting (Supplementary Fig. S12), suggesting that this bacterial species is more active during the initial stages.

## Conclusions

We have presented a detailed study of a thermophilic composting process, from a microbial molecular standpoint. Thanks to time-series sampling and high-throughput sequencing, we were able to observe the variation of the microbiota that orchestrate the composting process as well as the variation of their enzymatic activities.

**Figure 7:**
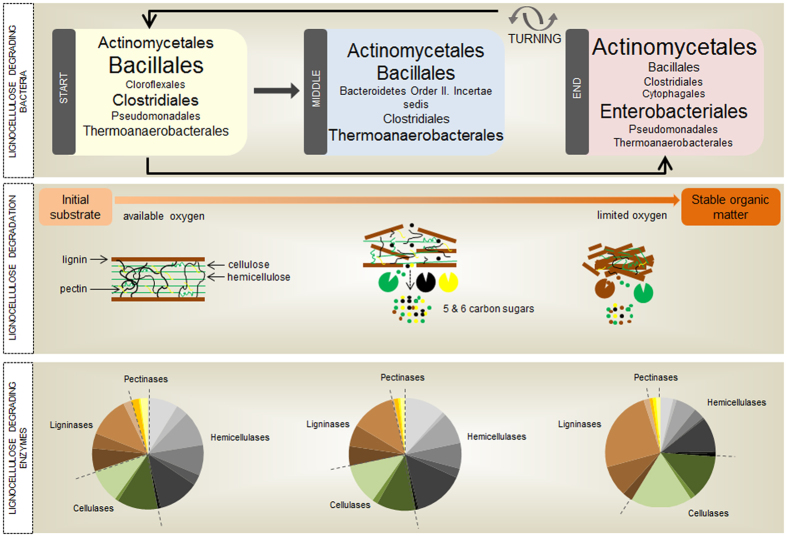
Molecular overview of a thermophilic composting operation. The top panel shows the three stages identified in this work: start (D01, D03 and D64), middle (D07, D15 and D30), and end (D78 and D99). The box for each stage shows the microbiota composition at the order level. The relative abundance of microorganisms is roughly proportional to the font size, and only orders with relative abundance ≥2.5% are shown. The middle panel depicts the degradation process, with vertical correspondence to the stages shown in the top panel. The bottom panel shows relative abundance of COG functions associated to lignocellulolytic enzymes, also with vertical correspondence to the stages shown in the top panel. The results in this figure were based on ZC4 metatranscriptomes.

The results described here, together with other studies on composting microbiota^16^,^23^,^57^, allow us to propose a core group of bacterial microorganisms and metabolic functions primarily responsible for lignocellulosic biomass degradation, as sketched in Fig. 7.

The impressive variety of bacterial microorganisms and metabolic functions so far found to be active in thermophilic composting warrant this engineered ecosystem as valuable source for continued survey of new bacteria and metabolic functions adapted to an extreme and complex environment. We have identified and obtained a near-complete genome of an organism that plays a role in biomass degradation and likely represent a new genus. This is probably just the tip of an iceberg, and additional microbial molecular studies of composting systems should uncover a whole treasure trove of new microorganisms and their enzymes useful for environment management and biofuel production.

## Methods

### Composting

The composting process was carried out at the composting facility of São Paulo Zoo Park, São Paulo, Brazil (23°38′56.9″S 46°37′18.7″W) with minor modifications from a previously described procedure^63^. The facility has several 8 m^3^ open concrete cells (1.6 × 2.0 × 2.5; height × width × depth) and is designed to compost four tons/day of all organic waste produced in the park comprising mainly shredded tree branches, leaves and grass from the maintenance of park green areas, plus manure, beddings and food residues from about 400 species of zoo animals (mammals, avian and reptiles). The facility area is protected from sunshine and rain. To build the composting pile the substrates were layered within the concrete cell to roughly reach a Carbon:Nitrogen (C/N) ratio of 30:1. Adequate aeration of the pile was maintained by aeration pipes placed at the bottom of concrete cell and by arranging the substrates to limit excessive compaction. Daily reported temperatures of the composting piles were averages of measurements taken at the four edges and in the center. Proper humidity was maintained by watering the composting pile. When temperature dropped below 55 °C, usually after ~60–65 days of composting, the pile was turned using a BobCat skid-steer loader (Bobcat Company, USA) to restore aeration and thermophilic conditions. After ~100 days, the composting process within the concrete cell was considered finished, the compost material was removed and aged for an additional 10 days in windrows.

### Sample collection

Samples were collected throughout the composting process from two cells named ZC3 and ZC4, which were built, respectively, on 06/27/2011 and 08/05/2013. After completion of the composting pile, samples were collected on days 1, 30, 64, 78 and 99 for ZC3, and on days 1, 3, 7, 15, 30, 64, 67, 78 and 99 for ZC4. Samples are referred to by the letter D followed by the collection day (e.g. D01 is day one). The turning procedure was performed on day 65 for ZC3 and on day 63 for ZC4. To obtain a representative sample of the pile at each sampling day, sub-samples were collected from five different points (at all depths in the four edges and center)^13^. The five sub-samples (~100 g each) were pooled, thoroughly mixed, distributed into sterile 50 mL Falcon tubes and immediately frozen in dry ice before being transported to the laboratory, where they were stored at −80 °C until DNA extraction or chemical analysis. Samples for moisture analysis and pH were freshly processed. For total RNA extraction, ~1 g of each composite sample was immediately transferred to 15 mL Falcon tubes containing the twice the volume of LifeGuard Soil Preservation Solution (MoBio Laboratories, USA) and the suspensions were kept at room temperature until RNA extraction no longer than 1 week. Tubes and spatulas were previously treated with RNAse Away (Life Technologies, USA).

### Physicochemical analyses

Temperature was measured at a depth of 60 cm from the top of pile using digital thermometer with long-handled stainless-steel probe in the four edges and center of the pile. Moisture content determination was performed by microwave oven drying as previously described^63^. The pH was determined from suspensions of fresh compost samples in 0.9% sodium chloride using a pH electrode. Total Carbon, Hydrogen and Nitrogen content were determined using PerkinElmer 2400 series II CHNS/O analyzer (Perkin-Elmer, USA). Elemental analysis (Al, Fe, Mg, P, As, Cd, Cr, Cu, K, Ni, Pb, Se and Zn) was performed by inductively-coupled plasma optical emission spectrometry using Spectro Arcos ICP-OES analyzer (Spectro, Germany).

### DNA extraction and shotgun metagenomic library preparation

Metagenomic DNA extraction was performed with MoBio DNA Power Soil kit. Prior to DNA extraction, −80 °C stored samples were subjected to lyophilization, and ~2 g lyophilized material were thoroughly grounded in a sterile mortar. Purified metagenomic DNA was subjected to a final clean-up step using QIAamp mini spin columns (Qiagen, USA) and stored −80 °C. DNA purity and concentration were evaluated on a ND-1000 spectrophotometer (Nano Drop Technologies, USA) at 260 nm, 280 nm and 230 nm. Further quantification was performed with Quant-iT Picogreen dsDNA assay kit (Life Technologies, USA). DNA integrity was examined with DNA 7500 chip using 2100 Bioanalyzer. DNA samples were mostly enriched in fragments higher than 10 kbp.

Shotgun metagenomic libraries were prepared using an Illumina Nextera DNA library preparation kit (Illumina, Inc., USA) with total DNA input of 20–35 ng. The resulting DNA fragment libraries were cleaned up with Agencourt AMPure XP beads (Beckman Coulter, Inc., USA) and fragment size within the range of 400–700 bp was verified by running in the 2100 Bioanalyzer using Agilent High Sensitivity DNA chip.

### 16S Metagenomic Sequencing Library Preparation

PCR reactions were performed using a primer pair based on the sequences of primers S-D-Bact-0341-b-S-17 and S-D-Bact-0785-a-A-21^64^ for amplification of the variable regions V3 and V4 of 16S rRNA gene and including adapters as suggested on the Illumina workflow for 16S Metagenomic Sequencing Library Preparation. Forward primer 341F, 5′-TCGTCGGCAGCGTCAGATGTGTATAAGAGACAGCCTACGGGNGGCWGCAG and reverse primer 785R, 5′-GTCTCGTGGGCTCGGAGATGTGTATAAGAGACAGGACTACHVGGGTATCTAATCC were used in PCR reactions (25 μL) performed with 12.5 ng of metagenomic DNA samples, KAPA HiFi HotStart ReadyMix (Kapa Biosystems, USA), 200 nM of each primer at 95 °C for 3 min followed by 25 cycles at 95 °C for 30 sec, 55 °C for 30 sec and 72 °C for 30 sec plus 72 °C for 5 min. Amplicon expected size of ~550 bp was verified with Bioanalyzer DNA 1000 chip. PCR product cleanup was performed with AMPure XP beads (Beckman Coulter, Inc., USA). Dual indexes were attached using the Nextera XT Index Kit and after a second round of PCR cleanup with AMPure XP beads, V3–V4 16S indexed amplicons libraries were validated by running on a Bioanalyzer High Sensitivity DNA chip to verify the expected size of ~630 bp.

### RNA extraction, rRNA depletion and RNA-seq library preparation

Total RNA was extracted from compost samples stored in LifeGuard Soil Preservation Solution (MoBio Laboratories, USA) using RNA MoBio PowerSoil Total RNA isolation kit. Extracted total RNA was stored at −80 °C. RNA purity and concentration were evaluated on a ND-1000 spectrophotometer (Nano Drop Technologies, USA) at 260 nm, 280 nm and 230 nm. Further quantification was performed with Quant-iT Ribogreen RNA Assay kit (Life Technologies, USA). RNA integrity number (RIN) was determined using RNA 6000 Nano kit in the 2100 Bioanalyzer (Agilent Technologies, USA). Only samples with RIN scores >7.5 were further processed. For removal of contaminant genomic DNA, total RNA samples were DNAse I-treated with Illustra RNASpin mini kit (GE Healthcare Life Sciences, USA) and re-evaluated regarding their RIN scores. Complete removal of genomic DNA was confirmed by PCR with primers for variable region V3 and V4 of 16S rRNA gene. Depletion of rRNA was performed using a Ribo-Zero rRNA Removal kit/Bacteria (Epicentre, USA) and 5 μg of DNAse-treated RNA. The final purification of the rRNA-depleted RNA (90 μL) was performed by adding 90 μL of RNase-free water, 18 μL of 3M-sodium acetate, 2 μL of glycogen (10 mg/mL) and 600 μL of cold 100% ethanol. After 2 h incubation at −20 °C, rRNA-depleted RNA was collected by centrifugation at 16,000 ×g for 30 min at 4 °C. After two washes with cold 70% ethanol, the rRNA-depleted RNA pellet was dried at room temperature for 5 min and immediately used for RNA-seq library preparation with ImProm-II Reverse Transcription System (Promega, USA) and TruSeq RNA Library Preparation Kit v2 (Illumina, Inc., USA). Complete depletion of rRNA was verified using Bioanalyzer RNA 6000 Pico chip. Average fragment size of ~300 bp of the RNA-seq libraries were verified using a Bioanalyzer High Sensitivity DNA chip.

### Sequencing

Quantification of Illumina sequencing libraries with KAPA Library Quantification Kit, normalization, and pooling were performed following standard protocols for sequencing in the Illumina MiSeq platform. Pooled libraries were subjected to 2–3 runs using the MiSeq Reagent kit v2 (500-cycle format, paired-end (PE) reads) and resulting sequences for each library were combined. RNA-seq libraries were also run on a single-sequencing lane on Illumina HiSeq2500 (200-cycle format) using TruSeq SBS v3 kits, and resulting sequences were combined with the MiSeq PE sequences. On average, Illumina PE read1 and read2 presented, respectively, >80% and >75% of bases with quality score at least 30 (Q30).

DNA samples purified from ZC3 chamber were also submitted to pyrosequencing following standard Roche 454 GS FLX Titanium protocols (Roche Applied Science) as previously described^13^.

### Bioinformatics analyses

We processed raw PE sequencing reads and 454-reads in various ways. Unassembled reads were submitted for automatic processing to the MG-RAST metagenomics analysis server^65^,^66^ using their default quality control pipeline. We also submitted data to the IMG/M annotation pipeline^67^ using the following steps. Raw PE sequencing reads data were quality-filtered to remove reads shorter than 50 bp or reads with average quality score lower than phred 20 using SICKLE^68^, FASTX-Toolkit (http://hannonlab.cshl.edu/fastx_toolkit/) and fastQC (http://www.bioinformatics.babraham.ac.uk/projects/fastqc/). Assembly of quality-filtered PE reads and 454-reads for each shotgun metagenomic or RNA-seq library was performed with SOAPdenovo2^69^ followed by assembly quality checking using QUAST^70^. The resulting sets of contigs were submitted to IMG/M^67^.

Methods employed in the analyses of metagenomic and metatranscriptomic sequences, taxonomic classification based on shotgun reads^34^,^65^,^66^ and 16S rRNA amplicon sequencing^71^, microbial community and diversity analyses, reconstruction of bacterial genomes, genome annotation and phylogenetic tree construction are presented in Supplementary Methods.

## Additional Information

**Accession codes:** All sequencing data described in this work comprising unassembled sequence reads, resulting sets of contigs (including singlets) and 16S rRNA V3-V4 amplicon sequences are available at MG-RAST, IMG/M or SRA databases under accession numbers listed in Supplementary Table S1. The Whole Genome Shotgun project related to the genome of OTU537822506 has been deposited at DDBJ/ENA/GenBank under the accession LWLU00000000. The version described in this paper is version LWLU01000000.

**How to cite this article**: Antunes, L. P. *et al*. Microbial community structure and dynamics in thermophilic composting viewed through metagenomics and metatranscriptomics. *Sci. Rep.* **6**, 38915; doi: 10.1038/srep38915 (2016).

**Publisher's note:** Springer Nature remains neutral with regard to jurisdictional claims in published maps and institutional affiliations.

## Supplementary Material

Supplementary Tables

Supplementary Figures

Supplementary Methods

## Figures and Tables

**Table 1:** Description of samples collected from ZC4 composting cell.

Day of collection	Sample ID Collection Date	Average Temperature^a^ (°C)	Δ Temperature^b^ (°C)	pH	Moisture (%)
1	D01 08/06/2013	66.2 ± 7.5	18	6.0	71.0
3	D03 08/08/2013	68.4 ± 2.6	6	5.5	67.0
7	D07 08/12/2013	70.8 ± 3.5	9	6.4	70.3
15	D15 08/20/2013	68.2 ± 5.8	14	6.9	63.8
30	D30 09/04/2013	69.5 ± 2.3	5.6	6.9	61.8
64*	D64 10/08/2013	57.9 ± 8.9	21.2	6.8	60.7
67	D67 10/11/2013	70.5 ± 4.8	10.7	7.4	n.d.
78	D78 10/22/2013	61.2 ± 3.2	8.0	7.8	58.6
99	D99 11/12/2013	47.8 ± 3.3	8.3	7.9	58.7

^a^Mean plus standard deviation of temperature measurements taken at the four edges and in the center of the composting pile.

^b^Variation between lower and higher temperature measurements. n.d., not determined.

^*^Turning was performed on day 63.
