# Supplementary material for: Microbial community structure and dynamics in thermophilic composting viewed through metagenomics and metatranscriptomics
Source: Sci Rep. 2016 Dec 12;6:38915. doi: 10.1038/srep38915 (PMC5150989; doi:10.1038/srep38915)
Supplement: Supplementary Tables [file srep38915-s1.pdf]

Supplementary Tables

Title: Microbial community structure and dynamics in thermophilic composting viewed through metagenomics and metatranscriptomics

Authors:
Luciana Principal Antunes[1]
Layla Farage Martins[1]
Roberta Verciano Pereira[1]
Andrew Maltez Thomas[1,2]
Deibs Barbosa[1,2]
Leandro Lemos Nascimento[1,2]
Gianluca Major Machado Silva[1,2]
Livia Maria Silva Moura[1,2]
George Willian Condomitti Epamino[1,2]
Luciano Antonio Digiampietri[3]
Karen Cristina Lombardi[1]
Patricia Locosque Ramos[4]
Ronaldo Bento Quaggio[1]
Julio Cezar Franco de Oliveira[5]
Renata Castiglioni Pascon[5]
João Batista da Cruz[4]
Aline Maria da Silva[1,2,*]
João Carlos Setubal[1,2,6,*]

Affiliations:
[1] Departamento de Bioquímica, Instituto de Química, Universidade de São Paulo, São Paulo, Brazil
[2] Programa de Pós-Graduação Interunidades em Bioinformática, Universidade de São Paulo, São Paulo, Brazil
[3] Escola de Artes, Ciências e Humanidades, Universidade de São Paulo, São Paulo, Brazil
[4] Fundação Parque Zoológico de São Paulo, São Paulo, Brazil
[5] Departamento de Ciências Biológicas, Universidade Federal de São Paulo, São Paulo, Brazil
[6] Biocomplexity Institute of Virginia Tech, Blacksburg, VA, USA
[*] These authors shared senior authorship

Correspondence and requests for materials should be addressed to: J.C.S. (setubal@iq.usp.br) or A.M.D.S. (almsilva@iq.usp.br)

Table S1. Accession numbers for sequencing datasets

ZC4 Samples	Unassembled reads MG-Rast ID		Contigs IMG-M Taxon Object ID
Shotgun metagenomics (MiSeq PE 500-cycle)			
	Read 1	Read 2	
D01	4556395.3	4556396.3	3300005064
D03	4556397.3	4556398.3	3300005063
D07	4549529.3	4549530.3	3300002205
D15	4556399.3	4577137.3	3300005060
D30	4556401.3	4556402.3	3300005061
D64	4556403.3	4556404.3	3300005072
D67	4556405.3	4556406.3	3300005059
D78	4565311.3	4565312.3	3300003647
D99	4565313.3	4565314.3	3300003648
Metatranscriptomics (MiSeq PE 500-cycle + HiSeq PE 200-cycle)			
D01	4604922.3	4604923.3	3300003896
D03	4604924.3	4604925.3	3300003897
D07	4604926.3	4604927.3	3300003898
D15	4604928.3	4604929.3	3300003912
D30	4604930.3	4604931.3	3300003900
D64	4604932.3	4604933.3	3300003913
D78	4604934.3	4604935.3	3300003899
D99	4604936.3	4604937.3	3300003901

ZC3 Samples	Unassembled reads MG-Rast ID		Contigs IMG-M Taxon Object ID
Shotgun metagenomics (Roche 454 GS FLX Titanium technology)			
ZC3 A D01	4500077.3		3300000505
ZC3 A D30	4500078.3		3300000483
ZC3 A D64	4500079.3		3300000489
ZC3 A D78	4500080.3		3300000476
ZC3 A D99	4500081.3		3300000514
Shotgun metagenomics (MiSeq PE 500-cycle)			
	Read 1	Read 2	
ZC3 B D01	4533999.3	4534000.3	3300002194
ZC3 B D30	4533996.3	4533995.3	3300002193
ZC3 B D64	4533998.3	4534001.3	3300002192
ZC3 B D78	4533994.3	4533997.3	3300002210
ZC3 B D99	4532702.3	4532703.3	3300002196

	16S rRNA V3-V4 amplicon sequences SRA access numbers
ZC3 and ZC4 samples	SRP068633

Table S2. Description of samples collected from ZC3 composting cell

Day of collection	Sample ID<br>Collection Date	Average Temperature[a] (°C)	Δ Temperature[b] (°C)	pH
1	D01<br>28/06/2011	60.4± 6.6	14	5.0
30	D30<br>27/07/2011	58.0 ± 6.5	15	7.9
64*	D64<br>30/08/2011	53.2 ± 6.5	16	7.0
78	D78<br>13/09/2011	56.6 ± 9.6	24	7.0
99	D99<br>04/10/2011	49.4 ± 10.3	25	7.0

[a]Mean plus standard deviation of temperature measurements taken at the four edges and in the center of the composting pile.[b]Variation between lower and higher temperature measurements. *Turning was performed on day 65.

Table S3. Chemical characteristics of ZC4 samples

Samples	C:H:N	C	H	N	Al	Fe	Mg	P	As	Cd	Cr	Cu	K	Ni	Pb	Se	Zn
		% (dry weight)							mg/kg (dry weight)								
D01	16:02:01	37.6	5.6	2.4	1.5	1.1	0.2	0.4	<0.05	<0.01	14.9	19.0	1.0	<0.01	<0.04	<0.06	2.4
D15	18:02:01	38.9	5.2	2.2	1.1	1.7	0.3	0.3	<0.05	<0.01	25.6	15.5	1.3	<0.01	<0.04	<0.06	4.5
D30	14:02:01	33.9	4.4	2.4	1.2	1.8	0.3	0.5	<0.05	<0.01	23.4	23.4	1.4	<0.01	<0.04	<0.06	8.5
D64	15:02:01	35.1	4.5	2.3	1.2	1.3	0.3	0.4	<0.05	<0.01	20.2	24.5	1.4	<0.01	<0.04	<0.06	9.4
D78	13:02:01	29.0	3.8	2.3	1.3	1.1	0.3	0.5	<0.05	<0.01	13.3	31.9	1.8	<0.01	<0.04	<0.06	20.4
D99	11:01:01	32.2	4.1	2.8	1.3	1.4	0.3	0.6	<0.05	<0.01	16.5	23.9	1.7	<0.01	<0.04	<0.06	15.5

Table S4. Chemical characteristics of ZC3 samples

Samples	C:H:N	C	H	N	Al	Fe	Mg	P	As	Cd	Cr	Ni	Pb	Se	Zn
		% (dry weight)							mg/kg (dry weight)						
D01	14:02:01	35.45	5.2	2.5	13.2	1.1	0.5	0.6	<0.3	<0.02	50.6	<0.03	<0.10	<0.2	207.3
D30	15:02:01	34.96	4.5	2.3	2.5	1.0	0.6	0.7	<0.3	<0.02	<0.03	<0.03	<0.10	<0.2	211.4
D64	15:02:01	37.45	5.1	2.5	0.5	0.3	0.3	0.7	<0.01	<0.03	<0.02	<0.01	<0.06	<0.01	91.5
D78	14:02:01	35.27	4.6	2.6	1.1	0.4	0.4	0.9	<0.02	0.01	<0.004	<0.04	<0.07	<0.01	191.8
D99	12:02:01	32.46	4.4	2.8	1.02	0.5	0.3	1.0	<0.03	<0.01	<0.04	<0.04	<0.07	<0.01	173.7

Table S5. Sequencing and assembly metrics of ZC4 samples

Parameter	D01	D03	D07	D15	D30	D64	D67	D78	D99
Shotgun metagenomics									
Total number of paired-end reads (read1+read2)	8213864	9340402	9113124	14438720	9653864	14469070	8369838	22490204	16647048
Mean read length (bp)	205	228	179	175	197	205	186	150	170
Metagenome size of unassembled reads (bp)	3385195302	4277689176	3279801076	5095916642	3822299536	5947512254	3133151522	6839411382	5690236784
Metagenome size of assembled reads (bp)	243754413	219282386	151588766	177815419	190856909	382856416	161189624	237497447	269689437
Number of contigs†	568660	487725	336757	391739	421533	883951	389331	517038	596836
Largest contig (bp)	7548	6098	7492	5534	10147	8897	4679	9038	8020
Mean contig length (bp)	428	449	450	453	452	433	414	459	451
N50 contig length (bp)	408	428	424	430	429	412	396	436	431
Number of singletons	5637490	6533285	5892230	10166739	6576682	9693272	6178591	15893536	11785414
RNA-seq									
Total number of paired-end reads (read1+read2)	14937982	14873258	14524876	11978457	9245877	17514259	-	9564842	7416187
Mean read length (bp)	113	114	116	113	141	114	-	112	139
Metatranscriptome size of unassembled reads (bp)	3405044922	3402767494	3385787392	2731899029	2617571479	4015710801	-	2162396674	2080659729
Metatranscriptome size of assembled reads (bp)	43615715	46631239	51298791	40471157	40860838	58172101	-	23091753	19965991
Number of contigs†	79492	80281	80114	56979	80028	106273	-	44903	35669
Largest contig (bp)	12283	14247	13025	20141	15199	13323	-	7632	14074
Mean contig length (bp)	548	580	640	710	510	547	-	514	559
N50 contig length (bp)	544	588	679	814	498	540	-	504	558
Number of singletons	10982633	10180001	8689757	5310469	5619976	11888201	-	5770788	5607829
16S rRNA V3-V4 amplicon									
Total number of paired-end reads	1014282	2623129	1495244	2082961	784604	2376934	1364487	1862316	1285268
Mean read length (bp) ± SD	457 ± 13	456 ± 17	453 ± 16	454 ± 16	453 ± 15	456 ± 17	453 ± 20	452 ± 25	454 ± 18

† only contigs with length ≥ 300 bp

Table S6. Sequencing and assembly metrics of ZC3 samples

Shotgun metagenomics										
Parameter	D01		D30		D64		D78		D99	
	(A)	(B)	(A)	(B)	(A)	(B)	(A)	(B)	(A)	(B)
Total number of reads	520074	2270264	737772	2145650	771427	1265240	1063197	2169689	711081	1509658
Mean read length (bp)	441	170	456	168	473	220	460	223	455	229
Metagenome size of unassembled reads (bp)	229379927	772598899 *	336965302	722769482 *	364908522	557561329 *	489666559	970119798 *	323585594	691581550 *
Metagenome size of assembled reads (bp)	99374672	91049842	154178375	75162465	151490080	72084723	221702398	149615890	134924924	103783150
Number of contigs†	208638	192597	335023	172923	319374	163444	480843	348389	283713	229445
Largest contig (bp)	6902	11253	5323	6137	6606	5653	4354	5960	9563	9198
Mean contig length (bp)	476	472	460	434	474	441	461	429	475	452
N50 contig length (bp)	458	435	457	404	465	414	459	409	465	426
Number of singletons	258195	1335560	417142	1503430	449609	841642	628294	1452236	379900	952039

16S rRNA V3-V4 amplicon					
Parameter	D01	D30	D64	D78	D99
Total number of paired-end reads (read1+read2)	3796538	4862931	2508305	4436611	2661313
Mean read length (bp) ± SD	453 ± 13	453 ± 13	454 ± 16	457 ± 13	457 ± 13

(A) Roche 454 GS FLX Titanium technology

(B) Paired-end reads from Illumina-MiSeq (500-cycle)

* sum of paired-end reads: read1 and read2

† only contigs with length ≥ 300 bp

Table S7. Domain distribution in ZC4 compost time-series samples

Database	M5NR								
Metagenome<br>Domain	D01<br>%	D03<br>%	D07<br>%	D15<br>%	D30<br>%	D64<br>%	D67<br>%	D78<br>%	D99<br>%
Bacteria	90.47	90.50	88.44	89.19	89.72	86.80	87.70	88.35	87.60
Archaea	0.27	0.29	0.48	0.39	0.28	0.46	0.37	0.69	0.35
Eukaryota	0.31	0.27	0.15	0.19	0.31	0.31	0.12	0.13	0.12
Viruses	0.17	0.13	0.07	0.05	0.08	0.09	0.09	0.21	0.11
Unclassified	8.78	8.81	10.86	10.18	9.61	12.34	11.72	10.62	11.82

Unassembled reads annotated on MG-RAST were analyzed using the classification tool based on M5NR (60% minimum identity) with maximum e- value cutoff of $1x10^{-5}$ and minimum alignment length of 50 bp.

Table S8. Domain distribution in ZC3 compost time-series samples

Database	M5NR				
Metagenome<br>Domain	D01<br>%	D30<br>%	D64<br>%	D78<br>%	D99<br>%
Bacteria	84.86	89.62	88.54	87.79	89.18
Archaea	1.51	0.61	0.74	0.54	0.48
Eukaryota	0.17	0.53	0.31	0.28	0.28
Viruses	0.05	0.12	0.12	0.14	0.32
Unclassified	13.41	9.12	10.30	11.25	9.75

Unassembled reads annotated on MG-RAST were analyzed using the classification tool based on M5NR (60% minimum identity) with maximum e- value cutoff of $1x10^{-5}$ and minimum alignment length of 50 bp.

Table S9. Abundant genera in ZC4 metagenome within selected orders according 16S rRNA Amplicon dataset

Order	Genus	% of classified OTUs within the respective orders								
		D01	D03	D07	D15	D30	D64	D67	D78	D99
Clostridiales	*Clostridium*	7.9	4.8	1.9	2.6	3.0	20.9	2.7	7.4	10.2
	Symbiobacterium	5.9	31.0	34.0	40.6	12.2	11.5	15.4	13.2	24.0
	Tepidimicrobium	1.1	12.3	7.9	4.8	11.1	6.4	3.8	3.8	3.5
	Thermaerobacter	0.5	1.5	7.1	38.7	27.4	1.1	14.9	20.0	20.2
	Ethanoligenens	0.4	5.4	30.3	0.5	0.2	0.3	0.9	0.0	0.1
	Acetivibrio	0.3	1.6	0.3	0.7	3.4	5.1	9.1	8.8	8.6
Bacillales	*Geobacillus*	43.5	30.4	14.4	5.8	14.2	3.2	4.0	5.1	10.8
	Ureibacillus	13.6	25.0	8.1	7.6	5.6	27.1	9.4	9.2	10.2
	Bacillus	6.2	3.5	4.7	3.8	10.2	14.7	15.5	12.4	11.5
	Paenibacillus	6.0	2.3	2.3	2.4	1.9	3.6	2.4	1.7	4.5
	Thermobacillus	3.7	3.0	3.0	23.4	7.8	0.4	2.2	3.3	4.2
	Planifilum	1.7	8.5	31.4	12.8	31.1	1.4	11.1	10.9	6.0
	Lysinibacillus	1.4	1.0	1.5	1.9	0.6	5.7	7.5	6.7	6.9
Actinomycetales	*Thermopolyspora*	17.1	22.9	24.0	16.7	21.4	12.3	16.5	7.8	8.6
	Thermobispora	13.7	21.3	20.5	10.6	10.3	4.5	7.2	2.3	3.6
	Streptomyces	9.7	6.0	2.9	4.8	2.3	2.1	1.9	1.6	2.2
	Actinomadura	7.0	3.2	0.8	1.3	1.4	3.7	4.3	5.0	6.6
	Thermomonospora	1.9	2.8	6.3	5.6	7.5	12.3	8.0	12.3	13.7
	Thermocrispum	1.4	0.9	1.8	4.5	3.1	8.9	5.9	2.2	3.4

Table S10. Most abundant bacterial species in ZC4 (all samples) that could be classified by program MyTaxa

Species	Order	% of classified reads
Rhodothermus marinus	Bacteroidetes Order II. Incertae sedis	2.48
Thermobispora bispora	Actinobacteria incertae sedis	2.15
Symbiobacterium thermophilum	Clostridiales	1.50
Sphaerobacter thermophilus	Sphaerobacterales	1.08
Thermobifida fusca	Streptosporangiales	0.75

Table S11. Comparative analysis of single-copy marker genes sequence of abundant species present in ZC4 compost microbial communities

Marker gene	*T. bispora*	*S. thermophilus*	*R. marinus*	*T. fusca*	*S. thermophilum*
50S ribosomal protein L6	100	99	99	100	90
50S ribosomal protein L21	100	99	100	99	99
DNA-directed RNA polymerase subunit beta	100	99	98	100	100
Ribosomal RNA small subunit methyltransferase E	99	99	95	100	Missing in the reference
DNA primase	100	99	94	100	86
DNA-directed RNA polymerase subunit alpha	92(1)	100	98	100	100(3)
DNA translocase SpoIIIE [cell division protein FtsK/SpoIIIE]	99	99	95	100(2)	96

(1) Average coverage is 80%; (2) Alignment coverage is 97%; (3) Alignment coverage is 92%.

Table S12. Comparative analysis of *Rhodothermus marinus* single-copy marker genes sequence present in ZC4 compost microbial communities

Marker gene	*R. marinus* SG0.5JP17-171	*R. marinus* SG0.5JP17-172	*R. marinus* DSM 4252
50S ribosomal protein L6	99	99	98
50S ribosomal protein L21	100	100	95
DNA-directed RNA polymerase subunit beta	100	100	98
Ribosomal RNA small subunit methyltransferase E	100	100	95
DNA primase	100	100	94
DNA-directed RNA polymerase subunit alpha	99	99	98
DNA translocase SpoIIIE [cell division protein FtsK/SpoIIIE]	97	97	95

Numbers are Blast identity values (%) in amino acids. Average alignment coverage is 98%.

Table S13. Most abundant bacterial species in ZC4 metatranscriptome. Classifications were done by program myTaxa on CDSs identified by the IMG pipeline. The numbers in each day column refer to relative abundance expressed in per thousand (only the top 30 are shown). The last column shows the absolute number of CDSs for each species across all samples

Species	D01	D03	D07	D15	D30	D64	D78	D99	Total for each species
Symbiobacterium thermophilum	34.95	71.00	121.06	61.24	14.09	60.80	21.11	2.50	15847
Rhodothermus marinus	2.47	4.92	25.12	89.06	86.42	12.70	92.76	5.94	10026
Thermobacillus composti	55.14	27.01	18.84	43.29	63.02	16.61	23.43	1.48	9490
Thermaerobacter marianensis	8.61	12.89	80.70	77.05	21.73	14.47	29.94	5.17	9082
Thermobispora bispora	42.26	40.66	62.69	18.08	26.14	12.94	17.39	6.66	9009
Streptosporangium roseum	28.86	28.70	37.25	12.08	19.14	9.37	8.88	7.68	6037
Paenibacillus mucilaginosus	56.62	15.43	16.61	17.76	14.49	13.86	11.08	1.31	5955
Solibacillus silvestris	33.63	27.03	4.44	1.28	0.69	50.86	0.68	2.25	5620
Brevibacillus brevis	21.49	50.10	15.89	17.83	6.71	12.31	4.78	0.68	5311
Pseudomonas mendocina	5.17	1.00	0.56	0.26	0.93	29.21	0.42	145.27	5302
Thermosediminibacter oceani	10.70	14.08	36.99	32.46	2.91	18.88	4.99	0.42	4880
Lysinibacillus sphaericus	28.12	23.56	4.34	1.31	0.71	42.55	0.89	1.36	4775
Sorangium cellulosum	1.15	14.29	13.55	31.53	51.63	2.31	15.97	0.81	4619
Klebsiella pneumoniae	2.97	0.10	0.05	0.00	0.03	0.31	0.05	182.68	4463
Sphaerobacter thermophilus	2.74	12.58	15.99	4.89	14.38	4.35	24.53	31.05	3463
Thermobifida fusca	2.70	2.76	4.51	0.70	61.46	3.44	17.12	5.17	3405
Bacillus coagulans	22.19	15.43	11.04	10.51	2.48	14.45	2.94	0.59	3361
Pseudomonas putida	12.23	1.28	0.42	0.38	0.95	3.23	0.53	91.28	2995
Kyrpidia tusciae	9.24	11.94	13.64	15.08	7.80	8.15	7.20	0.81	2850
Geobacillus thermodenitrificans	16.34	18.55	7.74	6.17	2.43	11.32	2.00	0.42	2766
Geobacillus sp. WCH70	17.35	13.77	10.48	7.67	2.48	8.15	1.89	0.93	2621
Paenibacillus sp. JDR-2	19.17	5.87	4.41	4.82	8.72	12.60	7.25	2.12	2619
Mahella australiensis	2.45	9.03	15.75	17.70	4.94	8.90	9.35	0.76	2570
Gemmatimonas aurantiaca	2.16	7.32	10.48	7.76	16.94	3.74	25.53	0.68	2437
Clostridium thermocellum	2.47	11.06	10.80	9.74	3.70	13.65	6.36	0.34	2329
Geobacillus sp. Y4.1MC1	17.83	10.30	9.43	7.19	1.72	5.50	1.84	0.34	2253
Clostridium acidurici	1.96	13.39	5.95	6.87	0.58	18.42	2.00	0.13	2149
Anoxybacillus flavithermus	11.33	10.72	7.81	6.96	1.88	7.56	2.10	0.21	2021
Thermomonospora curvata	5.08	5.99	8.76	2.14	11.34	4.54	7.04	12.30	2013
Bacillus megaterium	7.64	6.90	5.11	5.33	2.33	7.37	1.89	19.09	1977

Table S14. Five most abundant OTUs based on 16S data for ZC4 (all samples). Each OTU is identified by the number assigned to it by the clustering procedure used

Rank	OTU	Relative abundance (%)	Best BLAST hit	GenBank accession number of best hit
1	537822506	4.47	*Calditerricola yamamurae*	AB308475.1
2	576372771	2.89	Uncultured bacterium	KF911139.1
3	574661859	2.74	Uncultured bacterium	FN667161.1
4	723870961	2.48	Uncultured bacterium	KF911257.1
5	6102000	1.68	Uncultured bacterium	HE804952.1

BLAST was done against GenBank (NT) in November 2015. All hits reported had 100% identity and essentially 100% coverage against the 16S fragment that represents the OTU (lengths vary between 404 and 428 bp).

Table S15. Reads coverage for OTU537822506 draft genome

Contig ID	Average fold coverage	Contig length	% of coverage	Covered bases	Number of mapped reads (plus strand)	Number of mapped reads (minus strand)
NODE_2_length_98353_cov_21.3799_ID_25734092	64.80	98353	100	98353	39592	39535
NODE_8_length_54795_cov_29.4812_ID_25734106	126.44	54795	100	54795	49094	49281
NODE_15_length_46460_cov_23.0945_ID_25734120	77.11	46460	100	46460	23972	23884
NODE_28_length_36550_cov_24.6215_ID_25734146	75.66	36550	100	36550	18670	18643
NODE_44_length_31328_cov_23.7369_ID_25734178	110.61	31328	100	31328	24992	24780
NODE_55_length_28486_cov_21.7686_ID_25734200	66.27	28486	100	28486	11694	11676
NODE_73_length_26062_cov_23.7591_ID_25734240	75.02	26062	100	26062	13111	13093
NODE_96_length_23202_cov_27.5393_ID_25734290	102.52	23202	100	23202	17294	17258
NODE_105_length_22820_cov_20.3008_ID_25734310	73.06	22820	100	22820	10492	10515
NODE_112_length_22509_cov_22.3228_ID_25734322	72.50	22509	100	22509	12877	12831
NODE_120_length_21909_cov_24.3565_ID_25734338	94.23	21909	100	21909	15625	15563
NODE_132_length_20981_cov_21.3498_ID_25734370	65.25	20981	100	20981	7199	7209
NODE_138_length_20607_cov_23.3728_ID_25734386	70.60	20607	100	20607	9868	9842
NODE_143_length_20367_cov_23.1876_ID_25734396	64.91	20367	100	20367	8031	8021
NODE_150_length_20040_cov_22.9738_ID_25734410	73.37	20040	100	20040	10497	10476
NODE_171_length_18754_cov_24.1577_ID_25734456	84.39	18754	100	18754	11182	11145
NODE_186_length_18106_cov_21.423_ID_25734490	67.25	18106	100	18106	8165	8155
NODE_188_length_18056_cov_24.7152_ID_25734494	80.62	18056	100	18056	10115	10119
NODE_190_length_17982_cov_24.4363_ID_25734498	78.88	17982	100	17982	9869	9876
NODE_203_length_17546_cov_23.9182_ID_25734522	66.91	17546	100	17546	6595	6549
NODE_205_length_17527_cov_22.775_ID_25734530	70.42	17527	100	17527	7073	7090
NODE_209_length_17442_cov_23.2214_ID_25734538	67.39	17442	100	17442	7626	7617
NODE_227_length_16684_cov_21.1185_ID_25734576	63.51	16684	100	16684	6600	6576
NODE_237_length_16441_cov_22.4961_ID_25734600	90.24	16441	100	16441	10876	10688
NODE_247_length_16250_cov_22.6477_ID_25734620	79.63	16250	100	16250	8465	8477
NODE_269_length_15681_cov_19.2403_ID_25734668	65.06	15681	100	15681	5688	5694
NODE_310_length_14934_cov_25.981_ID_25734750	70.07	14934	100	14934	6245	6246
NODE_315_length_14835_cov_19.808_ID_25734760	63.91	14835	100	14835	6525	6514
NODE_330_length_14550_cov_22.8088_ID_25734796	65.68	14550	100	14550	6153	6153
NODE_350_length_14286_cov_20.5672_ID_25734840	69.86	14286	100	14286	6552	6527
NODE_353_length_14209_cov_20.2855_ID_25734846	70.43	14209	100	14209	7492	7472
NODE_370_length_14038_cov_23.4202_ID_25734880	78.13	14038	100	14038	7991	7987

NODE_394_length_13703_cov_23.992_ID_25734926	74.42	13703	100	13703	7183	7197
NODE_398_length_13631_cov_17.1474_ID_25734934	62.54	13631	100	13631	4838	4840
NODE_409_length_13517_cov_23.5516_ID_25734958	79.57	13517	100	13517	6445	6403
NODE_440_length_13049_cov_25.9056_ID_25735018	61.92	13049	100	13049	4492	4502
NODE_447_length_12925_cov_22.1227_ID_25735032	65.56	12925	100	12925	5160	5166
NODE_450_length_12915_cov_22.2675_ID_25735038	77.02	12915	99.9923	12914	7361	7230
NODE_474_length_12651_cov_21.638_ID_25735086	73.35	12651	100	12651	6164	6172
NODE_489_length_12374_cov_23.5117_ID_25735124	65.63	12374	100	12374	4609	4634
NODE_515_length_12069_cov_22.1046_ID_25735190	60.48	12069	100	12069	3857	3853
NODE_531_length_11810_cov_15.7664_ID_25735224	41.54	11810	100	11810	3269	3276
NODE_540_length_11697_cov_24.9477_ID_25735244	66.14	11697	100	11697	4444	4426
NODE_546_length_11556_cov_19.2857_ID_25735256	73.07	11556	100	11556	5564	5576
NODE_550_length_11546_cov_20.6113_ID_25735264	98.59	11546	100	11546	9646	9618
NODE_551_length_11545_cov_26.3196_ID_25735146	81.86	11545	100	11545	7994	8024
NODE_564_length_11454_cov_24.6258_ID_25735290	127.47	11454	100	11454	11829	11895
NODE_572_length_11374_cov_24.5465_ID_25735306	68.76	11374	100	11374	5085	5082
NODE_583_length_11236_cov_24.2932_ID_25735332	69.95	11236	100	11236	5587	5583
NODE_592_length_11170_cov_26.4034_ID_25735350	76.65	11170	100	11170	4837	4803
NODE_599_length_11103_cov_19.2783_ID_25735364	73.39	11103	100	11103	5320	5330
NODE_631_length_10858_cov_22.979_ID_25735426	66.90	10858	100	10858	3879	3874
NODE_644_length_10779_cov_20.1708_ID_25735458	67.41	10779	100	10779	4675	4673
NODE_647_length_10752_cov_25.0751_ID_25735464	74.92	10752	100	10752	5774	5740
NODE_651_length_10709_cov_23.3723_ID_25735476	73.69	10709	100	10709	5545	5502
NODE_653_length_10686_cov_18.3801_ID_25735480	66.05	10686	100	10686	4527	4504
NODE_683_length_10450_cov_17.1264_ID_25735540	87.46	10450	100	10450	6014	5955
NODE_686_length_10434_cov_22.8595_ID_25735550	62.19	10434	100	10434	3105	3101
NODE_710_length_10211_cov_17.2597_ID_25735598	74.71	10211	100	10211	6141	6139
NODE_711_length_10202_cov_23.9792_ID_25735600	75.42	10202	100	10202	4751	4830
NODE_717_length_10164_cov_22.8456_ID_25735612	70.41	10164	100	10164	4431	4427
NODE_719_length_10131_cov_22.1551_ID_25735616	64.24	10131	100	10131	4694	4684
NODE_720_length_10115_cov_20.5188_ID_25735618	69.51	10115	100	10115	5032	5015
NODE_721_length_10083_cov_23.0006_ID_25735620	65.71	10083	100	10083	4040	4015
NODE_729_length_10044_cov_23.7608_ID_25735636	69.85	10044	100	10044	4146	4148
NODE_738_length_9984_cov_23.5568_ID_25735654	90.51	9984	100	9984	5311	5302
NODE_770_length_9765_cov_9.21687_ID_25735722	37.18	9765	100	9765	2851	2843
NODE_783_length_9648_cov_24.3087_ID_25735754	74.55	9648	100	9648	4466	4484

NODE_807_length_9515_cov_17.144_ID_25735802	82.47	9515	100	9515	5452	5456
NODE_834_length_9353_cov_24.7799_ID_25735854	76.04	9353	100	9353	5042	5043
NODE_839_length_9329_cov_26.0514_ID_25735868	85.53	9329	100	9329	6410	6370
NODE_873_length_9104_cov_24.3215_ID_25735938	87.27	9104	100	9104	6653	6657
NODE_899_length_9029_cov_18.9487_ID_25735990	64.59	9029	100	9029	3645	3634
NODE_953_length_8773_cov_24.7336_ID_25736118	89.44	8773	100	8773	4181	4162
NODE_969_length_8694_cov_26.363_ID_25736150	98.86	8694	100	8694	6673	6682
NODE_970_length_8694_cov_25.1711_ID_25736152	68.42	8694	100	8694	2644	2666
NODE_1024_length_8464_cov_23.1316_ID_25736278	67.37	8464	100	8464	3381	3375
NODE_1053_length_8314_cov_24.2392_ID_25736348	85.98	8314	100	8314	4856	4861
NODE_1055_length_8308_cov_11.3963_ID_25736352	50.55	8308	100	8308	3672	3671
NODE_1067_length_8255_cov_16.2841_ID_25736376	76.43	8255	100	8255	4892	4925
NODE_1073_length_8204_cov_22.7847_ID_25736388	61.97	8204	100	8204	2443	2453
NODE_1109_length_8077_cov_20.2742_ID_25736468	73.52	8077	100	8077	3815	3802
NODE_1111_length_8074_cov_6.22508_ID_25736472	15.99	8074	100	8074	1058	1064
NODE_1181_length_7791_cov_16.7524_ID_25736608	62.00	7791	100	7791	4345	4329
NODE_1183_length_7790_cov_8.42072_ID_25736612	47.73	7790	100	7790	2929	2921
NODE_1195_length_7747_cov_25.8411_ID_25736640	76.92	7747	100	7747	3624	3549
NODE_1197_length_7729_cov_14.8036_ID_25736644	66.59	7729	100	7729	3509	3512
NODE_1208_length_7707_cov_24.4157_ID_25736666	49.88	7707	100	7707	2181	2182
NODE_1311_length_7393_cov_17.2116_ID_25736872	78.85	7393	100	7393	3629	3634
NODE_1352_length_7290_cov_25.6395_ID_25736960	65.32	7290	100	7290	3207	3204
NODE_1366_length_7241_cov_21.7698_ID_25736988	63.25	7241	100	7241	2633	2631
NODE_1381_length_7203_cov_19.9684_ID_25737016	69.70	7203	100	7203	3085	3095
NODE_1416_length_7109_cov_26.1288_ID_25737082	73.84	7109	100	7109	3206	3200
NODE_1463_length_6975_cov_5.37721_ID_25737184	17.78	6975	99.6272	6949	1012	1024
NODE_1485_length_6924_cov_24.4159_ID_25737230	141.07	6924	100	6924	5251	5258
NODE_1486_length_6921_cov_18.4011_ID_25737232	57.92	6921	100	6921	2582	2567
NODE_1498_length_6885_cov_19.8418_ID_25737256	118.42	6885	100	6885	6786	6717
NODE_1579_length_6702_cov_15.5319_ID_25737422	59.38	6702	100	6702	2583	2587
NODE_1598_length_6650_cov_21.3525_ID_25737458	73.52	6650	100	6650	3019	3010
NODE_1630_length_6591_cov_19.6709_ID_25737534	69.21	6591	100	6591	2926	2914
NODE_1641_length_6577_cov_21.9548_ID_25737560	79.72	6577	100	6577	3619	3627
NODE_1699_length_6472_cov_22.3179_ID_25737676	68.90	6472	100	6472	2791	2786
NODE_1701_length_6472_cov_22.759_ID_25737680	73.11	6472	100	6472	3454	3451
NODE_1744_length_6405_cov_21.8499_ID_25737768	64.17	6405	100	6405	2182	2187

NODE_1768_length_6362_cov_24.6395_ID_25737820	66.55	6362	100	6362	2691	2675
NODE_1887_length_6153_cov_21.3999_ID_25738076	69.96	6153	100	6153	3388	3396
NODE_1921_length_6082_cov_22.3737_ID_25738150	68.82	6082	100	6082	2186	2178
NODE_1959_length_6015_cov_22.1536_ID_25738228	74.31	6015	100	6015	3045	3042
NODE_1984_length_5980_cov_27.9995_ID_25738282	85.68	5980	100	5980	3176	3169
NODE_2056_length_5863_cov_23.0793_ID_25738430	86.29	5863	100	5863	3438	3503
NODE_2242_length_5619_cov_20.5747_ID_25738806	69.13	5619	100	5619	2044	2049
NODE_2251_length_5611_cov_17.8319_ID_25738824	61.33	5611	100	5611	1944	1964
NODE_2311_length_5523_cov_30.374_ID_25738954	101.28	5523	100	5523	3654	3601
NODE_2334_length_5489_cov_22.8592_ID_25739004	66.64	5489	100	5489	2108	2120
NODE_2336_length_5489_cov_23.0281_ID_25739008	58.53	5489	100	5489	1524	1518
NODE_2337_length_5487_cov_15.4007_ID_25739010	76.73	5487	100	5487	3124	3119
NODE_2440_length_5366_cov_22.605_ID_25739226	66.89	5366	100	5366	2168	2171
NODE_2459_length_5341_cov_19.5294_ID_25739264	76.26	5341	100	5341	3326	3342
NODE_2464_length_5336_cov_23.9973_ID_25739278	75.17	5336	100	5336	2431	2422
NODE_2487_length_5311_cov_23.5737_ID_25739328	82.64	5311	100	5311	3184	3200
NODE_2510_length_5286_cov_27.2083_ID_25739376	65.17	5286	100	5286	1906	1894
NODE_2526_length_5272_cov_4.74572_ID_25739406	18.83	5272	100	5272	1604	1605
NODE_2529_length_5267_cov_22.1116_ID_25739412	66.23	5267	100	5267	2410	2408
NODE_2594_length_5210_cov_23.0586_ID_25739554	79.87	5210	100	5210	2933	2905
NODE_2672_length_5128_cov_14.3918_ID_25739718	61.13	5128	100	5128	1765	1753
NODE_2715_length_5085_cov_7.48103_ID_25739800	22.41	5085	100	5085	857	847
NODE_2732_length_5071_cov_20.3861_ID_25739838	75.43	5071	100	5071	2608	2601
NODE_2829_length_4978_cov_22.7411_ID_25740054	76.45	4978	100	4978	2360	2362
NODE_2849_length_4962_cov_19.497_ID_25740094	66.03	4962	100	4962	2089	2099
NODE_2852_length_4961_cov_23.3847_ID_25740100	65.33	4961	100	4961	2194	2195
NODE_2980_length_4836_cov_22.3713_ID_25740354	94.78	4836	100	4836	2879	2862
NODE_3001_length_4825_cov_19.3229_ID_25740404	82.00	4825	100	4825	3029	3032
NODE_3007_length_4818_cov_25.096_ID_25740416	73.24	4818	100	4818	2669	2669
NODE_3020_length_4809_cov_6.95985_ID_25740444	35.67	4809	100	4809	1632	1629
NODE_3091_length_4753_cov_23.1153_ID_25740588	206.81	4753	100	4753	8196	7862
NODE_3183_length_4676_cov_15.9354_ID_25740784	54.21	4676	100	4676	1432	1401
NODE_3238_length_4631_cov_26.3362_ID_25740902	141.21	4631	100	4631	3597	3578
NODE_3268_length_4610_cov_18.944_ID_25740960	58.99	4610	100	4610	1647	1643
NODE_3279_length_4604_cov_24.1237_ID_25740982	65.36	4604	100	4604	1956	1979
NODE_3285_length_4598_cov_22.672_ID_25741000	59.76	4598	100	4598	1636	1640

NODE_3414_length_4509_cov_22.1153_ID_25741260	76.86	4509	100	4509	2557	2566
NODE_3462_length_4477_cov_24.7052_ID_25741352	63.85	4477	100	4477	1387	1397
NODE_3522_length_4440_cov_21.022_ID_25741478	54.08	4440	100	4440	1266	1264
NODE_3594_length_4390_cov_20.416_ID_25741636	60.75	4390	100	4390	1449	1445
NODE_3686_length_4340_cov_17.9289_ID_25741824	66.60	4340	100	4340	1852	1861
NODE_3713_length_4329_cov_14.2857_ID_25741880	66.44	4329	100	4329	1973	1967
NODE_3829_length_4265_cov_30.7555_ID_25742120	104.00	4265	100	4265	3533	3553
NODE_3834_length_4259_cov_18.7281_ID_25742130	64.41	4259	100	4259	1485	1483
NODE_3951_length_4201_cov_21.25_ID_25742368	65.38	4201	100	4201	1735	1730
NODE_4073_length_4143_cov_16.4144_ID_25742630	85.80	4143	100	4143	2464	2477
NODE_4082_length_4137_cov_18.9264_ID_25742648	109.29	4137	100	4137	3173	3235
NODE_4175_length_4088_cov_14.9614_ID_25742842	37.25	4088	100	4088	1146	1152
NODE_4192_length_4080_cov_24.5456_ID_25742876	69.24	4080	100	4080	1802	1808
NODE_4206_length_4071_cov_24.3373_ID_25742904	115.50	4071	100	4071	2146	2145
NODE_4369_length_3976_cov_21.2711_ID_25743252	69.76	3976	100	3976	1952	1952
NODE_4398_length_3963_cov_25.6688_ID_25743310	104.89	3963	100	3963	3571	3573
NODE_4445_length_3943_cov_14.8655_ID_25743400	54.29	3943	100	3943	1131	1131
NODE_4706_length_3827_cov_18.8971_ID_25743948	107.16	3827	100	3827	3189	3119
NODE_4790_length_3793_cov_21.253_ID_25744128	58.43	3793	100	3793	1031	1047
NODE_4801_length_3788_cov_14.8987_ID_25744148	46.95	3788	100	3788	1035	1034
NODE_4896_length_3753_cov_24.4162_ID_25744336	74.65	3753	100	3753	1620	1631
NODE_4904_length_3750_cov_23.8903_ID_25744352	61.64	3750	100	3750	1242	1253
NODE_5138_length_3674_cov_6.23214_ID_25744844	28.39	3674	100	3674	1152	1150
NODE_5211_length_3649_cov_11.8488_ID_25744994	54.26	3649	100	3649	1052	1063
NODE_5296_length_3623_cov_12.3765_ID_25745186	58.83	3623	100	3623	1143	1147
NODE_5390_length_3588_cov_18.7149_ID_25745374	57.84	3588	100	3588	1424	1427
NODE_5534_length_3544_cov_24.895_ID_25745676	72.34	3544	100	3544	1674	1654
NODE_5541_length_3541_cov_7.77829_ID_25745694	39.73	3541	100	3541	1258	1257
NODE_5829_length_3454_cov_17.7684_ID_25746304	86.66	3454	100	3454	2335	2324
NODE_6124_length_3370_cov_21.1142_ID_25746916	82.90	3370	100	3370	1858	1867
NODE_6277_length_3328_cov_10.6739_ID_25747240	57.01	3328	100	3328	1191	1158
NODE_6288_length_3325_cov_17.3904_ID_25747262	64.00	3325	100	3325	1250	1250
NODE_6295_length_3323_cov_20.2683_ID_25747276	66.89	3323	100	3323	1519	1513
NODE_6397_length_3292_cov_2.51695_ID_25747494	28.92	3292	100	3292	1395	1361
NODE_6493_length_3268_cov_13.4522_ID_25747682	42.35	3268	100	3268	750	747
NODE_6501_length_3266_cov_20.3051_ID_25747702	66.63	3266	100	3266	1194	1192

NODE_6524_length_3262_cov_19.6515_ID_25747748	64.27	3262	100	3262	1123	1130
NODE_6702_length_3214_cov_19.5174_ID_25748126	63.82	3214	100	3214	1083	1082
NODE_6730_length_3206_cov_2.77629_ID_25748180	15.30	3206	100	3206	703	700
NODE_6753_length_3202_cov_27.1027_ID_25748230	93.84	3202	100	3202	2074	2116
NODE_6771_length_3196_cov_14.1372_ID_25748266	63.04	3196	100	3196	993	995
NODE_6865_length_3170_cov_16.7957_ID_25748452	58.41	3170	100	3170	1157	1153
NODE_6990_length_3138_cov_13.6779_ID_25748696	61.55	3138	100	3138	1224	1215
NODE_7020_length_3132_cov_23.4527_ID_25748756	63.49	3132	100	3132	859	876
NODE_7089_length_3117_cov_12.1783_ID_25748898	63.14	3117	100	3117	1203	1201
NODE_7204_length_3093_cov_18.7974_ID_25749134	55.03	3093	100	3093	967	961
NODE_7379_length_3055_cov_8.46441_ID_25749512	40.43	3055	100	3055	875	870
NODE_7582_length_3015_cov_13.2488_ID_25749914	50.81	3015	100	3015	1222	1221
NODE_7621_length_3008_cov_22.7741_ID_25750000	73.76	3008	100	3008	1348	1353
NODE_7647_length_3004_cov_138.396_ID_25750056	269.71	3004	100	3004	2993	2992
NODE_7784_length_2977_cov_12.5007_ID_25750346	65.54	2977	100	2977	1532	1534
NODE_8024_length_2934_cov_8.87154_ID_25750842	40.13	2934	100	2934	837	837
NODE_8105_length_2920_cov_17.9388_ID_25751002	57.50	2920	100	2920	920	907
NODE_8711_length_2812_cov_15.2622_ID_25746098	63.64	2812	100	2812	1289	1306
NODE_8940_length_2780_cov_4.99334_ID_25752716	15.51	2780	100	2780	512	507
NODE_8953_length_2778_cov_6.0074_ID_25752742	37.16	2778	100	2778	805	807
NODE_9083_length_2756_cov_12.1086_ID_25753014	55.20	2756	100	2756	897	900
NODE_9525_length_2696_cov_21.21_ID_25753908	60.60	2696	100	2696	967	962
NODE_9617_length_2683_cov_21.718_ID_25754094	58.88	2683	100	2683	808	811
NODE_9831_length_2652_cov_17.0629_ID_25754544	58.94	2652	100	2652	979	981
NODE_10581_length_2564_cov_16.4435_ID_25756102	60.12	2564	100	2564	693	690
NODE_10835_length_2535_cov_15.4028_ID_25756626	41.32	2535	100	2535	670	666
NODE_11095_length_2508_cov_6.40683_ID_25757148	47.10	2508	100	2508	907	903
NODE_11381_length_2476_cov_18.476_ID_25757752	103.07	2476	100	2476	1943	1907
NODE_11512_length_2463_cov_23.3382_ID_25758032	71.43	2463	100	2463	1094	1067
NODE_11516_length_2463_cov_7.63956_ID_25758040	21.99	2463	100	2463	436	442
NODE_11636_length_2451_cov_23.9646_ID_25758300	69.95	2451	100	2451	917	913
NODE_11894_length_2424_cov_23.9182_ID_25758834	66.10	2424	100	2424	859	850
NODE_13150_length_2307_cov_12.9556_ID_25761400	68.94	2307	100	2307	1353	1350
NODE_13460_length_2280_cov_17.8212_ID_25762030	65.71	2280	100	2280	883	883
NODE_13495_length_2278_cov_8.67106_ID_25762098	64.63	2278	100	2278	901	892
NODE_13622_length_2267_cov_2.55936_ID_25762360	26.50	2267	100	2267	938	931

NODE_14126_length_2226_cov_3.8725_ID_25763418	14.56	2226	100	2226	484	487
NODE_14280_length_2215_cov_9.86389_ID_25763730	63.16	2215	100	2215	897	895
NODE_14525_length_2196_cov_3.73431_ID_25764234	13.57	2196	99.9089	2194	425	427
NODE_14836_length_2174_cov_20.9957_ID_25764896	63.07	2174	100	2174	699	702
NODE_15131_length_2154_cov_7.67549_ID_25765504	34.88	2154	99.4893	2143	702	704
NODE_15162_length_2152_cov_6.38217_ID_25765564	25.71	2152	100	2152	754	760
NODE_15213_length_2149_cov_14.237_ID_25765670	59.97	2149	100	2149	546	555
NODE_15237_length_2148_cov_125.166_ID_25765718	408.67	2148	100	2148	3417	3421
NODE_16170_length_2087_cov_20.29_ID_25767620	71.76	2087	100	2087	1209	1205
NODE_16489_length_2069_cov_7.78815_ID_25768274	53.14	2069	100	2069	1218	1222
NODE_16672_length_2060_cov_8.75643_ID_25768648	104.37	2060	100	2060	1811	1772
NODE_16916_length_2046_cov_37.0813_ID_25769146	79.80	2046	100	2046	815	815
NODE_17086_length_2037_cov_2.88367_ID_25769500	39.51	2037	100	2037	1432	1434
NODE_17630_length_2007_cov_19.8865_ID_25770592	61.73	2007	100	2007	761	773
NODE_18003_length_1986_cov_8.19487_ID_25771362	25.94	1986	100	1986	424	423
NODE_18101_length_1981_cov_3.33981_ID_25771560	363.69	1981	100	1981	14389	384
NODE_18516_length_1960_cov_6.63356_ID_25772406	17.47	1960	100	1960	305	303
NODE_18688_length_1951_cov_6.63234_ID_25772754	24.89	1951	100	1951	483	475
NODE_19240_length_1925_cov_35.9318_ID_25773872	298.35	1925	100	1925	3613	3615
NODE_19312_length_1921_cov_14.3156_ID_25774020	63.48	1921	100	1921	596	596
NODE_19533_length_1912_cov_9.49101_ID_25774476	43.75	1912	100	1912	545	554
NODE_19828_length_1899_cov_3.5472_ID_25775078	24.39	1899	100	1899	496	494
NODE_19841_length_1899_cov_14.7552_ID_25775104	74.08	1899	100	1899	1019	1004
NODE_20342_length_1875_cov_18.5095_ID_25749228	54.88	1875	100	1875	424	432
NODE_20780_length_1857_cov_12.6264_ID_25777014	63.36	1857	100	1857	601	608
NODE_20949_length_1850_cov_8.43937_ID_25777358	55.08	1850	100	1850	691	687
NODE_21018_length_1847_cov_7.7565_ID_25777512	36.09	1847	100	1847	738	727
NODE_21152_length_1841_cov_3.01247_ID_25777784	15.27	1841	100	1841	467	463
NODE_21564_length_1824_cov_3.0332_ID_25778624	12.46	1824	100	1824	317	317
NODE_21655_length_1821_cov_31.7076_ID_25778804	103.80	1821	100	1821	924	921
NODE_21662_length_1821_cov_13.129_ID_25778818	51.48	1821	100	1821	653	665
NODE_21817_length_1814_cov_6.91595_ID_25779146	27.58	1814	100	1814	434	443
NODE_22014_length_1807_cov_2.56705_ID_25779552	23.60	1807	100	1807	751	745
NODE_22244_length_1798_cov_3.99128_ID_25780014	11.90	1798	100	1798	281	282
NODE_22934_length_1771_cov_9.92798_ID_25781424	32.23	1771	100	1771	477	484
NODE_23419_length_1753_cov_9.82518_ID_25760226	62.75	1753	100	1753	582	593

NODE_23442_length_1753_cov_32.0453_ID_25782486	102.85	1753	100	1753	793	797
NODE_24482_length_1717_cov_5.61951_ID_25784588	36.49	1717	100	1717	651	643
NODE_24533_length_1716_cov_9.01586_ID_25784688	57.97	1716	100	1716	459	453
NODE_24762_length_1708_cov_5.32128_ID_25785148	26.96	1708	100	1708	352	352
NODE_25104_length_1698_cov_3.17212_ID_25785842	18.89	1698	100	1698	455	459
NODE_25263_length_1693_cov_3.89851_ID_25786166	34.23	1693	100	1693	551	552
NODE_25651_length_1681_cov_18.4738_ID_25786968	69.32	1681	100	1681	741	725
NODE_26008_length_1670_cov_21.4721_ID_25787696	64.44	1670	100	1670	502	498
NODE_26671_length_1650_cov_10.4056_ID_25789070	33.63	1650	100	1650	433	442
NODE_26855_length_1644_cov_5.92789_ID_25789450	62.48	1644	100	1644	1113	1106
NODE_27414_length_1629_cov_4.4201_ID_25790568	70.97	1629	100	1629	1036	1054
NODE_27575_length_1624_cov_23.8177_ID_25790892	128.97	1624	100	1624	1389	1390
NODE_27664_length_1622_cov_171.588_ID_25791070	151.86	1622	99.4451	1613	861	859
NODE_28085_length_1609_cov_3.8094_ID_25791916	19.79	1609	100	1609	240	249
NODE_28248_length_1604_cov_2.07728_ID_25792252	45.36	1604	100	1604	1201	1210
NODE_28916_length_1586_cov_2.33731_ID_25793638	22.61	1586	100	1586	677	678
NODE_29076_length_1583_cov_42.156_ID_25793962	164.20	1583	100	1583	1160	1172
NODE_29556_length_1570_cov_5.41929_ID_25794934	17.95	1570	100	1570	292	295
NODE_29788_length_1564_cov_5.25286_ID_25795412	66.58	1564	100	1564	762	771
NODE_30569_length_1545_cov_3.49251_ID_25796986	22.99	1545	100	1545	452	455
NODE_30723_length_1541_cov_10.8852_ID_25797296	61.34	1541	100	1541	731	723
NODE_30987_length_1536_cov_1.83139_ID_25797834	17.74	1536	100	1536	395	395
NODE_31244_length_1530_cov_12.7103_ID_25798352	79.38	1530	100	1530	1144	1134
NODE_31478_length_1524_cov_2.53766_ID_25766872	9.34	1524	100	1524	214	211
NODE_31507_length_1524_cov_12.6828_ID_25798890	65.02	1524	100	1524	457	461
NODE_31916_length_1515_cov_9.10779_ID_25799714	66.45	1515	100	1515	679	684
NODE_32522_length_1501_cov_3.39045_ID_25800940	18.30	1501	100	1501	405	402
NODE_32908_length_1493_cov_21.238_ID_25801730	62.68	1493	100	1493	518	518
NODE_32966_length_1491_cov_6.07496_ID_25801858	56.24	1491	100	1491	387	390
NODE_33096_length_1488_cov_1.71935_ID_25802120	30.11	1488	100	1488	798	795
NODE_33896_length_1472_cov_2.8595_ID_25803744	9.87	1472	100	1472	135	133
NODE_34025_length_1469_cov_16.3017_ID_25804018	53.02	1469	100	1469	416	416
NODE_34198_length_1466_cov_15.4658_ID_25804368	78.22	1466	100	1466	1098	1095
NODE_34454_length_1461_cov_18.6749_ID_25804882	67.79	1461	100	1461	618	621
NODE_34665_length_1456_cov_5.98115_ID_25805318	36.30	1456	100	1456	496	499
NODE_34692_length_1456_cov_21.5504_ID_25805376	116.25	1456	100	1456	1356	1375

NODE_35803_length_1434_cov_2.54237_ID_25807650	21.18	1434	100	1434	505	502
NODE_35882_length_1433_cov_3.23746_ID_25807808	21.39	1433	100	1433	366	365
NODE_35973_length_1431_cov_2.06795_ID_25807994	57.84	1431	100	1431	1108	1098
NODE_36023_length_1430_cov_5.70584_ID_25808098	23.40	1430	100	1430	423	422
NODE_36527_length_1421_cov_7.76562_ID_25809102	15.61	1421	100	1421	145	145
NODE_36613_length_1419_cov_1.35469_ID_25809270	26.72	1419	100	1419	643	660
NODE_36718_length_1417_cov_2.39776_ID_25809488	13.62	1417	100	1417	304	305
NODE_36723_length_1417_cov_2.62687_ID_25809502	13.85	1417	100	1417	250	249
NODE_36840_length_1415_cov_2.07399_ID_25809748	20.37	1415	100	1415	526	523
NODE_37053_length_1411_cov_8.98651_ID_25810178	47.33	1411	100	1411	719	712
NODE_37894_length_1396_cov_4.60197_ID_25811874	23.04	1396	100	1396	549	549
NODE_38202_length_1391_cov_3.80289_ID_25812498	13.17	1391	100	1391	144	147
NODE_38698_length_1383_cov_1.79173_ID_25813506	18.61	1383	100	1383	515	517
NODE_38852_length_1380_cov_5.76132_ID_25813834	664.40	1380	100	1380	262	23456
NODE_39111_length_1376_cov_9.34488_ID_25814360	23.95	1376	100	1376	259	261
NODE_39277_length_1373_cov_2.65355_ID_25814692	12.57	1373	100	1373	277	277
NODE_39320_length_1373_cov_8.37037_ID_25814776	63.79	1373	100	1373	466	469
NODE_39533_length_1369_cov_6.65944_ID_25815202	39.35	1369	100	1369	398	392
NODE_39688_length_1366_cov_3.72149_ID_25815522	42.66	1366	100	1366	517	519
NODE_39869_length_1363_cov_2.01166_ID_25815892	11.95	1363	100	1363	264	264
NODE_40395_length_1354_cov_2.35161_ID_25816954	9.42	1354	100	1354	208	209
NODE_40425_length_1354_cov_2.19499_ID_25817018	29.03	1354	100	1354	640	655
NODE_40449_length_1354_cov_8.49413_ID_25817064	63.98	1354	100	1354	547	542
NODE_40689_length_1350_cov_5.53888_ID_25817544	24.97	1350	100	1350	258	262
NODE_42019_length_1330_cov_8.18915_ID_25820224	36.46	1330	100	1330	390	396
NODE_42090_length_1329_cov_2.71885_ID_25820370	14.71	1329	100	1329	302	301
NODE_42488_length_1323_cov_93.439_ID_25821178	199.96	1323	100	1323	1133	1147
NODE_42832_length_1317_cov_1.75242_ID_25821874	16.26	1317	100	1317	376	376
NODE_42912_length_1316_cov_3.46247_ID_25822034	52.29	1316	100	1316	603	590
NODE_42967_length_1315_cov_2.76898_ID_25822148	33.32	1315	100	1315	616	603
NODE_43134_length_1313_cov_11.2338_ID_25822492	59.30	1313	97.7913	1284	546	543
NODE_43245_length_1311_cov_2.66126_ID_25822714	19.66	1311	100	1311	493	494
NODE_43980_length_1301_cov_2.91912_ID_25824214	12.31	1301	100	1301	217	217
NODE_44218_length_1297_cov_4.08033_ID_25824708	36.74	1297	100	1297	594	579
NODE_44404_length_1295_cov_3.81691_ID_25825094	49.66	1295	100	1295	1317	1315
NODE_44655_length_1291_cov_5.16145_ID_25825606	27.68	1291	100	1291	321	335

NODE_44771_length_1290_cov_2.81781_ID_25825848	86.99	1290	100	1290	1242	1232
NODE_45651_length_1277_cov_3.05417_ID_25827600	18.16	1277	100	1277	379	379
NODE_45657_length_1277_cov_1.85333_ID_25827612	18.02	1277	100	1277	411	412
NODE_45970_length_1273_cov_2.72575_ID_25828242	59.05	1273	100	1273	1067	1068
NODE_46326_length_1269_cov_9.61158_ID_25828954	52.29	1269	100	1269	398	391
NODE_46386_length_1268_cov_17.2309_ID_25829074	70.29	1268	100	1268	418	432
NODE_46682_length_1264_cov_3.50126_ID_25829670	22.12	1264	96.9146	1225	394	395
NODE_47297_length_1256_cov_2.74131_ID_25830916	27.43	1256	100	1256	725	726
NODE_49227_length_1231_cov_3.22444_ID_25834834	115.68	1231	100	1231	1223	1244
NODE_49266_length_1231_cov_3.77296_ID_25834914	12.13	1231	100	1231	219	218
NODE_49306_length_1230_cov_2.37901_ID_25834994	15.21	1230	100	1230	327	328
NODE_49328_length_1230_cov_10.5768_ID_25835038	58.12	1230	100	1230	306	304
NODE_49413_length_1229_cov_6.62153_ID_25835214	39.73	1229	100	1229	299	294
NODE_49442_length_1229_cov_10.7101_ID_25835276	59.25	1229	100	1229	336	334
NODE_49511_length_1228_cov_3.53258_ID_25835418	26.77	1228	100	1228	443	443
NODE_49616_length_1226_cov_2.89817_ID_25835624	13.32	1226	100	1226	235	236
NODE_50419_length_1217_cov_1.84386_ID_25837262	55.83	1217	100	1217	804	805
NODE_50747_length_1214_cov_6.13105_ID_25837916	39.96	1214	100	1214	438	441
NODE_51227_length_1208_cov_1.54907_ID_25838898	8.54	1208	100	1208	165	163
NODE_51418_length_1206_cov_9.32684_ID_25839296	62.72	1206	100	1206	478	475
NODE_51739_length_1202_cov_2.03289_ID_25839950	57.10	1202	98.5857	1185	1612	1614
NODE_52189_length_1197_cov_4.8625_ID_25840872	24.37	1197	100	1197	436	435
NODE_52346_length_1195_cov_2.83453_ID_25841182	102.44	1195	100	1195	661	672
NODE_52582_length_1192_cov_2.05112_ID_25841646	13.46	1192	100	1192	249	248
NODE_52744_length_1190_cov_3.91824_ID_25841962	45.28	1190	100	1190	711	702
NODE_52783_length_1190_cov_3.13028_ID_25842044	30.23	1190	100	1190	692	692
NODE_53014_length_1188_cov_12.4167_ID_25842502	54.09	1188	100	1188	354	353
NODE_53048_length_1187_cov_2.32703_ID_25842570	21.16	1187	100	1187	507	509
NODE_53314_length_1184_cov_2.06775_ID_25843102	32.29	1184	100	1184	632	641
NODE_53603_length_1181_cov_2.80616_ID_25843674	17.65	1181	100	1181	413	412
NODE_53806_length_1179_cov_2.76316_ID_25844078	29.27	1179	99.8304	1177	499	493
NODE_54735_length_1169_cov_3.93681_ID_25845956	13.90	1169	100	1169	240	240
NODE_55176_length_1165_cov_2.31893_ID_25846852	64.66	1165	100	1165	1593	1591
NODE_55438_length_1163_cov_3.10313_ID_25847376	10.56	1163	100	1163	175	174
NODE_55834_length_1159_cov_14.1201_ID_25848174	52.13	1159	100	1159	400	403
NODE_56218_length_1155_cov_5.32931_ID_25848936	16.07	1155	100	1155	172	168

NODE_56598_length_1151_cov_2.20577_ID_25849700	19.34	1151	100	1151	437	433
NODE_56900_length_1148_cov_2.81232_ID_25850312	47.79	1148	100	1148	397	415
NODE_57116_length_1146_cov_3.87465_ID_25850752	14.41	1146	100	1146	259	260
NODE_57622_length_1141_cov_2.10056_ID_25851766	56.38	1141	100	1141	837	828
NODE_58240_length_1135_cov_1.88374_ID_25853002	21.23	1135	100	1135	205	208
NODE_58502_length_1133_cov_5.77178_ID_25853536	26.17	1133	100	1133	445	443
NODE_58647_length_1131_cov_2.01898_ID_25853832	9.67	1131	100	1131	193	195
NODE_58883_length_1129_cov_1.30798_ID_25854310	13.68	1129	100	1129	324	325
NODE_59049_length_1128_cov_2.18459_ID_25854646	10.65	1128	100	1128	209	210
NODE_59225_length_1127_cov_5.74857_ID_25854994	47.72	1127	100	1127	1099	259
NODE_59837_length_1121_cov_3.35345_ID_25856218	47.81	1121	100	1121	745	758
NODE_60274_length_1117_cov_2.04038_ID_25857108	6.81	1117	100	1117	113	112
NODE_60427_length_1116_cov_26.0135_ID_25857418	67.46	1116	100	1116	459	454
NODE_60471_length_1115_cov_1.78613_ID_25857512	16.87	1115	100	1115	353	353
NODE_60585_length_1114_cov_2.66152_ID_25857746	30.10	1114	100	1114	377	381
NODE_61064_length_1110_cov_2.92062_ID_25858720	25.17	1110	100	1110	570	569
NODE_61179_length_1109_cov_10.2258_ID_25858948	64.80	1109	100	1109	360	357
NODE_61852_length_1103_cov_1.77583_ID_25860308	21.94	1103	100	1103	514	513
NODE_61963_length_1102_cov_10.2302_ID_25860530	112.48	1102	100	1102	552	558
NODE_62261_length_1100_cov_5.43891_ID_25861134	23.52	1100	100	1100	200	199
NODE_62732_length_1096_cov_2.80765_ID_25862090	50.37	1096	100	1096	317	321
NODE_62878_length_1094_cov_9.05211_ID_25862394	42.77	1094	100	1094	242	244
NODE_62892_length_1094_cov_1.54376_ID_25862428	20.43	1094	100	1094	477	476
NODE_62997_length_1093_cov_3.89961_ID_25862638	46.44	1093	100	1093	575	573
NODE_63017_length_1093_cov_2.15157_ID_25862676	52.77	1093	100	1093	1225	1216
NODE_63327_length_1091_cov_5.09862_ID_25863294	15.62	1091	100	1091	190	191
NODE_63669_length_1088_cov_4.0821_ID_25863982	17.69	1088	100	1088	223	231
NODE_63769_length_1087_cov_4.7802_ID_25864182	15.25	1087	100	1087	202	203
NODE_64892_length_1078_cov_2.36464_ID_25866494	20.13	1078	100	1078	402	406
NODE_65033_length_1077_cov_3.164_ID_25866780	13.72	1077	100	1077	123	126
NODE_65119_length_1076_cov_2.37738_ID_25866962	22.81	1076	100	1076	272	275
NODE_65235_length_1075_cov_2.21643_ID_25867200	42.09	1075	100	1075	730	724
NODE_65239_length_1075_cov_3.46994_ID_25867208	13.07	1075	100	1075	201	200
NODE_65657_length_1072_cov_1.29045_ID_25868040	8.65	1072	100	1072	182	183
NODE_66415_length_1066_cov_1.44489_ID_25869576	16.41	1066	100	1066	224	240
NODE_66696_length_1064_cov_2.56332_ID_25870150	27.51	1064	100	1064	197	185

NODE_66942_length_1062_cov_1.98883_ID_25870650	21.21	1062	100	1062	476	472
NODE_67396_length_1059_cov_2.63136_ID_25871558	14.71	1059	100	1059	218	216
NODE_67472_length_1058_cov_2.31906_ID_25871714	19.98	1058	100	1058	364	362
NODE_67889_length_1055_cov_2.71677_ID_25872558	19.42	1055	100	1055	338	340
NODE_67983_length_1054_cov_4.63255_ID_25872742	12.48	1054	100	1054	150	150
NODE_68139_length_1053_cov_3.76844_ID_25873058	27.77	1053	100	1053	466	466
NODE_68145_length_1053_cov_3.61475_ID_25873070	26.48	1053	100	1053	491	491
NODE_68326_length_1052_cov_5.14974_ID_25873430	41.06	1052	100	1052	475	470
NODE_68520_length_1050_cov_1.97636_ID_25873830	14.65	1050	100	1050	274	275
NODE_68805_length_1048_cov_1.65808_ID_25874402	25.07	1048	100	1048	393	403
NODE_69196_length_1046_cov_6.42105_ID_25875196	37.41	1046	100	1046	332	319
NODE_69834_length_1041_cov_2.1639_ID_25876496	18.72	1041	100	1041	356	358
NODE_69951_length_1040_cov_2.72897_ID_25876730	14.05	1040	100	1040	270	269
NODE_70012_length_1040_cov_1.46521_ID_25876852	19.88	1040	100	1040	373	372
NODE_70242_length_1038_cov_2.6795_ID_25877324	22.72	1038	100	1038	362	370
NODE_71496_length_1030_cov_9.88877_ID_25879844	71.86	1030	100	1030	359	354
NODE_71555_length_1029_cov_2.1355_ID_25879962	8.73	1029	100	1029	156	158
NODE_71776_length_1028_cov_2.8633_ID_25880398	15.76	1028	100	1028	178	180
NODE_72729_length_1021_cov_1.85381_ID_25882322	15.60	1021	100	1021	319	319
NODE_72825_length_1021_cov_13.1377_ID_25882522	50.38	1021	100	1021	227	223
NODE_72843_length_1020_cov_1.48038_ID_25882560	16.77	1020	100	1020	382	382
NODE_73147_length_1019_cov_8.70488_ID_25883168	43.70	1019	100	1019	224	225
NODE_73664_length_1015_cov_3.12687_ID_25884232	45.97	1015	100	1015	624	608
NODE_73752_length_1014_cov_4.06403_ID_25884412	53.93	1014	100	1014	1127	1126
NODE_73772_length_1014_cov_4.65848_ID_25884452	22.99	1014	100	1014	305	303
NODE_73990_length_1013_cov_2.58761_ID_25884888	21.83	1013	100	1013	424	422
NODE_74104_length_1012_cov_1.26096_ID_25885122	9.84	1012	100	1012	201	201
NODE_74262_length_1011_cov_2.00535_ID_25885440	18.14	1011	100	1011	371	371
NODE_74430_length_1010_cov_3.30332_ID_25885780	30.62	1010	100	1010	504	506
NODE_74477_length_1010_cov_2.72669_ID_25885874	28.33	1010	100	1010	257	263
NODE_74580_length_1009_cov_2.62446_ID_25886076	23.11	1009	100	1009	445	447
NODE_75315_length_1005_cov_2.83621_ID_25887544	8.09	1005	100	1005	91	91
NODE_75541_length_1003_cov_11.9838_ID_25888002	81.00	1003	100	1003	697	690
NODE_75688_length_1003_cov_13.2106_ID_25888298	42.88	1003	99.8006	1001	242	245
NODE_75786_length_1002_cov_2.07676_ID_25888494	26.06	1002	100	1002	465	461
NODE_75919_length_1001_cov_1.97511_ID_25888764	39.22	1001	100	1001	535	525

NODE_75942_length_1001_cov_3.75433_ID_25813804	20.62	1001	100	1001	321	324
NODE_76394_length_998_cov_3.11292_ID_25889720	16.23	998	100	998	91	92
NODE_76526_length_997_cov_4.03804_ID_25889990	25.50	997	100	997	407	401
NODE_76537_length_997_cov_3.20326_ID_25890012	13.67	997	100	997	232	232
NODE_76922_length_995_cov_2.09368_ID_25890790	12.53	995	100	995	245	246
NODE_77231_length_993_cov_2.26638_ID_25891418	37.66	993	100	993	551	564
NODE_77373_length_992_cov_4.90273_ID_25891706	42.87	992	100	992	307	307
NODE_77787_length_990_cov_11.1895_ID_25892534	53.46	990	100	990	367	361
NODE_78241_length_987_cov_1.94176_ID_25893438	18.86	987	100	987	304	304
NODE_78700_length_984_cov_2.88203_ID_25894362	20.12	984	100	984	272	271
NODE_78703_length_984_cov_2.09592_ID_25894368	9.32	984	100	984	147	145
NODE_79059_length_982_cov_1.54807_ID_25895090	8.15	982	100	982	104	104
NODE_79066_length_982_cov_1.64309_ID_25895104	22.30	982	100	982	316	313
NODE_80009_length_976_cov_4.12903_ID_25896992	32.44	976	100	976	284	283
NODE_80086_length_976_cov_2.38042_ID_25897146	66.35	976	100	976	366	348
NODE_80604_length_973_cov_2.44866_ID_25898184	14.42	973	100	973	226	225
NODE_80639_length_972_cov_1.92179_ID_25898258	21.13	972	100	972	447	447
NODE_80661_length_972_cov_2.30838_ID_25898302	28.23	972	100	972	475	468
NODE_81216_length_969_cov_7.08632_ID_25899420	38.89	969	100	969	372	393
NODE_81264_length_969_cov_2.30269_ID_25899516	20.66	969	100	969	280	269
NODE_81363_length_968_cov_2.04826_ID_25899712	8.86	968	100	968	83	83
NODE_81573_length_967_cov_2.2764_ID_25900132	5.66	967	100	967	60	60
NODE_81746_length_966_cov_1.973_ID_25900484	13.50	966	100	966	233	232
NODE_81869_length_965_cov_4.89527_ID_25900732	31.05	965	100	965	209	212
NODE_82252_length_963_cov_2.68849_ID_25901498	120.32	963	100	963	949	915
NODE_82284_length_963_cov_2.2088_ID_25901562	21.71	963	100	963	406	404
NODE_82512_length_962_cov_2.75141_ID_25902016	18.13	962	100	962	226	228
NODE_82850_length_960_cov_4.82106_ID_25902696	32.47	960	100	960	207	208
NODE_83032_length_959_cov_4.13379_ID_25903060	44.75	959	100	959	697	692
NODE_83094_length_958_cov_2.60272_ID_25903184	22.52	958	100	958	353	354
NODE_83260_length_958_cov_7.23156_ID_25903516	34.18	958	100	958	271	269
NODE_83321_length_957_cov_2.41932_ID_25903638	48.47	957	100	957	929	923
NODE_83636_length_955_cov_2.9385_ID_25904270	41.73	955	100	955	752	752
NODE_83927_length_954_cov_2.40707_ID_25904860	35.85	954	100	954	639	637
NODE_84200_length_953_cov_3.63128_ID_25905410	24.98	953	100	953	250	250
NODE_84389_length_952_cov_2.056_ID_25905798	9.28	952	100	952	148	148

NODE_84438_length_951_cov_1.77574_ID_25905896	6.66	951	100	951	104	104
NODE_85042_length_948_cov_2.68197_ID_25907106	8.78	948	100	948	122	121
NODE_85054_length_948_cov_2.93456_ID_25907130	15.06	948	100	948	247	245
NODE_85208_length_948_cov_27.403_ID_25907444	119.33	948	100	948	509	508
NODE_85337_length_947_cov_2.58621_ID_25907702	19.85	947	100	947	200	202
NODE_85428_length_946_cov_1.84695_ID_25907884	28.22	946	100	946	371	358
NODE_85627_length_945_cov_2.58295_ID_25908280	16.64	945	100	945	174	175
NODE_85783_length_944_cov_1.42099_ID_25908588	16.13	944	93.4322	882	264	267
NODE_85797_length_944_cov_2.41522_ID_25908616	11.33	944	100	944	144	144
NODE_86168_length_943_cov_12.1051_ID_25909354	59.18	943	100	943	306	309
NODE_86263_length_942_cov_1.81734_ID_25909542	31.98	942	100	942	539	544
NODE_86366_length_942_cov_11.8786_ID_25909748	69.74	942	100	942	575	581
NODE_86557_length_941_cov_12.0266_ID_25910128	76.72	941	100	941	327	319
NODE_86766_length_940_cov_7.77636_ID_25910542	61.35	940	100	940	1102	1109
NODE_87064_length_938_cov_1.54123_ID_25911144	12.73	938	100	938	229	229
NODE_87137_length_938_cov_11.5389_ID_25911290	165.79	938	100	938	2101	2107
NODE_87168_length_937_cov_1.95233_ID_25911350	17.59	937	100	937	330	331
NODE_87172_length_937_cov_3.11279_ID_25911358	10.00	937	100	937	139	139
NODE_87903_length_934_cov_2.64411_ID_25912824	10.17	934	100	934	131	132
NODE_88609_length_930_cov_1.46307_ID_25914248	15.33	930	100	930	168	167
NODE_88755_length_930_cov_5.11958_ID_25914532	28.27	930	100	930	235	234
NODE_88827_length_929_cov_2.62324_ID_25914674	75.24	929	100	929	493	500
NODE_88855_length_929_cov_2.01056_ID_25914728	18.70	929	100	929	345	345
NODE_89375_length_926_cov_2.13899_ID_25915780	10.78	926	100	926	181	181
NODE_89502_length_926_cov_3.53004_ID_25916036	15.25	926	100	926	217	218
NODE_89621_length_925_cov_4.60377_ID_25916276	16.14	925	100	925	126	132
NODE_89898_length_924_cov_2.68477_ID_25916840	21.08	924	100	924	160	158
NODE_90037_length_923_cov_2.08629_ID_25917120	37.36	923	100	923	641	646
NODE_90405_length_921_cov_1.66469_ID_25917860	5.27	921	100	921	76	75
NODE_90414_length_921_cov_2.6173_ID_25917878	9.28	921	100	921	137	136
NODE_90433_length_921_cov_4.60545_ID_25917916	20.70	921	100	921	306	304
NODE_90464_length_921_cov_2.35427_ID_25917978	73.49	921	100	921	753	747
NODE_90484_length_921_cov_3.03791_ID_25918018	29.42	921	100	921	418	422
NODE_90494_length_921_cov_3.34479_ID_25918038	20.31	921	100	921	219	219
NODE_90654_length_920_cov_1.20996_ID_25918362	7.31	920	100	920	119	119
NODE_90717_length_920_cov_6.35706_ID_25918492	12.64	920	100	920	109	108

NODE_91059_length_918_cov_2.73246_ID_25919184	17.85	918	100	918	315	315
NODE_91162_length_918_cov_2.3912_ID_25919398	13.60	918	100	918	235	235
NODE_91744_length_915_cov_3.78401_ID_25920564	40.96	915	100	915	413	449
NODE_91814_length_915_cov_9.53699_ID_25920702	70.11	915	100	915	363	369
NODE_91849_length_914_cov_1.45639_ID_25920772	5.87	914	100	914	86	85
NODE_92021_length_914_cov_7.77778_ID_25921116	36.44	914	100	914	232	229
NODE_92208_length_913_cov_2.48206_ID_25921494	104.87	913	99.8905	912	440	436
NODE_92335_length_912_cov_4.27904_ID_25921748	50.61	912	100	912	418	423
NODE_92430_length_912_cov_2.32216_ID_25921938	56.74	912	100	912	640	651
NODE_92433_length_912_cov_1.56407_ID_25921944	16.41	912	100	912	244	246
NODE_92519_length_911_cov_1.74101_ID_25922114	8.14	911	100	911	127	127
NODE_92573_length_911_cov_1.84652_ID_25922230	18.80	911	100	911	110	114
NODE_92834_length_910_cov_3.7455_ID_25922772	19.68	910	100	910	313	315
NODE_92977_length_909_cov_1.78125_ID_25923062	14.04	909	100	909	230	231
NODE_93367_length_907_cov_2.68193_ID_25923854	13.98	907	100	907	240	240
NODE_93524_length_906_cov_1.04101_ID_25924164	27.01	906	100	906	527	526
NODE_93636_length_906_cov_1.60796_ID_25924390	75.64	906	100	906	827	837
NODE_93794_length_905_cov_2.07488_ID_25924706	18.30	905	100	905	342	341
NODE_93822_length_905_cov_2.6099_ID_25924760	29.53	905	97.5691	883	397	402
NODE_93933_length_905_cov_3.593_ID_25924982	50.99	905	100	905	361	364
NODE_94189_length_903_cov_2.67918_ID_25925492	27.50	903	100	903	417	423
NODE_94193_length_903_cov_2.46005_ID_25925500	10.57	903	100	903	166	164
NODE_94637_length_901_cov_2.66262_ID_25926394	7.80	901	100	901	101	102
NODE_94663_length_901_cov_2.96723_ID_25926446	15.86	901	100	901	259	257
NODE_95101_length_899_cov_4.23236_ID_25927324	16.37	899	100	899	233	233
NODE_95699_length_897_cov_9.19146_ID_25928532	65.62	897	100	897	316	300
NODE_95913_length_896_cov_9.5348_ID_25928964	27.18	896	100	896	145	142
NODE_95954_length_895_cov_3.60391_ID_25929050	14.34	895	100	895	219	218
NODE_96265_length_894_cov_3.06242_ID_25929672	16.34	894	100	894	237	237
NODE_96398_length_893_cov_2.00858_ID_25929934	11.95	893	100	893	176	177
NODE_96429_length_893_cov_4.84314_ID_25929996	16.26	893	100	893	223	222
NODE_96474_length_893_cov_3.42034_ID_25930086	12.12	893	100	893	155	154
NODE_96634_length_893_cov_4.40319_ID_25930400	36.67	893	100	893	296	304
NODE_96675_length_892_cov_1.86748_ID_25930482	7.01	892	100	892	107	107
NODE_96818_length_892_cov_2.4638_ID_25930770	13.19	892	100	892	205	206
NODE_97215_length_890_cov_5.24231_ID_25931580	25.15	890	100	890	192	194

NODE_97340_length_889_cov_4.20443_ID_25931838	27.42	889	100	889	367	367
NODE_97531_length_888_cov_1.62145_ID_25932222	21.69	888	100	888	400	404
NODE_97567_length_888_cov_3.67694_ID_25932294	12.75	888	100	888	147	147
NODE_97915_length_887_cov_1.86296_ID_25932994	12.13	887	100	887	188	187
NODE_97942_length_887_cov_2.10864_ID_25933048	81.79	887	100	887	541	557
NODE_98015_length_886_cov_1.46972_ID_25933196	6.85	886	100	886	95	97
NODE_99569_length_880_cov_3.36862_ID_25936292	26.06	880	100	880	269	261
NODE_99611_length_880_cov_6.37235_ID_25936372	30.31	880	100	880	151	150
NODE_99654_length_879_cov_2.08853_ID_25936458	13.89	879	100	879	221	221
NODE_99825_length_879_cov_7.40524_ID_25936802	18.40	879	100	879	124	126
NODE_100035_length_878_cov_2.6005_ID_25937218	86.66	878	100	878	407	399
NODE_100337_length_877_cov_5.925_ID_25937820	28.77	877	100	877	284	277
NODE_100410_length_876_cov_2.91364_ID_25937966	32.01	876	100	876	529	525
NODE_100417_length_876_cov_1.65957_ID_25937980	12.65	876	100	876	197	195
NODE_100681_length_875_cov_1.84085_ID_25938514	36.80	875	100	875	459	444
NODE_100870_length_874_cov_2.97742_ID_25938896	43.54	874	100	874	417	422
NODE_101181_length_873_cov_3.1093_ID_25939520	13.26	873	100	873	141	143
NODE_101372_length_872_cov_2.39623_ID_25939906	9.16	872	100	872	126	126
NODE_101409_length_872_cov_3.13082_ID_25939980	13.15	872	100	872	180	180
NODE_101557_length_872_cov_2.60503_ID_25940278	23.96	872	100	872	220	220
NODE_101751_length_871_cov_3.40806_ID_25940666	8.29	871	100	871	76	76
NODE_101756_length_871_cov_3.93829_ID_25940676	29.97	871	100	871	197	197
NODE_102023_length_870_cov_4.77175_ID_25941208	20.48	870	100	870	255	249
NODE_102120_length_869_cov_1.60354_ID_25941402	14.10	869	100	869	233	231
NODE_102148_length_869_cov_2.28157_ID_25941458	18.30	869	100	869	325	325
NODE_102201_length_869_cov_3.17677_ID_25941572	14.13	869	100	869	206	207
NODE_102914_length_866_cov_4.82003_ID_25943020	12.52	866	100	866	134	137
NODE_102942_length_866_cov_1.70089_ID_25943076	17.49	866	100	866	298	299
NODE_103151_length_865_cov_3.43782_ID_25943492	18.42	865	100	865	194	195
NODE_103760_length_863_cov_2.78753_ID_25944706	21.61	863	100	863	242	240
NODE_104712_length_859_cov_2.91816_ID_25946624	47.72	859	100	859	624	628
NODE_105003_length_858_cov_2.4443_ID_25947208	7.08	858	100	858	82	81
NODE_105903_length_855_cov_3.41517_ID_25949014	145.07	855	100	855	1250	1269
NODE_106417_length_853_cov_2.85954_ID_25950046	92.55	853	100	853	880	875
NODE_106477_length_853_cov_3.06314_ID_25950166	14.92	853	100	853	188	186
NODE_107610_length_848_cov_2.08171_ID_25952446	12.16	848	100	848	92	92

NODE_107696_length_848_cov_1.50843_ID_25952622	14.37	848	100	848	218	216
NODE_108107_length_846_cov_0.989597_ID_25953440	8.16	846	79.3144	671	165	165
NODE_108168_length_846_cov_1.6632_ID_25953568	5.50	846	100	846	71	71
NODE_108213_length_846_cov_2.27308_ID_25953658	35.85	846	100	846	188	189
NODE_108214_length_846_cov_1.72822_ID_25953660	17.96	846	100	846	307	307
NODE_108361_length_845_cov_1.73568_ID_25953952	56.20	845	100	845	678	692
NODE_108519_length_845_cov_2.10026_ID_25954276	30.96	845	100	845	467	469
NODE_108763_length_844_cov_4.41851_ID_25954762	18.09	844	100	844	181	182
NODE_109611_length_841_cov_2.5589_ID_25956462	15.23	841	100	841	195	197
NODE_109922_length_840_cov_3.92923_ID_25957088	28.22	840	100	840	368	368
NODE_109945_length_840_cov_1.67104_ID_25957134	8.46	840	100	840	95	96
NODE_110004_length_840_cov_2.25426_ID_25957252	14.84	840	100	840	220	221
NODE_110027_length_840_cov_2.04063_ID_25957298	17.87	840	100	840	246	247
NODE_111000_length_836_cov_2.60606_ID_25959258	54.19	836	100	836	196	192
NODE_111216_length_835_cov_1.73747_ID_25959692	111.69	835	100	835	1078	1071
NODE_111300_length_835_cov_2.38127_ID_25959860	16.16	835	100	835	262	263
NODE_111503_length_834_cov_2.78468_ID_25960266	46.78	834	100	834	413	416
NODE_112175_length_832_cov_1.69669_ID_25961618	16.91	832	100	832	253	255
NODE_112727_length_830_cov_1.30146_ID_25962722	10.64	830	100	830	146	143
NODE_112815_length_830_cov_2.46746_ID_25962896	19.61	830	100	830	273	276
NODE_112922_length_829_cov_1.05186_ID_25963110	8.53	829	100	829	130	131
NODE_113174_length_829_cov_6.34973_ID_25963614	30.31	829	100	829	183	186
NODE_113916_length_826_cov_3.08144_ID_25965094	47.82	826	100	826	711	733
NODE_114041_length_825_cov_2.24332_ID_25965344	8.17	825	100	825	78	80
NODE_114169_length_825_cov_2.95989_ID_25965594	76.90	825	100	825	307	315
NODE_114360_length_824_cov_2.54886_ID_25965978	27.55	824	100	824	450	450
NODE_114663_length_823_cov_1.82038_ID_25966586	7.79	823	100	823	119	119
NODE_114779_length_823_cov_2.73458_ID_25966812	17.70	823	100	823	198	196
NODE_114958_length_822_cov_1.96376_ID_25967172	37.47	822	100	822	587	594
NODE_115103_length_822_cov_2.14228_ID_25967462	21.31	822	100	822	353	350
NODE_115527_length_820_cov_1.46568_ID_25968304	784.97	820	100	820	11806	256
NODE_115735_length_820_cov_2.15747_ID_25968724	17.84	820	100	820	262	259
NODE_116629_length_817_cov_2.72973_ID_25768868	7.96	817	100	817	74	76
NODE_116920_length_816_cov_1.66982_ID_25971076	6.00	816	100	816	69	69
NODE_117987_length_812_cov_2.71156_ID_25973210	17.74	812	100	812	193	192
NODE_118142_length_812_cov_12.2748_ID_25973518	151.87	812	100	812	506	496

NODE_118208_length_811_cov_1.75068_ID_25973648	301.58	811	100	811	1836	1719
NODE_118223_length_811_cov_1.74659_ID_25973678	12.84	811	100	811	207	207
NODE_118441_length_811_cov_3.93052_ID_25974106	16.68	811	100	811	95	95
NODE_118462_length_811_cov_1.85014_ID_25974146	68.64	811	100	811	1056	1077
NODE_118827_length_810_cov_6.4352_ID_25974880	24.75	810	100	810	237	174
NODE_118848_length_809_cov_1.87022_ID_25974922	9.70	809	100	809	129	128
NODE_119115_length_809_cov_1.72541_ID_25975454	41.62	809	100	809	232	220
NODE_119188_length_808_cov_3.36662_ID_25975600	17.28	808	100	808	183	184
NODE_119210_length_808_cov_1.19015_ID_25975648	26.50	808	100	808	333	343
NODE_119411_length_808_cov_2.8933_ID_25976050	21.18	808	100	808	326	330
NODE_119647_length_807_cov_2.6274_ID_25976526	71.71	807	100	807	587	599
NODE_119731_length_807_cov_1.74247_ID_25976694	15.37	807	100	807	263	263
NODE_119892_length_806_cov_2.3155_ID_25977020	17.83	806	100	806	263	265
NODE_119942_length_806_cov_1.28258_ID_25977118	15.47	806	100	806	235	235
NODE_119966_length_806_cov_1.64335_ID_25977166	9.37	806	100	806	113	113
NODE_120014_length_806_cov_3.03567_ID_25977262	159.93	806	99.7519	804	748	2509
NODE_120210_length_805_cov_8.03297_ID_25977654	64.58	805	100	805	268	258
NODE_120686_length_804_cov_4.69876_ID_25978602	25.33	804	98.5075	792	179	180
NODE_120776_length_803_cov_33.7273_ID_25978780	196.52	803	100	803	707	717
NODE_121152_length_802_cov_1.42759_ID_25979546	26.63	802	100	802	415	421
NODE_121191_length_802_cov_2.12552_ID_25979624	21.97	802	100	802	332	331
NODE_121202_length_802_cov_1.94345_ID_25979646	7.22	802	100	802	111	112
NODE_121846_length_800_cov_3.03181_ID_25980930	15.67	800	100	800	202	200
NODE_121861_length_800_cov_1.48548_ID_25980960	11.21	800	100	800	176	177
NODE_121890_length_800_cov_1.52144_ID_25981018	34.76	800	100	800	206	199
NODE_121906_length_800_cov_2.94744_ID_25981050	46.63	800	100	800	225	221
NODE_122014_length_800_cov_15.2061_ID_25981266	142.70	800	100	800	616	626
Average reads coverage for the assembled genome	51.2					

Table S16. Comparative analysis of 15 COG functions most abundant in ZC3 and ZC4 metagenomes

		Composting cell	D01		D30		D64		D78		D99	
COG category	COG functions	Name	ZC3	ZC4	ZC3	ZC4	ZC3	ZC4	ZC3	ZC4	ZC3	ZC4
L	COG0178	Excinuclease UvrABC ATPase subunit	2.64	1.65	1.43	1.36	1.45	1.34	1.69	1.23	1.91	1.13
L	COG0187	DNA gyrase/topoisomerase IV, subunit B	2.72	1.27	1.19	0.81	1.17	1.09	1.22	0.88	1.19	0.78
L	COG0188	DNA gyrase/topoisomerase IV, subunit A	2.67	1.63	1.23	1.22	1.32	1.48	1.61	1.16	1.62	1.08
I,Q	COG0318	Acyl-CoA synthetase (AMP-forming)/AMP-acid ligase II	2.86	3.53	3.53	3.99	3.60	3.61	3.68	3.57	3.26	4.15
G	COG0395	ABC-type glycerol-3-phosphate transport system, permease component	1.65	2.91	3.38	2.54	2.83	2.77	2.29	2.64	2.28	2.78
M	COG0438	Glycosyltransferase involved in cell wall biosynthesis	2.65	2.56	3.26	4.80	3.40	3.72	3.89	4.43	3.05	5.06
J	COG0480	Translation elongation factor EF-G, a GTPase	2.79	1.16	1.16	0.98	1.28	1.16	1.27	1.08	1.12	0.95
K,L,R,T	COG0515	Serine/threonine protein kinase	1.25	1.52	2.43	3.54	1.74	2.08	2.92	2.89	2.01	3.53
V	COG0534	Na+-driven multidrug efflux pump	5.17	2.58	1.59	1.03	1.34	1.6	1.18	1.32	1.17	1.09
E,P	COG0601	ABC-type dipeptide/oligopeptide/nickel transport system, permease component	1.20	2.05	2.95	2.38	3.11	2.3	2.47	2.67	2.38	2.75
J	COG0621	tRNA A37 methylthiotransferase MiaB	2.59	1.14	1.13	1.11	1.08	1.13	1.06	0.97	1.14	0.94
T	COG0642	Signal transduction histidine kinase	4.15	3.45	3.11	3.87	2.92	3.64	3.29	3.41	3.51	3.86
R	COG0673	Predicted dehydrogenase	2.34	2.70	3.44	3.15	2.91	2.71	2.85	3.17	2.92	3.44
K,T	COG0745	DNA-binding response regulator, OmpR family, contains REC and winged-helix (wHTH) domain	3.47	3.36	3.58	3.67	2.93	3.57	3.38	3.21	3.27	3.13
E	COG0747	ABC-type transport system, periplasmic component	1.17	2.76	4.10	3.45	3.66	3.12	3.52	3.78	3.08	3.71
V	COG0841	Multidrug efflux pump subunit AcrB	2.80	3.57	3.90	3.74	3.83	3.56	3.89	3.55	4.63	3.72
C	COG1012	Acyl-CoA reductase or other NAD-dependent aldehyde dehydrogenase	1.43	4.26	4.16	4.19	4.02	3.87	4.04	3.56	4.33	3.72
I,Q,R	COG1028	NAD(P)-dependent dehydrogenase, short-chain	2.06	4.05	4.22	4.32	3.71	4.14	3.98	4.06	4.10	4.60

		alcohol dehydrogenase family										
V	COG1131	ABC-type multidrug transport system, ATPase component	1.73	2.52	2.78	2.85	3.10	2.98	3.29	2.93	2.76	2.63
V	COG1132	ABC-type multidrug transport system, ATPase and permease component	4.62	3.75	3.61	2.79	2.99	3.64	3.16	2.98	2.90	2.72
G	COG1175	ABC-type sugar transport system, permease component	1.47	2.68	3.42	2.66	2.80	2.71	2.45	2.65	2.42	2.87
R	COG1373	Predicted ATPase, AAA+ superfamily	2.76	0.55	0.24	0.19	0.24	0.17	0.20	0.17	0.25	0.24
K	COG1595	DNA-directed RNA polymerase specialized sigma subunit, sigma24 family	2.91	2.94	3.25	3.57	2.92	3.15	2.95	3.16	2.70	3.87
K	COG1609	DNA-binding transcriptional regulator, LacI/PurR family	1.92	3.68	3.35	2.74	2.65	3.18	2.70	2.59	2.35	2.74
G	COG1653	ABC-type glycerol-3-phosphate transport system, periplasmic component	1.36	2.69	4.68	2.93	3.70	3.17	3.04	4.22	2.95	3.39
I	COG1960	Acyl-CoA dehydrogenase related to the alkylation response protein AidB	1.14	3.30	3.63	4.10	3.88	3.41	4.06	3.68	3.70	3.76
T	COG2204	DNA-binding transcriptional response regulator, NtrC family, contains REC, AAA-type ATPase, and a Fis-type DNA-binding domains	1.45	2.13	2.50	3.71	2.61	2.82	3.37	3.24	3.26	3.77
P	COG2217	Cation transport ATPase	2.38	2.5	2.62	2.27	2.86	2.47	2.74	2.33	2.81	2.09
L	COG4974	Site-specific recombinase XerD	3.46	2.45	1.96	2.08	2.36	2.15	2.37	2.55	2.40	2.36
K	COG0583	DNA-binding transcriptional regulator, LysR family	1.82	4.84	2.49	2.43	1.99	3.21	1.81	1.98	1.99	2.25
K,T	COG2197	DNA-binding response regulator, NarL/FixJ family, contains REC and HTH domains	1.04	2.26	2.91	2.74	2.65	2.41	2.76	2.67	2.41	3.00
G	COG2814	Predicted arabinose efflux permease, MFS family	1.11	3.67	2.79	2.65	2.18	2.76	2.08	2.42	2.33	2.49

Relative values (‰= 1/1000).

COG Categories: C: Energy production and conversion, E: Amino acid transport and metabolism, G: Carbohydrate transport and metabolism, I: Lipid transport and metabolism, J: Translation, ribosomal structure and biogenesis, K: Transcription, L: Replication, recombination and repair, M: Cell wall/membrane/envelope biogenesis, P: Inorganic ion transport and metabolism, Q: Secondary metabolites biosynthesis, transport and catabolism, R: General function prediction only, T: Signal transduction mechanisms, V: Defense mechanisms

Table S17. COG analysis related to biomass degradation in ZC4 metatranscriptome

Enzyme	EC number	COG	Function name	D01	D03	D07	D15	D30	D64	D78	D99
Cellulase	3.2.1.21	COG2723	Beta-glucosidase/6-phospho-beta-glucosidase/beta-galactosidase	0.0545	0.0560	0.0705	0.0701	0.0656	0.0222	0.0567	0.1136
	3.2.1.91	COG3405	Endo-1,4-beta-D-glucanase Y	0.0063	0.0031	0.0038	0.0091	0.0159	0.0016	0.0038	0.0159
	3.2.1.4	COG5297	Cellulase/cellobiase CelA1	0.0524	0.0448	0.0534	0.0584	0.1069	0.0135	0.1662	0.0681
Hemicellulase	3.2.1.8	COG3693	Endo-1,4-beta-xylanase, GH35 family	0.0440	0.0468	0.0610	0.0935	0.1090	0.0421	0.1058	0.0273
	3.2.1.37	COG3507	Beta-xylosidase	0.0545	0.0244	0.0486	0.0597	0.0593	0.0349	0.0378	0.0409
	3.2.1.55	COG3534	Alpha-L-arabinofuranosidase	0.0346	0.0407	0.0410	0.0428	0.0519	0.0238	0.0397	0.0023
	3.2.1.139	COG3661	Alpha-glucuronidase	0.0105	0.0132	0.0200	0.0130	0.0159	0.0103	0.0019	0.0045
	3.2.1.78	COG3934	Endo-1,4-beta-mannosidase	0.0000	0.0031	0.0010	0.0052	0.0000	0.0000	0.0019	0.0068
	3.2.1.22	COG3345	Alpha-galactosidase	0.0147	0.0143	0.0076	0.0065	0.0042	0.0095	0.0019	0.0114
	3.2.1.21	COG2723	Beta-glucosidase/6-phospho-beta-glucosidase/beta-galactosidase	0.0545	0.0560	0.0705	0.0701	0.0656	0.0222	0.0567	0.1136
	3.2.1.89	COG3867	Arabinogalactan endo-1,4-beta-galactosidase	0.0031	0.0010	0.0029	0.0026	0.0053	0.0024	0.0057	0.0045
	3.2.1.23	COG3250	Beta-galactosidase/beta-glucuronidase	0.0482	0.0295	0.0858	0.0662	0.0614	0.0206	0.0283	0.0204
Ligninase	1.10.3.2	COG1496	Copper oxidase (laccase) domain	0.0210	0.0326	0.0343	0.0363	0.0328	0.0206	0.0113	0.0273
	1.1.99.18	COG2303	Choline dehydrogenase or related flavoprotein	0.0157	0.0153	0.0229	0.0441	0.0476	0.0183	0.0321	0.0863
	1.11.1.19	COG2837	Periplasmic deferrochelatase/peroxidase EfeB	0.0105	0.0061	0.0057	0.0013	0.0042	0.0087	0.0019	0.0204
	1.3.3.5.	COG2132	Multicopper oxidase with three cupredoxin domains (includes cell division protein FtsP and spore coat protein CotA)	0.0262	0.0346	0.0686	0.0973	0.0656	0.0714	0.1077	0.2090

	3.1.1.11	COG4677	Pectin methylesterase and related acyl-CoA thioesterases	0.0021	0.0031	0.0038	0.0039	0.0032	0.0000	0.0038	0.0091
Pectinase	3.2.1.82	COG5434	Polygalacturonase	0.0136	0.0051	0.0095	0.0052	0.0106	0.0056	0.0076	0.0091
	4.2.2.2	COG3866	Pectate lyase	0.0178	0.0071	0.0038	0.0065	0.0127	0.0008	0.0057	0.0068

Relative abundance of CDSs annotated with given COGs by the IMG/M pipeline. Relative values (%).

Table S18. Number of CDSs in ZC4 metatranscriptome classified by CAZy database

CAZy families	CAZy enzymes	D01	D03	D07	D15	D30	D64	D78	D99	CAZy classification
Cellulosome	SLH	393	302	255	188	201	536	132	21	domains of the scaffolding proteins in cellulosome
	cohesin	10	15	4	4	5	19	1	0	duplicated sequences presents in enzymes and cellulosome
	dockerin	4	8	1	0	6	6	3	0	non-catalytic scaffolding protein containing repeated sequences presents in enzymes and cellulosome
CBM	CBM1	0	0	1	0	1	0	0	0	modules almost exclusively in fungi. The only non-fungal occurence of CBM1 is in an algal non-hydrolytic polysaccharide-binding protein
	CBM2	19	19	26	21	43	8	47	14	modules found in a large number of bacterial enzymes. Bind cellulose, quitin and xylan
	CBM3	9	6	3	2	6	8	4	0	modules found in bacterial enzymes. Bind cellulose
	CBM4	5	8	3	7	9	18	10	0	modules found in bacterial enzymes. Bind xylan, β-1,3-glucan, β-1,3-1,4-glucan, β-1,6-glucan and amorphous cellulose
	CBM5	3	3	0	3	1	2	1	0	modules found in bacterial enzymes. Chitin-binding described in several cases.
	CBM6	15	19	12	11	15	18	9	1	bind cellulose
	CBM8	2	1	1	3	0	2	0	0	the cellulose-binding module from a cellulase of the slime mold *Dictyostelium discoideum*
	CBM9	6	11	6	10	17	20	17	1	modules found only in xylanases
	CBM11	0	2	2	2	0	0	0	0	bind both β-1,4-glucan and β-1,3-1,4-mixed linked glucans
	CBM12	6	2	1	0	1	1	1	1	bind chitin
	CBM13	6	2	5	4	6	5	8	2	found in glycoside hydrolases and glycosyltransferases
	CBM14	0	1	0	0	0	1	0	2	bind chitin
	CBM15	2	0	0	0	0	1	0	0	bind xylan and xylooligosaccharides
	CBM16	12	14	14	17	18	11	8	3	bind cellulose and glucomannan
	CBM17	0	1	0	1	0	1	0	0	bind amorphous cellulose, cellooligosaccharides and derivatized cellulose
	CBM18	1	0	0	0	0	0	1	0	bind chitin
	CBM19	1	0	0	0	0	0	0	0	bind chitin
	CBM20	6	7	9	6	3	8	3	1	bind starch
	CBM21	0	0	0	0	0	1	0	0	bind starch

CBM22	8	5	11	8	16	14	9	2	bind xylan
CBM23	0	1	1	1	5	5	0	0	bind mannan
CBM24	0	0	0	0	0	0	1	0	bind α-1,3-glucan
CBM25	6	4	5	4	8	3	7	3	bind starch
CBM26	4	2	1	0	0	3	0	0	bind starch
CBM27	0	0	0	1	0	1	0	0	bind mannan
CBM28	0	0	0	0	0	1	0	0	bind non-crystalline cellulose, cellooligosaccharide and β-(1,3)(1,4)-glucan
CBM30	1	1	1	1	1	5	2	0	bind cellulose
CBM31	0	1	0	0	0	0	0	0	bind β-1,3-xylan
CBM32	26	18	27	22	22	43	14	9	bind galactose and lactose
CBM34	8	15	15	8	2	9	1	1	bind starch
CBM35	11	15	19	16	20	19	8	6	bind xylan
CBM36	2	3	1	2	0	4	1	0	bind xylan and xylooligosaccharide
CBM37	11	5	3	6	2	8	1	1	bind xylan, chitin, microcrystalline and phosphoric-acid swollen cellulose
CBM38	0	1	0	0	0	0	0	0	bind inulin
CBM39	1	1	0	0	0	1	0	0	bind β-1,3-glucan
CBM40	0	8	4	8	5	4	6	3	bind sialic acid
CBM41	4	0	1	0	2	3	0	0	bind α-glucans amylose, amylopectin, pullulan and oligosaccharide
CBM42	2	0	0	0	0	0	0	0	bind arabinofuranose (present in arabinoxylan)
CBM43	0	1	2	1	0	2	0	0	found at the C-terminus of GH17 or GH72. Bind β-1,3-glucan
CBM44	17	23	10	11	15	4	12	2	bind cellulose and xyloglucan
CBM46	3	7	6	3	4	11	1	0	found at the C-terminus of several GH5 cellulases. Bind cellulose
CBM47	0	0	0	1	1	0	0	0	bind fucose
CBM48	9	3	10	4	13	5	8	7	found in GH13. Bind glycogen
CBM49	0	0	0	0	0	0	1	0	found in GH9. Bind crystalline cellulose
CBM50	206	250	220	169	198	291	156	71	found in GH18, GH19, GH23, GH24, GH25 and GH73. Bind chitin and peptidoglycan
CBM51	2	1	3	0	1	2	0	0	found in GH2, GH27, GH31, GH95, GH98 and GH101 . Bind galactose
CBM53	1	0	0	1	0	1	0	0	bind starch
CBM54	4	3	3	2	5	6	3	0	bind xylan, yeast cell wall glucan and chitin
CBM56	4	0	0	2	3	3	4	0	bind β-1,3-glucan

	CBM57	1	2	0	3	2	2	1	0	found in glycosidases
	CBM58	0	2	0	0	0	1	0	0	bind maltoheptaose
	CBM59	3	2	3	4	4	4	2	0	bind mannan, xylan and cellulose
	CBM60	1	3	0	0	1	0	0	0	found in xylanases. Bind xylan
	CBM61	3	6	5	4	2	4	1	0	found in GH16, GH30, GH31, GH43, GH53 and GH66. Bind β-1,4-galactan
	CBM62	0	0	0	0	0	1	1	0	found on xyloglucan, arabinogalactan and galactomannan
	CBM63	0	1	0	1	0	0	1	0	bind cellulose
	CBM64	0	1	0	0	0	0	0	0	bind cellulose
	CBM65	0	2	0	1	1	3	0	0	bind β-glucans
	CBM66	9	16	7	14	9	20	5	1	bind fructoside residue
	CBM67	7	6	7	5	3	1	3	0	bind L-rhamnose
GH	GH1	12	14	17	19	13	7	7	9	β-glucosidase; β-galactosidase; β-mannosidase; β-glucuronidase; β-xylosidase; β-D-fucosidase
	GH2	2	2	4	4	2	0	0	1	β-galactosidase; β-mannosidase; β-glucuronidase; α-L-arabinofuranosidase; mannosylglycoprotein endo-β-mannosidase; exo-β-glucosaminidase
	GH3	24	28	32	32	29	31	14	8	β-glucosidase; xylan 1,4-β-xylosidase; β-glucosylceramidase; β-N-acetylhexosaminidase; α-L-arabinofuranosidase; glucan 1,3-β-glucosidase; glucan 1,4-β-glucosidase
	GH4	17	24	17	22	11	26	8	8	maltose-6-phosphate glucosidase; α-glucosidase; α-galactosidase; 6-phospho-β-glucosidase; α-glucuronidase; α-galacturonase
	GH5	12	10	7	10	13	11	15	3	endo-β-1,4-glucanase; endo-β-1,4-xylanase; β-glucosidase; β-mannosidase; β-glucosylceramidase; glucan β-1,3-glucosidase; exo-β-1,4-glucanase; glucan endo-1,6-β-glucosidase; mannan endo-β-1,4-mannosidase; cellulose β-1,4-cellobiosidase
	GH6	8	9	7	11	12	3	8	4	endoglucanase; cellobiohydrolase
	GH7	1	1	0	0	1	0	0	0	endo-β-1,4-glucanase; reducing end-acting cellobiohydrolase; chitosanase; endo-β-1,3-1,4-glucanase
	GH8	1	1	1	2	6	1	1	4	chitosanase; cellulase; licheninase; endo-1,4-β-xylanase; reducing-end-xylose releasing exo-oligoxylanase
	GH9	2	2	1	4	5	6	3	1	endoglucanase; endo-β-1,3(4)-glucanase; β-glucosidase; endo-β-1,3-1,4-glucanase; exo-β-1,4-glucanase; cellobiohydrolase; endo-xyloglucanase; exo-β-glucosaminidase
	GH10	22	21	28	35	35	25	24	3	endo-1,4-β-xylanase; endo-1,3-β-xylanase; tomatinase; xylan endotransglycosylase

GH11	16	15	13	5	10	17	4	2	endo-β-1,4-xylanase; endo-β-1,3-xylanase
GH12	3	2	3	4	6	1	5	0	endoglucanase; xyloglucan hydrolase; β-1,3-1,4-glucanase; xyloglucan endotransglycosylase
GH13	47	44	34	22	23	52	10	13	α-amylase; pullulanase; cyclomaltodextrin glucanotransferase; cyclomaltodextrinase; trehalose-6-phosphate hydrolase; oligo-α-glucosidase
GH15	13	9	19	9	10	11	5	2	glucoamylase; glucodextranase; α,α-trehalase; dextran dextrinase
GH16	7	2	3	3	5	10	3	2	xyloglucan:xyloglucosyltransferase; keratan-sulfate endo-1,4-β-galactosidase; endo-1,3-β-glucanase; endo-1,3(4)-β-glucanase; licheninase; β-agarase
GH17	0	1	0	0	0	2	0	0	glucan endo-1,3-β-glucosidase; glucan 1,3-β-glucosidase; licheninase ; ABA-specific β-glucosidase; β-1,3-glucanosyltransglycosylase
GH18	25	23	30	18	5	26	3	2	chitinase; lysozyme; endo-β-N-acetylglucosaminidase; peptidoglycan hydrolase with endo-β-N-acetylglucosaminidase specificity
GH19	1	0	1	1	2	0	2	1	chitinase; lysozyme
GH20	1	0	1	2	0	1	0	1	β-hexosaminidase; lacto-N-biosidase; β-1,6-N-acetylglucosaminidase; β-6-SO3-N-acetylglucosaminidase
GH22	2	0	0	2	2	1	1	0	lysozyme type C; lysozyme type i; α-lactalbumin
GH23	29	49	51	26	41	44	16	26	lysozyme type G; peptidoglycan lyase; chitinase
GH24	3	3	4	2	3	1	3	3	lysozyme
GH25	7	3	4	4	3	8	3	2	lysozyme
GH26	1	2	4	7	4	2	5	0	β-mannanase; exo-β-1,4-mannobiohydrolase; β-1,3-xylanase; endo-β-1,3-1,4-glucanase ; mannobiose-producing exo-β-mannanase
GH27	0	0	0	1	1	2	1	0	α-galactosidase; α-N-acetylgalactosaminidase; isomalto-dextranase; β-L-arabinopyranosidase; galactan:galactan galactosyltransferase
GH28	3	1	3	1	0	4	2	0	polygalacturonase; exo-polygalacturonase; exo-polygalacturonosidase; rhamnogalacturonase; rhamnogalacturonan α-1,2-galacturonohydrolase
GH29	2	1	5	3	1	1	0	0	α-L-fucosidase; α-1,3/1,4-L-fucosidase
GH30	0	6	6	2	1	1	2	0	endo-β-1,4-xylanase; β-glucosidase; β-glucuronidase; β-xylosidase; β-fucosidase; glucosylceramidase; β-1,6-glucanase
GH31	8	4	20	12	6	2	2	5	α-glucosidase; α-galactosidase; α-mannosidase; α-1,3-glucosidase; sucrase-isomaltase; α-xylosidase
GH32	4	0	1	2	3	1	0	1	invertase; endo-inulinase; β-2,6-fructan 6-levanbiohydrolase; endo-levanase; exo-inulinase; fructan β-(2,1)-fructosidase; fructan β-

									(2,6)-fructosidase/6-exohydrolase
GH33	4	2	1	2	5	0	2	0	sialidase; trans-sialidase; 2-keto-3-deoxynononic acid hydrolase; anhydrosialidase; 3-deoxy-D-manno-octulosonic-acid hydrolase
GH35	5	2	4	8	4	2	3	3	β-galactosidase; exo-β-glucosaminidase; exo-β-1,4-galactanase; β-1,3-galactosidase
GH36	0	2	1	0	0	1	0	1	α-galactosidase; α-N-acetylgalactosaminidase; stachyose synthase; raffinose synthase
GH37	0	0	0	0	0	0	0	3	α,α-trehalase
GH38	2	2	5	2	2	2	0	0	α-mannosidase; mannosyl-oligosaccharide α-1,2-mannosidase; mannosyl-oligosaccharide α-1,3-1,6-mannosidase; α-2-O-mannosylglycerate hydrolase; mannosyl-oligosaccharide α-1,3-mannosidase
GH39	1	2	4	1	0	2	1	0	α-L-iduronidase; β-xylosidase
GH42	9	5	7	6	1	3	1	2	β-galactosidase; α-L-arabinopyranosidase
GH43	36	15	25	24	28	31	11	5	β-xylosidase; α-L-arabinofuranosidase; arabinanase; xylanase; galactan 1,3-β-galactosidase; α-1,2-L-arabinofuranosidase
GH44	1	0	0	1	1	0	0	0	endoglucanase; xyloglucanase
GH45	0	1	1	0	1	0	0	0	endoglucanase
GH48	5	5	3	3	2	5	4	1	reducing end-acting cellobiohydrolase; endo-β-1,4-glucanase; chitinase
GH51	1	2	5	8	4	2	1	0	endoglucanase; endo-β-1,4-xylanase; β-xylosidase; α-L-arabinofuranosidase
GH52	0	0	1	0	1	1	0	0	β-xylosidase
GH53	1	1	0	2	3	1	0	1	endo-β-1,4-galactanase
GH54	0	1	1	0	0	0	0	0	α-L-arabinofuranosidase; β-xylosidase
GH57	1	3	8	5	4	1	1	1	α-amylase; α-galactosidase; amylopullulanase; cyclomaltodextrinase; branching enzym; 4-α-glucanotransferase
GH62	2	1	1	2	1	0	3	0	α-L-arabinofuranosidase
GH64	1	0	0	0	0	1	0	0	β-1,3-glucanase
GH65	9	6	4	2	0	4	3	1	α,α-trehalase; maltose phosphorylase; trehalose phosphorylase; kojibiose phosphorylase; trehalose-6-phosphate phosphorylase
GH66	1	0	1	0	0	1	0	0	α,α-trehalase; maltose phosphorylase; trehalose phosphorylase; kojibiose phosphorylase; trehalose-6-phosphate phosphorylase
GH67	0	2	5	1	2	2	0	0	α-glucuronidase; xylan α-1,2-glucuronidase
GH68	0	0	0	0	0	3	0	0	levansucrase; β-fructofuranosidase; inulosucrase
GH72	0	0	0	0	0	3	0	0	β-1,3-glucanosyltransglycosylase

GH73	21	16	6	7	8	25	2	5	lysozyme; mannosyl-glycoprotein endo-β-N-acetylglucosaminidase; peptidoglycan hydrolase with endo-β-N-acetylglucosaminidase specificity
GH74	4	8	17	16	15	9	7	11	endoglucanase; oligoxyloglucan reducing end-specific cellobiohydrolase; xyloglucanase
GH75	1	0	0	0	0	0	0	0	chitosanase
GH76	0	0	1	1	1	3	0	0	α-1,6-mannanase
GH77	1	2	0	1	1	1	1	1	amylomaltase or 4-α-glucanotransferase
GH78	3	1	5	7	0	2	0	1	α-L-rhamnosidase
GH81	0	1	0	0	3	0	0	0	endo-β-1,3-glucanase
GH82	0	0	0	0	2	0	1	0	I-carrageenase
GH84	1	1	1	1	1	2	0	0	N-acetyl β-glucosaminidase; hyaluronidase; [protein]-3-O-(GlcNAc)-L-Ser/Thr β-N-acetylglucosaminidase
GH87	4	0	0	0	0	0	0	0	mycodextranase; α-1,3-glucanase
GH88	2	0	0	1	2	0	1	0	d-4,5-unsaturated β-glucuronyl hydrolase
GH92	0	0	1	3	0	0	0	0	mannosyl-oligosaccharide α-1,2-mannosidase; mannosyl-oligosaccharide α-1,3-mannosidase; mannosyl-oligosaccharide α-1,6-mannosidase; α-mannosidase; α-1,2-mannosidase; α-1,3-mannosidase; α-1,4-mannosidase
GH93	0	0	0	1	1	0	0	1	exo-α-L-1,5-arabinanase
GH94	2	1	2	0	0	2	1	0	cellobiose phosphorylase; laminaribiose phosphorylase; cellodextrin phosphorylase; chitobiose phosphorylase; cyclic β-1,2-glucan synthase; cellobionic acid phosphorylase
GH95	3	0	1	1	1	0	1	0	α-L-fucosidase; α-1,2-L-fucosidase; α-L-galactosidase
GH97	0	0	0	1	2	0	2	0	glucoamylase; α-glucosidase; α-galactosidase
GH99	1	0	1	2	1	0	1	0	glycoprotein endo-α-1,2-mannosidase
GH102	0	1	1	0	0	0	0	3	peptidoglycan lytic transglycosylase
GH103	2	1	0	0	1	5	0	7	peptidoglycan lytic transglycosylase
GH104	0	1	0	0	0	1	0	0	peptidoglycan lytic transglycosylase
GH105	6	0	0	2	1	2	0	3	unsaturated rhamnogalacturonyl hydrolase; d-4,5-unsaturated β-glucuronyl hydrolase
GH106	2	0	0	2	0	0	0	0	α-L-rhamnosidase
GH108	2	1	3	1	2	0	0	1	N-acetylmuramidase
GH109	94	81	103	95	132	99	73	27	α-N-acetylgalactosaminidase
GH113	2	1	0	0	1	0	0	0	β-mannanase

	GH114	0	1	1	0	0	0	1	1	endo-α-1,4-polygalactosaminidase
	GH115	2	1	2	0	3	0	1	0	xylan α-1,2-glucuronidase; α-(4-O-methyl)-glucuronidase
	GH116	1	1	0	0	1	0	1	0	β-glucosidase; β-xylosidase; acid β-glucosidase; β-N-acetylglucosaminidase
	GH117	2	0	0	0	1	0	1	0	α-1,3-L-neoagarooligosaccharide hydrolase; α-1,3-L-neoagarobiase
	GH120	3	4	3	3	1	4	1	0	β-xylosidase
	GH123	0	0	0	1	0	0	0	0	glycosphingolipid β-N-acetylgalactosaminidase
	GH125	1	0	2	2	0	1	0	0	exo-α-1,6-mannosidase
	GH126	1	0	0	0	0	1	0	0	α-amylase
	GH127	4	2	5	7	1	4	1	2	β-L-arabinofuranosidase
	GH128	0	0	0	0	0	1	0	0	β-1,3-glucanase
	GH129	2	0	1	0	1	0	1	0	α-N-acetylgalactosaminidase
	GH130	21	20	29	19	13	28	5	0	β-1,4-mannosylglucose phosphorylase; β-1,4-mannooligosaccharide phosphorylase; β-1,4-mannosyl-N-acetyl-glucosamine phosphorylase; β-1,2-mannobiose phosphorylase
	GH132	0	0	0	0	0	1	0	0	n.d.
GT	GT1	0	1	1	0	0	0	0	0	UDP-glucuronosyltransferase; zeatin O-β-xylosyltransferase; 2-hydroxyacylsphingosine 1-β-galactosyltransferase; N-acylsphingosine galactosyltransferase; flavonol 3-O-glucosyltransferase
	GT2	78	72	79	85	76	55	40	30	cellulose synthase; chitin synthase; dolichyl-phosphate β-D-mannosyltransferase; dolichyl-phosphate β-glucosyltransferase; N-acetylglucosaminyltransferase; N-acetylgalactosaminyltransferase
	GT3	0	0	0	0	0	2	0	0	glycogen synthase
	GT4	97	91	142	130	125	98	49	46	sucrose synthase; sucrose-phosphate synthase; α-glucosyltransferas; lipopolysaccharide N-acetylglucosaminyltransferase; phosphatidylinositol α-mannosyltransferase
	GT5	3	4	6	5	9	5	2	2	UDP-Glc: glycogen glucosyltransferase; ADP-Glc: starch glucosyltransferase; NDP-Glc: starch glucosyltransferase; UDP-Glc: α-1,3-glucan synthase; UDP-Glc: α-1,4-glucan synthase
	GT7	0	0	1	0	0	0	0	0	lactose synthase; β-N-acetylglucosaminyl-glycopeptide β-1,4-galactosyltransferase; N-acetyllactosamine synthase; xylosylprotein β-4-galactosyltransferase; UDP-Gal: neolactotriaosylceramide β-1,4-galactosyltransferase
	GT8	4	6	5	3	2	0	3	0	lipopolysaccharide α-1,3-galactosyltransferase; UDP-Glc: (glucosyl)lipopolysaccharide α-1,2-glucosyltransferase;

									lipopolysaccharide glucosyltransferase 1; glycogenin glucosyltransferase
GT9	0	1	1	3	4	0	0	16	lipopolysaccharide N-acetylglucosaminyltransferase; heptosyltransferase
GT12	0	1	0	0	0	4	0	1	[N-acetylneuraminyl]-galactosylglucosylceramide N-acetylgalactosaminyltransferase
GT14	1	0	0	0	0	0	0	0	β-1,3-galactosyl-O-glycosyl-glycoprotein β-1,6-N-acetylglucosaminyltransferase; N-acetyllactosaminide β-1,6-N-acetylglucosaminyltransferase; protein O-β-xylosyltransferase; UDP-GlcA:arabinogalactan β-glucuronosyltransferase
GT15	0	0	0	0	0	2	0	0	glycolipid 2-α-mannosyltransferase; GDP-Man: α-1,2-mannosyltransferase
GT19	6	9	13	12	4	13	3	5	lipid-A-disaccharide synthase
GT20	4	5	7	6	8	2	4	5	α,α-trehalose-phosphate synthase [UDP-forming]; Glucosylglycerol-phosphate synthase; trehalose-6-P phosphatase; [retaining] GDP-valeniol: validamine 7-phosphate valeniolyltransferase
GT21	0	2	3	2	1	1	0	0	UDP-Glc: ceramide β-glucosyltransferase
GT25	0	0	0	0	0	0	0	1	lipopolysaccharide β-1,4-galactosyltransferase; β-1,3-glucosyltransferase; β-1,2-glucosyltransferase; β-1,2-galactosyltransferase; LPS β-1,4-galactosyltransferase; occidiofungin β-xylosyltransferase; UDP-Gal:procollagen β-galactosyltransferase
GT26	16	18	21	18	18	19	15	3	UDP-ManNAcA: β-N-acetyl mannosaminuronyltransferase; UDP-ManNAc: β-N-acetyl-mannosaminyltransferase; UDP-Glc: β-1,4-glucosyltransferase; β-1,4-galactosyltransferase
GT27	3	1	3	3	2	0	1	0	polypeptide α-N-acetylgalactosaminyltransferase
GT28	30	24	26	20	11	35	5	5	1,2-diacylglycerol 3-β-galactosyltransferase; 1,2-diacylglycerol 3-β-glucosyltransferase; UDP-GlcNAc: Und-PP-MurAc-pentapeptide β-N-acetylglucosaminyltransferase; digalactosyldiacylglycerol synthase
GT29	1	0	1	0	1	0	0	0	sialyltransferase; β-galactoside α-2,6-sialyltransferase; α-N-acetylgalactosaminide α-2,6-sialyltransferase; β-galactoside α-2,3-sialyltransferase; N-acetyllactosaminide α-2,3-sialyltransferase
GT30	1	1	1	2	4	4	2	4	CMP-β-KDO: α-3-deoxy-D-manno-octulosonic-acid (KDO) transferase
GT31	0	0	0	0	0	1	0	1	N-acetyllactosaminide β-1,3-N-acetylglucosaminyltransferase; Glycoprotein-N-acetylgalactosamine 3-β-galactosyltransferase;

									fucose-specific β-1,3-N-acetylglucosaminyltransferase; globotriosylceramide β-1,3-GalNAc transferase
GT32	0	0	1	0	1	2	0	0	α-1,6-mannosyltransferase; α-1,4-N-acetylglucosaminyltransferase; α-1,4-N-acetylgalactosaminyltransferase; GDP-Man: inositol-phosphorylceramide transferase; UDP-Gal: β-galactoside α-1,4-galactosyltransferase
GT34	0	0	0	0	0	1	0	0	UDP-Gal: galactomannan α-1,6-galactosyltransferase; UDP-Xyl: xyloglucan α-1,6-xylosyltransferase; α-1,2-galactosyltransferase
GT35	2	3	0	2	2	2	2	2	glycogen or starch phosphorylase
GT39	1	1	4	0	3	4	1	0	Dol-P-Man: protein α-mannosyltransferase
GT44	2	2	0	0	0	1	0	0	UDP-Glc: α-glucosyltransferase; UDP-GlcNAc: α-N-acetylglucosaminyltransferase
GT45	1	0	0	0	0	0	0	0	α-N-acteylglucosaminyltransferase
GT46	0	1	0	0	0	0	0	0	n.d.
GT48	0	0	0	0	0	1	0	0	1,3-β-glucan synthase
GT51	52	47	52	28	32	64	16	19	murein polymerase
GT55	0	0	0	1	1	0	0	1	GDP-Man: mannosyl-3-phosphoglycerate synthase
GT56	0	0	0	0	0	0	0	1	TDP-Fuc4NAc: lipid II Fuc4NAc transferase
GT62	0	0	0	1	0	2	0	0	α-1,2-mannosyltransferase; α-1,6-mannosyltransferase
GT66	0	0	0	0	0	1	1	0	dolichyl-diphosphooligosaccharide—protein glycotransferase; undecaprenyl-diphosphooligosaccharide—protein glycotransferase
GT70	0	0	1	1	0	1	0	0	UDP-GlcA: β-glucuronosyltransferase
GT73	0	0	1	0	0	0	0	1	CMP-β-KDO: α-3-deoxy-D-manno-octulosonic-acid (KDO) transferase
GT76	1	1	1	0	0	0	0	0	Dol-P-Man: α-1,6-mannosyltransferase
GT78	0	1	0	0	1	0	1	0	GDP-Man: α-mannosyltransferase (mannosylglycerate synthase)
GT81	4	5	15	5	8	0	3	1	NDP-Glc: glucosyl-3-phosphoglycerate synthase; NDP-Man: mannosyl-3-phosphoglycerate synthase; ADP-Glc: glucosyl-2-glycerate synthase
GT83	1	1	5	2	0	0	0	1	undecaprenyl phosphate-α-L-Ara4N: 4-amino-4-deoxy-β-L-arabinosyltransferase; dodecaprenyl phosphate-β-galacturonic acid: lipopolysaccharide core α-galacturonosyl transferase
GT84	0	0	1	1	0	0	0	0	cyclic β-1,2-glucan synthase
GT87	1	0	1	0	2	0	1	1	polyprenol-P-Man: α-1,2-mannosyltransferase
GT90	1	0	0	0	0	0	0	0	UDP-Xyl: (mannosyl) glucuronoxylomannan/galactoxylomannan β-1,2-xylosyltransferase; UDP-Glc: protein O-β-

										glucosyltransferase; UDP-Xyl: protein O-β-xylosyltransferase
	GT94	6	4	5	2	1	0	1	1	GDP-Man: GlcA-β-1,2-Man-α-1,3-Glc-β-1,4-Glc-α-1-PP-undecaprenol β-1,4-mannosyltransferase
CE	CE1	42	46	61	46	45	53	14	23	acetyl xylan esterase; cinnamoyl esterase; feruloyl esterase; carboxylesterase; S-formylglutathione hydrolase; diacylglycerol O-acyltransferase ; trehalose 6-O-mycolyltransferase
	CE2	2	1	0	0	2	2	0	0	acetyl xylan esterase
	CE3	10	14	9	7	13	9	6	4	acetyl xylan esterase
	CE4	61	73	83	55	41	61	28	18	acetyl xylan esterase; chitin deacetylase; chitooligosaccharide deacetylase; peptidoglycan GlcNAc deacetylase; peptidoglycan N-acetylmuramic acid deacetylase
	CE5	0	0	2	0	1	0	0	0	acetyl xylan esterase; cutinase
	CE6	1	0	3	4	1	2	0	0	acetyl xylan esterase
	CE7	6	4	5	6	8	4	7	3	acetyl xylan esterase; cephalosporin-C deacetylase
	CE8	1	1	2	3	1	1	0	1	pectin methylesterase
	CE9	8	10	23	11	5	17	2	1	N-acetylglucosamine 6-phosphate deacetylase; N-acetylglucosamine 6-phosphate deacetylase
	CE10	22	23	26	23	22	34	11	14	arylesterase; carboxyl esterase; acetylcholinesterase; cholinesterase; sterol esterase; brefeldin A esterase
	CE11	18	17	12	10	22	20	10	4	UDP-3-0-acyl N-acetylglucosamine deacetylase
	CE12	11	4	2	3	5	4	3	0	pectin acetylesterase; rhamnogalacturonan acetylesterase; acetyl xylan esterase
	CE14	22	20	35	21	15	25	10	11	N-acetyl-1-D-myo-inosityl-2-amino-2-deoxy-α-D-glucopyranoside deacetylase; diacetylchitobiose deacetylase; mycothiol S-conjugate amidase
	CE15	2	1	5	5	7	1	3	0	4-O-methyl-glucuronoyl methylesterase
	CE16	1	0	0	0	1	1	0	0	acetylesterase
PL	PL1	13	4	2	3	5	0	4	1	pectate lyase; exo-pectate lyase; pectin lyase
	PL3	8	4	1	2	2	0	2	0	pectate lyase
	PL4	0	0	0	0	0	0	0	0	rhamnogalacturonan lyase
	PL6	1	1	0	0	0	0	0	1	alginate lyase; chondroitinase B; MG-specific alginate lyase
	PL9	4	0	2	5	4	0	2	0	pectate lyase; exopolygalacturonate lyase; thiopeptidoglycan lyase
	PL10	2	0	0	2	3	0	2	0	pectate lyase
	PL11	4	0	2	2	1	0	1	0	rhamnogalacturonan lyase; exo-unsaturated rhamnogalacturonan lyase

	PL12	1	0	1	0	2	0	2	1	heparin-sulfate lyase
	PL14	0	1	1	1	3	0	1	0	alginate lyase; exo-oligoalginate lyase; β-1,4-glucuronan lyase
	PL15	0	0	1	1	4	0	3	1	oligo-alginate lyase; alginate lyase
	PL17	1	0	1	2	3	0	2	0	alginate lyase; oligoalginate lyase
	PL18	0	1	0	0	0	0	0	1	alginate lyase; poly(α-L-guluronate) lyase / G-specific alginate lyase; MG-specific alginate lyase
	PL20	0	1	0	0	0	0	0	0	endo-β-1,4-glucuronan lyase
	PL21	0	0	0	1	0	0	0	0	heparin lyase; heparin-sulfate lyase; acharan-sulfate lyase
	PL22	4	4	3	5	4	0	1	1	oligogalacturonate lyase / oligogalacturonide lyase
AA	AA2	13	10	3	6	6	18	2	14	manganese peroxidase; versatile peroxidase; lignin peroxidase; peroxidase
	AA3	1	0	1	1	1	3	0	0	cellobiose dehydrogenase; glucose 1-oxidase; aryl alcohol oxidase; alcohol oxidase; pyranose oxidase
	AA4	7	5	10	7	2	3	0	1	vanillyl-alcohol oxidase
	AA6	19	23	18	15	10	57	7	23	1,4-benzoquinone reductase
	AA7	0	0	7	1	1	0	4	6	glucooligosaccharide oxidase; chitooligosaccharide oxidase
	AA9	0	0	2	0	0	2	0	0	copper-dependent lytic polysaccharide monooxygenases
	AA10	7	6	5	6	4	4	9	7	copper-dependent lytic polysaccharide monooxygenases

Table S19. Number of CDS from reconstructed genome (OTU537822506) mapped to CAZy database

Type	Family	CDS	Known Activities
SLH		8	cellulosome domain
CBM	13	1	xylan-binding domain
	37	1	xylan, chitin and cellulose-binding
	50	9	peptidoglycan-binding domain
	66	1	fructans-binding domain
GH	11	1	endo-β-1,4-xylanase; endo-β-1,3-xylanase
	16	1	xyloglucan:xyloglucosyltransferase; endo-1,4-β-galactosidase; endo-1,3-β-glucanase
	18	1	chitinase; lysozyme; endo-β-N-acetylglucosaminidase
	23	4	lysozyme type G; peptidoglycan lyase; chitinase
	74	1	endoglucanase; oligoxyloglucan reducing end-specific cellobiohydrolase; xyloglucanase
	109	1	α-N-acetylgalactosaminidase
	130	2	β-1,4-mannosylglucose phosphorylase; β-1,4-mannooligosaccharide phosphorylase
GT	2	3	cellulose synthase; chitin synthase; N-acetylglucosaminyltransferase
	4	4	sucrose synthase; sucrose-phosphate synthase; α-glucosyltransferase
	19	1	lipid-A-disaccharide synthase
	27	1	polypeptide α-N-acetylgalactosaminyltransferase
	28	4	1,2-diacylglycerol 3-β-galactosyltransferase; 1,2-diacylglycerol 3-β-glucosyltransferase
	51	3	murein polymerase
	94	1	GDP-Man: GlcA-β-1,2-Man-α-1,3-Glc-β-1,4-Glc-α-1-PP-undecaprenol β-1,4-mannosyltransferase
PL	1	1	pectate lyase
CE	1	5	acetyl xylan esterase; feruloyl esterase; carboxylesterase
	4	5	chitin deacetylase
AA	4	3	vanillyl-alcohol oxidase
	6	1	1,4-benzoquinone reductase
