# Supplementary material for: Microbial community structure and dynamics in thermophilic composting viewed through metagenomics and metatranscriptomics
Source: Sci Rep. 2016 Dec 12;6:38915. doi: 10.1038/srep38915 (PMC5150989; doi:10.1038/srep38915)
Supplement: Supplementary Figures [file srep38915-s2.pdf]

Supplementary Figures

Title: Microbial community structure and dynamics in thermophilic composting viewed through metagenomics and metatranscriptomics

Authors:
Luciana Principal Antunes[1]
Layla Farage Martins[1]
Roberta Verciano Pereira[1]
Andrew Maltez Thomas[1,2]
Deibs Barbosa[1,2]
Leandro Lemos Nascimento[1,2]
Gianluca Major Machado Silva[1,2]
Livia Maria Silva Moura[1,2]
George Willian Condomitti Epamino[1,2]
Luciano Antonio Digiampietri[3]
Karen Cristina Lombardi[1]
Patricia Locosque Ramos[4]
Ronaldo Bento Quaggio[1]
Julio Cezar Franco de Oliveira[5]
Renata Castiglioni Pascon[5]
João Batista da Cruz[4]
Aline Maria da Silva[1,2,*]
João Carlos Setubal[1,2,6,*]

Affiliations:
[1] Departamento de Bioquímica, Instituto de Química, Universidade de São Paulo, São Paulo, Brazil
[2] Programa de Pós-Graduação Interunidades em Bioinformática, Universidade de São Paulo, São Paulo, Brazil
[3] Escola de Artes, Ciências e Humanidades, Universidade de São Paulo, São Paulo, Brazil
[4] Fundação Parque Zoológico de São Paulo, São Paulo, Brazil
[5] Departamento de Ciências Biológicas, Universidade Federal de São Paulo, São Paulo, Brazil
[6] Biocomplexity Institute of Virginia Tech, Blacksburg, VA, USA
[*] These authors shared senior authorship

Correspondence and requests for materials should be addressed to: J.C.S. (setubal@iq.usp.br) or A.M.D.S. (almsilva@iq.usp.br)

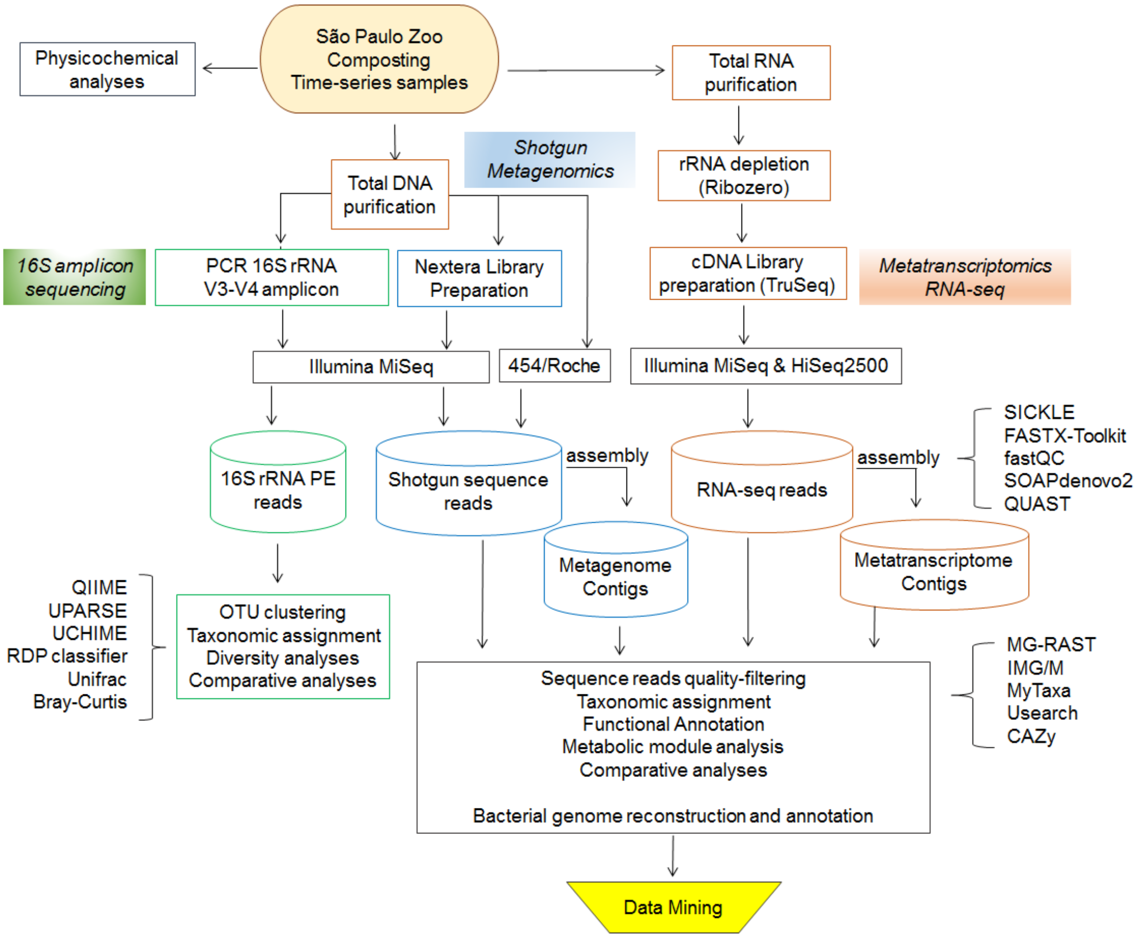

Figure S1. Data mining workflow. Schematic representation of the strategy used for taxonomic and functional analyses combining metagenomic (16S rRNA amplicon and shotgun approaches) and metatranscriptomic datasets. See Methods for detailed information.

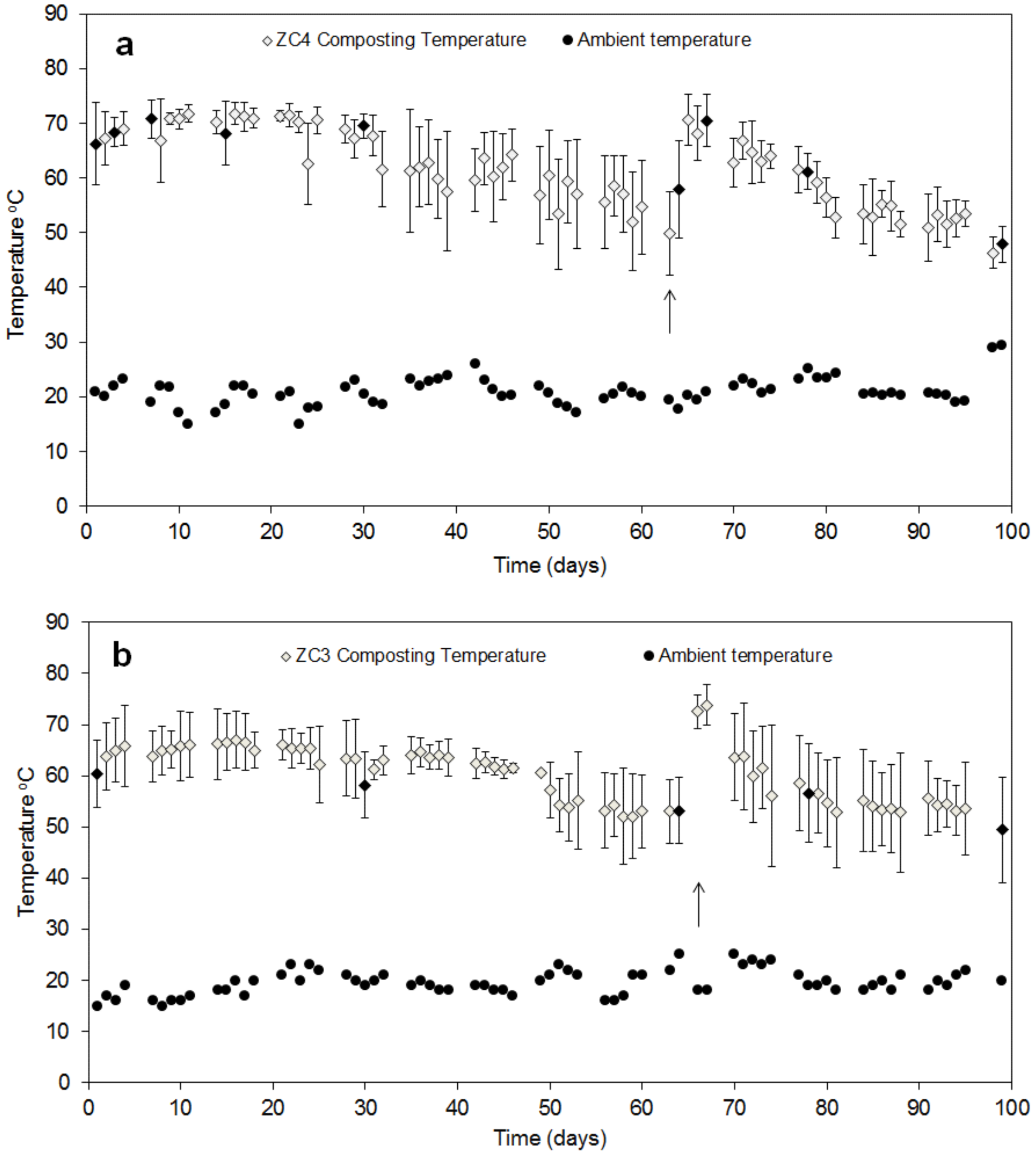

Figure S2. Temperature profile during composting process of ZC4 and ZC3 cells. Temperature of ZC4 (a) and ZC3 (b) composting cells and of ambient were measured daily. Error bars represent standard deviation of measurements taken at the four edges and in the center of the pile. Black diamonds indicate days of sample collections. Arrow indicates the turning procedure in ZC4 (day 63) and ZC3 (day 65).

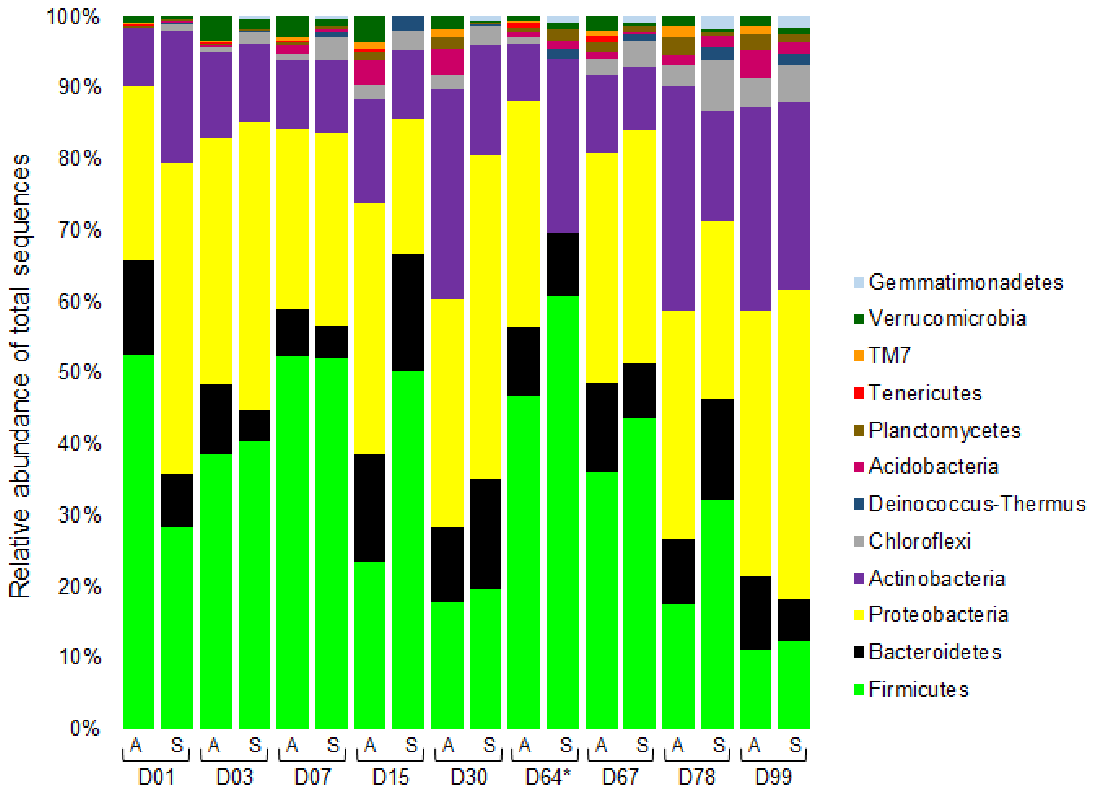

Figure S3. Taxonomic assignment of bacterial phyla during ZC4 composting process. Relative abundance of bacteria at phylum level was assigned from 16S rRNA amplicon (A) and shotgun (S) sequencing datasets from ZC4 samples using RDP classifier and MG-RAST (M5NR) database, respectively. Only phyla with relative abundance >1% are shown. Unassigned reads for 16S (7-30%) and for shotgun (10.2-15.2%) were excluded. Samples are referred to by the letter D followed by the collection day. Asterisk indicates one day after the turning procedure.

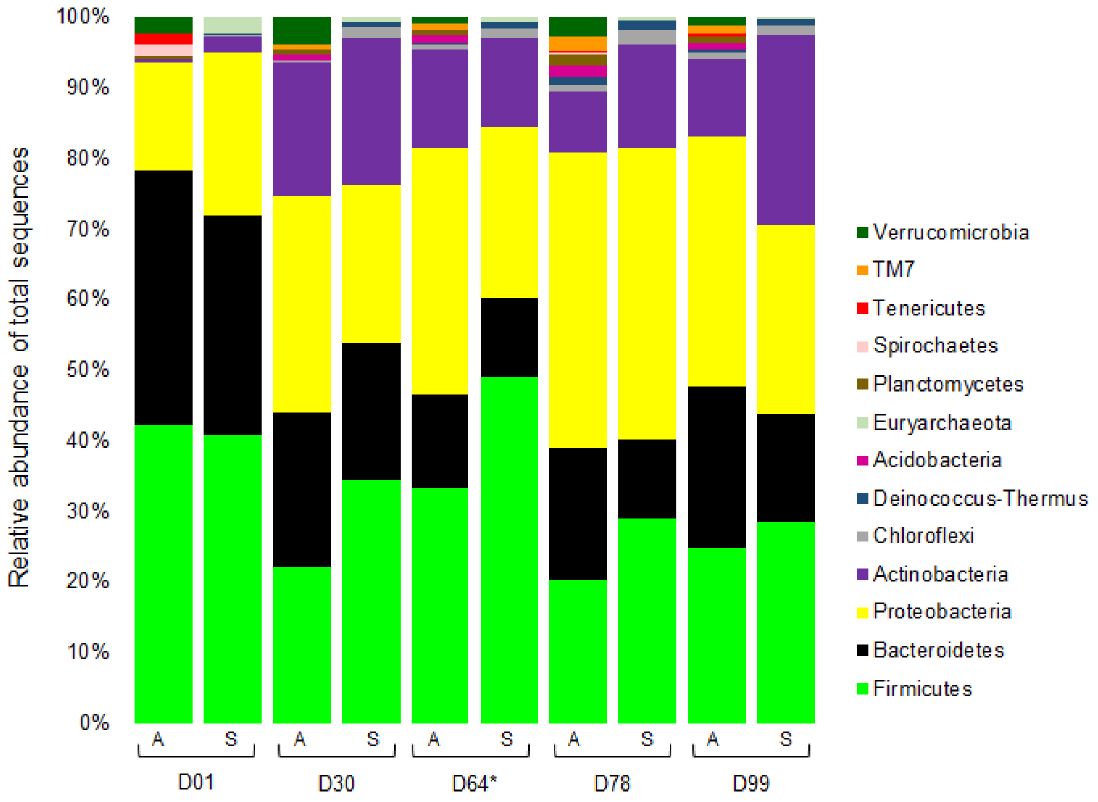

Figure S4. Taxonomic assignment of bacterial phyla during ZC3 composting process. Relative abundance of bacteria at phylum level was assigned from 16S rRNA amplicon (A) and shotgun (S) sequencing datasets from ZC3 samples using RDP classifier and MG-RAST (M5NR) database, respectively. Only phyla with relative abundance >1% are shown. Unassigned reads for 16S (16-29%) and for shotgun (8.5-13.2%) were excluded. Samples are referred to by the letter D followed by the collection day. Asterisk indicates one day before the turning procedure.

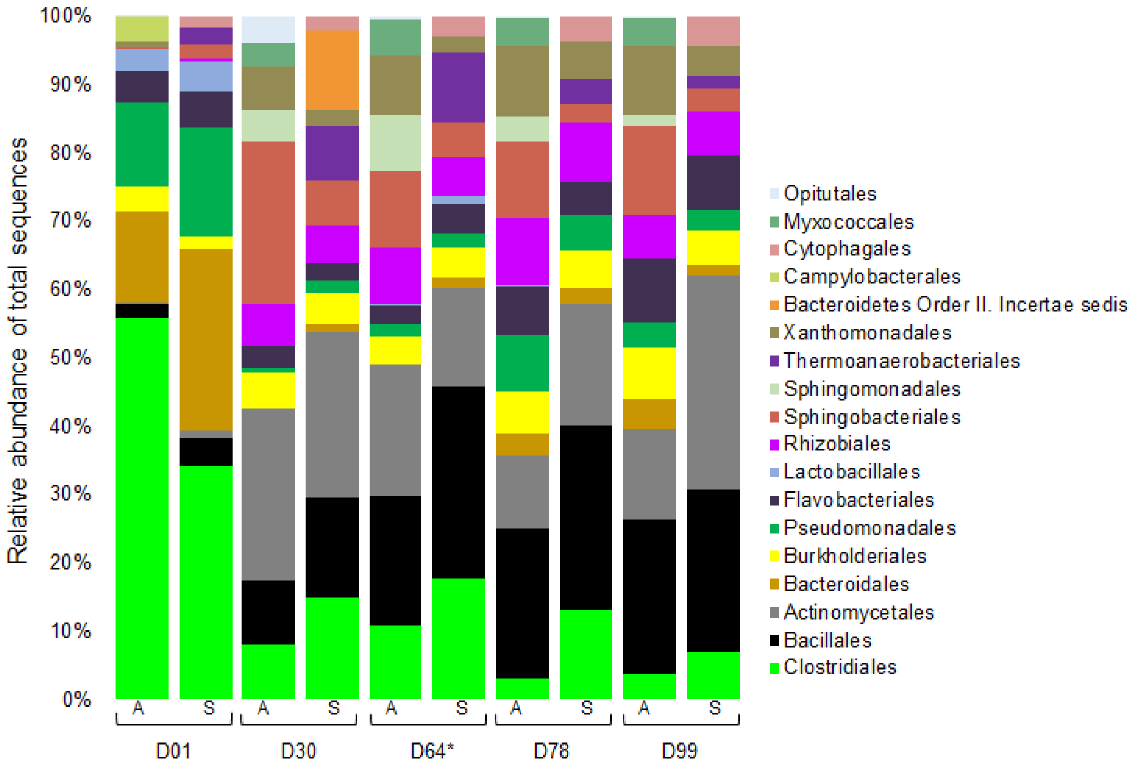

Figure S5. Taxonomic assignment of bacterial order during ZC3 composting process. Relative abundance of bacteria at order level was assigned from 16S rRNA amplicon (A) and shotgun (S) sequencing datasets from ZC3 samples using RDP classifier and MG-RAST (M5NR) database, respectively. Only orders with relative abundance >1% are shown. Unassigned reads for 16S (16-29%) and for shotgun (8.2-12.9%) were excluded. Samples are referred to by the letter D followed by the collection day. Asterisk indicates one day before the turning procedure.

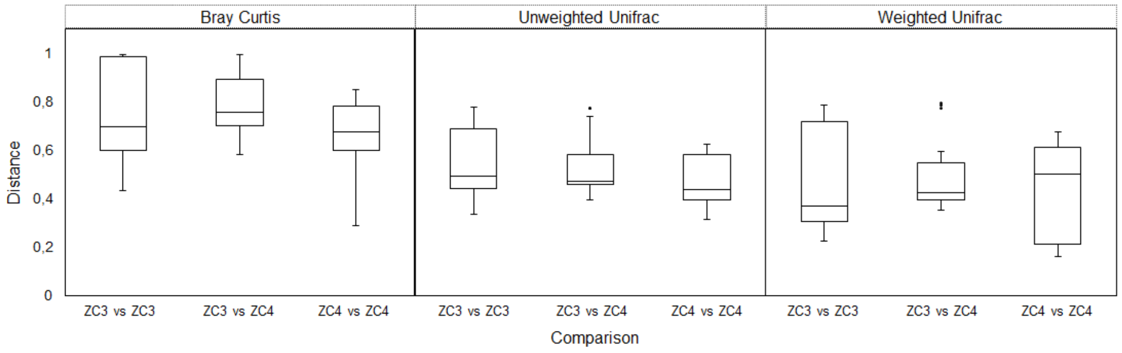

Figure S6. Phylogenetic comparison between ZC3 and ZC4 bacterial communities. The OTUs assigned by16S rRNA amplicon dataset from each equivalent time sample of ZC3 and ZC4 (D01, D30, D78 and D99) were compared using Bray Curtis and Unifraq (Unweighted and Weighted) metrics. Box plots represent sample comparisons within the same cell (ZC3 versus ZC3 or ZC4 versus ZC4) or from distinct cells (ZC3 versus ZC4). The points indicate outliers.

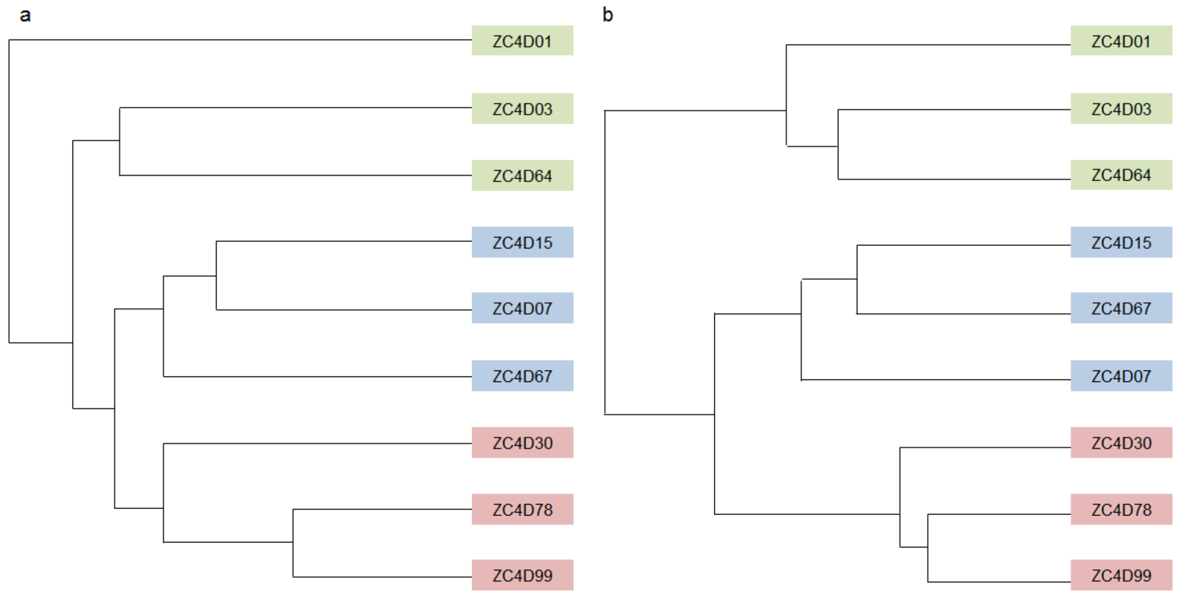

Figure S7. Hierarchical clustering of ZC4 composting samples based on 16S amplicon data. Bacterial community comparison for each time point using group-average clustering of 16S amplicon data based on Bray-Curtis similarity (A) and weighted Unifraq (B) matrices. Samples are referred to by the letter D followed by the collection day.

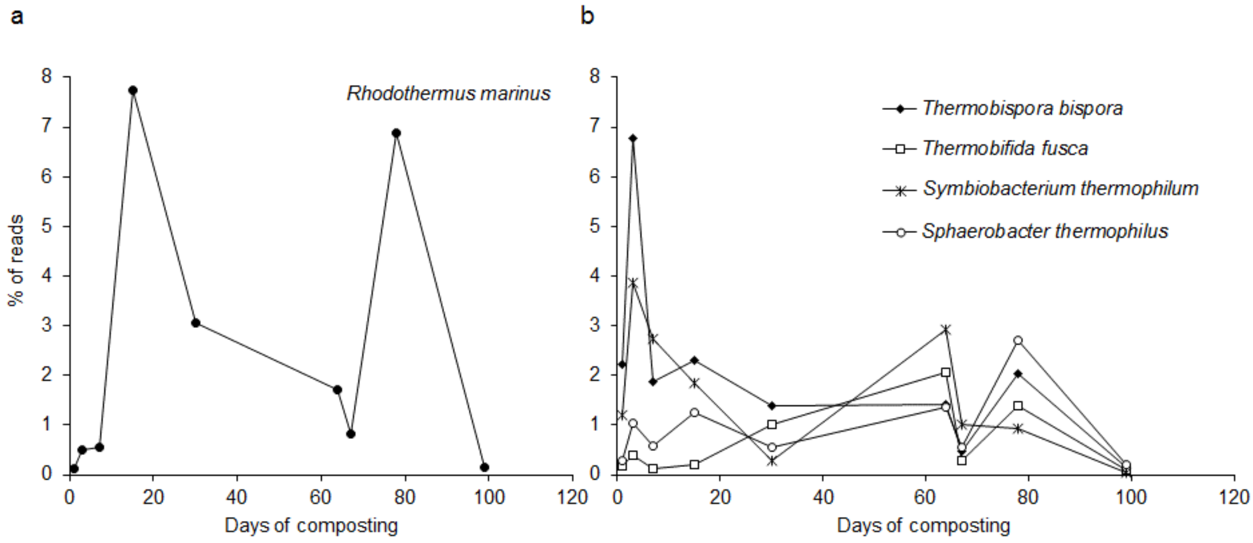

Figure S8. Variation in relative abundance of the top five bacterial species over days of ZC4 composting. Relative abundance was calculated using total reads classified by MyTaxa in ZC4 metagenomic data. The highest relative abundant organism is *R. marinus* (a) followed by *T. bispora*, *S. thermophilum*, *S. thermophilus* and *T. fusca* (b).

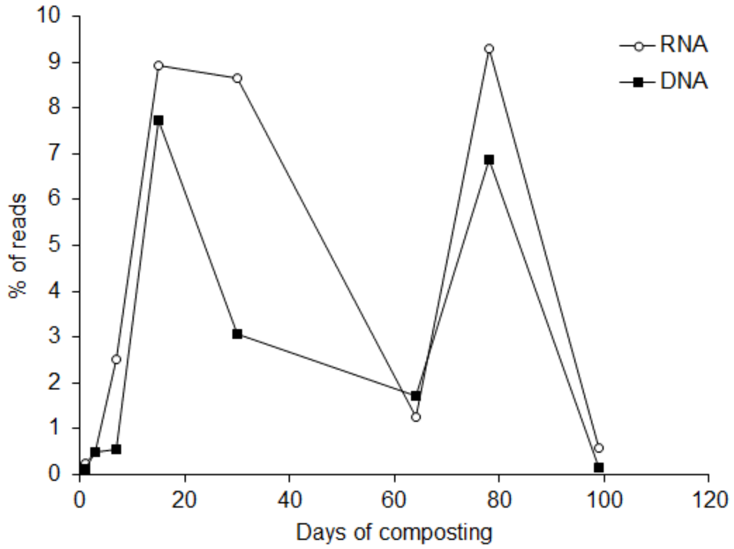

Figure S9. Variation in relative abundance of reads classified as *Rhodothermus marinus* by MyTaxa in ZC4 metagenomes (DNA) and metatranscriptomes (RNA). Percentage of classified reads was normalized across ZC4 samples.

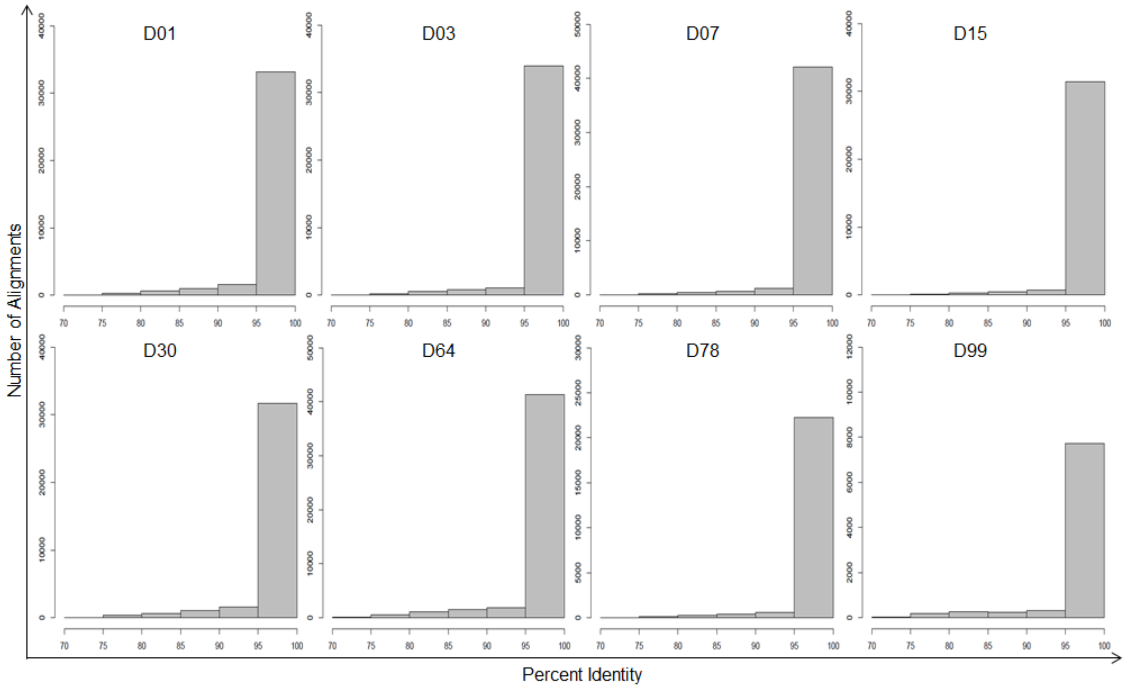

Figure S10. Number of RNA reads that were mapped to DNA contigs in each sample. Each graph is a histogram, and each bin shows the number of reads that were mapped at a given threshold of identity. The vast majority of RNA reads could be mapped at the 95% level or higher. Samples are referred to by the letter D followed by the collection day.

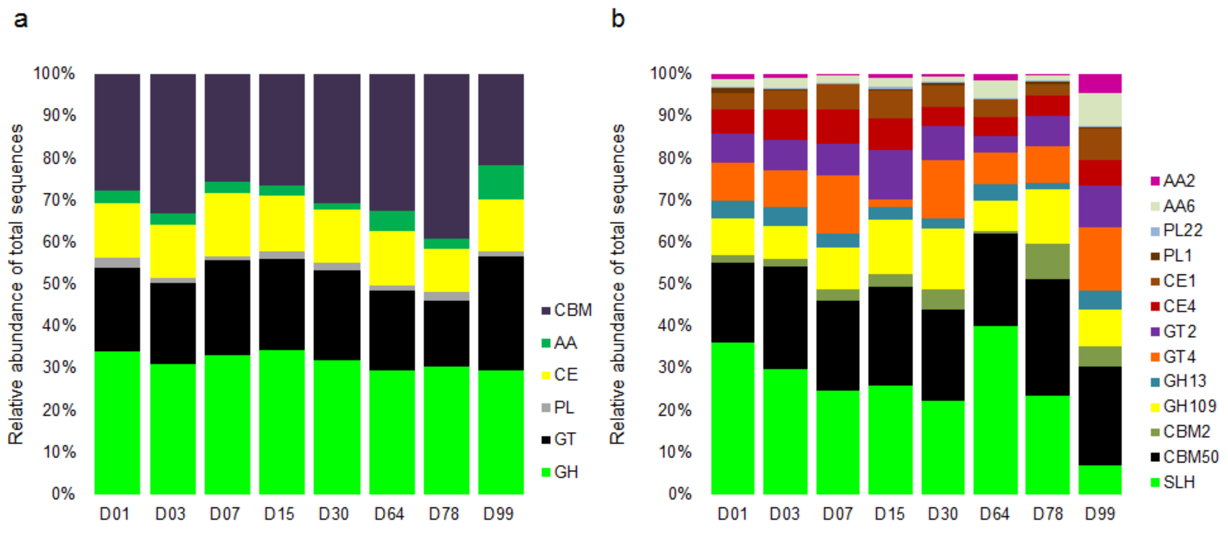

Figure S11. Carbohydrate-active functional profile in ZC4 metatranscriptome. (a) Relative abundance (%) of each CAZy class in each metatranscriptome. (b) Relative abundance (%) of the most abundant families classified per CAZy class/module. Samples are referred to by the letter D followed by the collection day.

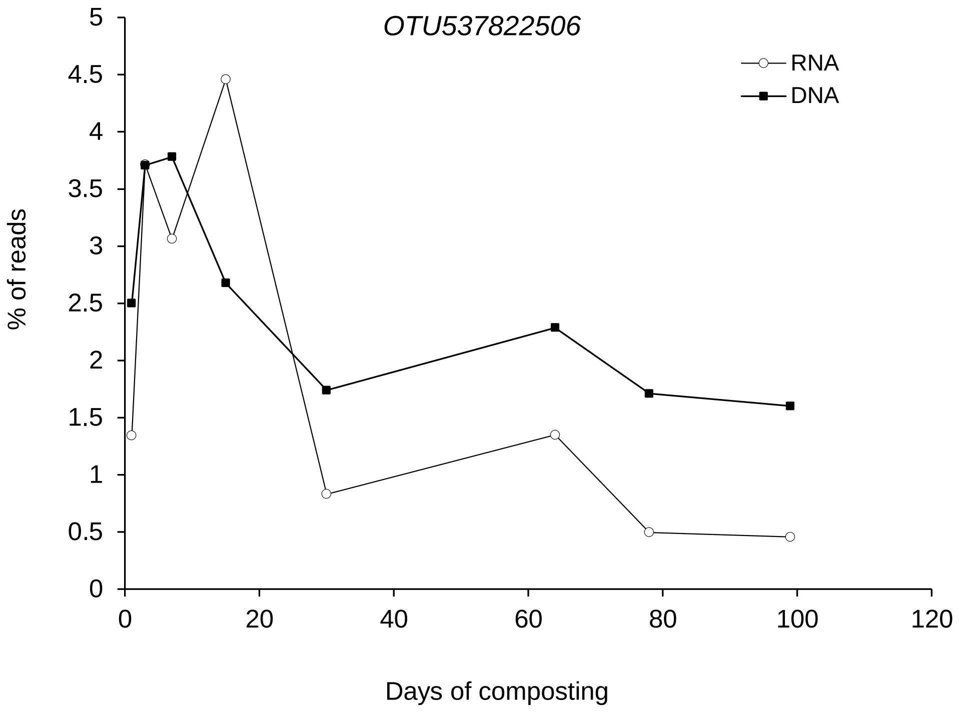

Figure S12. Variation of metagenome and metatranscriptome reads from OTU537822506 over days of ZC4 composting. Relative abundance of reads was calculated using total reads of OTU537822506 per total reads in the ZC4 metagenomic (DNA) or metatranscriptomic (RNA) data.
