# Supplementary material for: Microbial community structure and dynamics in thermophilic composting viewed through metagenomics and metatranscriptomics
Source: Sci Rep. 2016 Dec 12;6:38915. doi: 10.1038/srep38915 (PMC5150989; doi:10.1038/srep38915)
Supplement: Supplementary Methods [file srep38915-s3.pdf]

Supplementary Methods

Title: Microbial community structure and dynamics in thermophilic composting viewed through metagenomics and metatranscriptomics

Authors:
Luciana Principal Antunes[1]
Layla Farage Martins[1]
Roberta Verciano Pereira[1]
Andrew Maltez Thomas[1,2]
Deibs Barbosa[1,2]
Leandro Lemos Nascimento[1,2]
Gianluca Major Machado Silva[1,2]
Livia Maria Silva Moura[1,2]
George Willian Condomitti Epamino[1,2]
Luciano Antonio Digiampietri[3]
Karen Cristina Lombardi[1]
Patricia Locosque Ramos[4]
Ronaldo Bento Quaggio[1]
Julio Cezar Franco de Oliveira[5]
Renata Castiglioni Pascon[5]
João Batista da Cruz[4]
Aline Maria da Silva[1,2,*]
João Carlos Setubal[1,2,6,*]

Affiliations:
[1] Departamento de Bioquímica, Instituto de Química, Universidade de São Paulo, São Paulo, Brazil
[2] Programa de Pós-Graduação Interunidades em Bioinformática, Universidade de São Paulo, São Paulo, Brazil
[3] Escola de Artes, Ciências e Humanidades, Universidade de São Paulo, São Paulo, Brazil
[4] Fundação Parque Zoológico de São Paulo, São Paulo, Brazil
[5] Departamento de Ciências Biológicas, Universidade Federal de São Paulo, São Paulo, Brazil
[6] Biocomplexity Institute of Virginia Tech, Blacksburg, VA, USA
[*] These authors shared senior authorship

Correspondence and requests for materials should be addressed to: J.C.S. (setubal@iq.usp.br) or A.M.D.S. (almsilva@iq.usp.br)

Annotation and analyses of metagenomic and metatranscriptomic sequences dataset

The IMG/M[1] pipeline assigns functions to coding sequences based on COG categories [2], Pfam families [3] and EC numbers. Samples were compared in terms of these annotations using the IMG/M function comparison tool (binomial test). For all tests of statistical overrepresentation, we used a maximum *p*-value of 0.05. Predicted protein coding sequences annotated in the contigs dataset were also screened for carbohydrate-active enzymes using BLASTP[4] against the CAZy database[5].

Concordance between ZC4 metagenomics and metatranscriptomics datasets for each sample was verified by aligning their respective contigs using BLASTN[4].

Microbial community analyses

Microbial composition analyses based on unassembled shotgun metagenomics reads uploaded on MG-RAST[6,7] were performed using best hit classification tool against the M5NR (M5 non-redundant protein) database available within MG-RAST (version 3.6), using minimal identity of 60%, maximum e-value cutoff of $1x10^{-5}$ and minimal alignment length of 50 bp. Both read1 and read2 were taken into account to estimate relative abundance of taxonomic levels.

For microbial community analyses based on 16S rRNA amplicon sequencing, PE sequence reads from the16S metagenomic sequencing libraries were joined using fastq-join[8]. Using QIIME tool kit[9], we trimmed both forward and reverse 16S rRNA primers allowing a maximum of 2 mismatches, removed sequences with a mean quality threshold below phred 20 using a 50 bp sliding window and removed sequences smaller than 200 bp. Quality filtered sequences were then clustered into OTUs using UPARSE[10] at the 97% identity threshold and chimera-filtered using UCHIME[10]. Representative seed sequences were used for taxonomic identification using the RDP classifier[11] and a confidence threshold of 80%. For Unifrac

diversity measurements, a phylogenetic tree was constructed by aligning representative sequences to the Greengenes core set[12] using PyNAST[13] and FastTree2[14].

QIIME version 1.8 was used for alpha and beta diversity analyses. Samples were rarefied to a depth of 517,210 sequences and using the average of 10 iterations. Alpha diversity was calculated using phylogenetic diversity[15] and species richness, beta diversity was calculated using Unifrac[16] and Bray-Curtis.

Taxonomic classification of shotgun DNA reads and coding sequences

We performed taxonomic classification of shotgun DNA reads in the metagenome and coding sequences in the metatranscriptome assembled contigs using program MyTaxa[17].

Genome reconstruction based on reference genomes

Sequences of the five most abundant bacterial species as classified by MyTaxa[17] in ZC4 metagenomes were recruited and aligned to the reference genomes for these species, using FR-HIT[18]. Sequences that aligned to each one of the reference genomes with 85% identity and e-value < 0.00005 were filtered and assembled using Newbler[19]. Reconstructed genomes were annotated with PROKKA[20], Prodigal[21] and RNAmmer[22].

Method used to reconstruct the genome of OTU537822506

Shotgun DNA reads classified by MyTaxa as ‘unassigned Firmicutes’ (a total of 1,581,916 reads) were used as input for the following pipeline. The reads were assembled with SPADES (option --meta)[23], generating 50,909 contigs. These contigs were used as input for program MaxBin[24] (with parameter -min_contig_length 1000), which placed these contigs into 13 bins. By comparing the full-length 16S sequence of C*alditerricola yamamurae* (GenBank accession number AB308475.1) and the draft genome of *Calditerricola satsumensis* JCM 14719 (NCBI Reference Sequence NZBBCF0000000.1) against the sequences in these bins, we identified bin 5

as the one containing genome sequences of OTU537822506. This partial genome contains 452 contigs, with a total of 685,090 bp. We then extended this genome with the following strategy. To the “unassigned firmicutes” reads we added all unclassified reads. We assembled this dataset using SPADES (option --meta) resulting in 1,162,607 contigs. We then used MaxBin as done previously, obtaining this time 91 bins. We used bin 5 form the previous round as query on a BLASTn against the 91 bins from the second round. Bin 3 from the second round was found to correspond to the genome. Its contigs were used as input for CheckM[25] so we could identify multiple single copy markers and remove them from the final draft genome. This draft genome has 635 contigs, with a total of 2,367,546 bp.

Method for inferring phylogenetic tree with proposed novel Bacillaceae genus

Based on the fact that *Calditerricola yamamurae* is a member of the family Bacillaceae, we chose six closely related organisms for which there are complete annotated genomes available: *Bacillus subtilis* subsp. *subtilis* str. 168, *Calditerricola satsumensis* JCM 14719, *Geobacillus* sp lc300, *Geobacillus thermoglucosidasius* strain dsm 2542 [26,27], *Lactobacillus hokkaidonensis* and *Thermaerobacter marianensis* dsm 12885. The latter was chosen as outgroup, since it is a member of the order Bacillales but not a member of the family Bacillaceae. We then ran the program get_homologues[28] (parameters -M and -S 50) on these genomes to obtain ortholog gene families. Next, we selected a set of 113 families to build the tree; these are single-copy genes present in all genomes. The nucleotide sequences in each family were aligned with MUSCLE[29], and the resulting alignments were input to the alignment-correction program Guidance[30]. The output was concatenated using FasConCat[31], generating an alignment with 72,396 columns. Then the following steps were run: partitionFinder[32] (seeks best partition to generate a tree), IQ-

TREE[33] (tree inference by maximum likelihood), and figTree (http://tree.bio.ed.ac.uk/software/figtree/) (tree visualization).

Mapping of metagenomic and metatranscriptome reads to OTU537822506 genome

Metatranscriptome reads were quality-checked (Q20, min Length 50) with Sickle (https://github.com/najoshi/sickle). OTU537822506 shotgun reads were mapped to these reads using BWA[34].

References

1. Markowitz, V. M. *et al.* IMG/M: the integrated metagenome data management and comparative analysis system. *Nucleic Acids Res* **40**, D123-129 (2012).

2. Galperin, M. Y., Makarova, K. S., Wolf, Y. I. & Koonin, E. V. Expanded microbial genome coverage and improved protein family annotation in the COG database. *Nucleic Acids Res* **43**, D261-D269 (2015).

3. Finn, R. D. *et al.* Pfam: the protein families database. *Nucleic Acids Res* **42**, D222-D230 (2014).

4. Altschul, S. F. *et al.* Gapped BLAST and PSI-BLAST: a new generation of protein database search programs. *Nucleic Acids Res* **25**, 3389-3402 (1997).

5. Cantarel, B. L. *et al.* The Carbohydrate-Active EnZymes database (CAZy): an expert resource for Glycogenomics. *Nucleic Acids Res* **37**, D233-238 (2009).

6. Meyer, F. *et al.* The metagenomics RAST server - a public resource for the automatic phylogenetic and functional analysis of metagenomes. *BMC Bioinformatics* **9**, 386 (2008).

7. Glass, E. M., Wilkening, J., Wilke, A., Antonopoulos, D. & Meyer, F. Using the metagenomics RAST server (MG-RAST) for analyzing shotgun metagenomes. *Cold Spring Harb Protoc* **2010**, pdb prot5368 (2010).

8. Aronesty, E. Comparison of Sequencing Utility Programs. *The Open Bioinformatics Journal* **7**, 1-8 (2013).

9. Caporaso, J. G. *et al.* QIIME allows analysis of high-throughput community sequencing data. *Nat Methods* **7**, 335-336 (2010).

10. Edgar, R. C. UPARSE: highly accurate OTU sequences from microbial amplicon reads. *Nat Methods* **10**, 996-998 (2013).

11. Wang, Q., Garrity, G. M., Tiedje, J. M. & Cole, J. R. Naive Bayesian classifier for rapid assignment of rRNA sequences into the new bacterial taxonomy. *Appl Environ Microbiol* **73**, 5261-5267 (2007).

12. McDonald, D. *et al.* An improved Greengenes taxonomy with explicit ranks for ecological and evolutionary analyses of bacteria and archaea. *Isme J* **6**, 610-618 (2012).

13. Caporaso, J. G. *et al.* PyNAST: a flexible tool for aligning sequences to a template alignment. *Bioinformatics* **26**, 266-267 (2010).

14. Price, M. N., Dehal, P. S. & Arkin, A. P. FastTree 2--approximately maximum-likelihood trees for large alignments. *PLoS One* **5**, e9490 (2010).

15. Faith, D. P., Lozupone, C. A., Nipperess, D. & Knight, R. The cladistic basis for the phylogenetic diversity (PD) measure links evolutionary features to environmental gradients and supports broad applications of microbial ecology's "phylogenetic beta diversity" framework. *Int J Mol Sci* **10**, 4723-4741 (2009).

16. Lozupone, C. & Knight, R. UniFrac: a new phylogenetic method for comparing microbial communities. *Appl Environ Microbiol* **71**, 8228-8235 (2005).

17. Luo, C., Rodriguez, R. L. & Konstantinidis, K. T. MyTaxa: an advanced taxonomic classifier for genomic and metagenomic sequences. *Nucleic Acids Res* **42**, e73 (2014).

18. Niu, B., Zhu, Z., Fu, L., Wu, S. & Li, W. FR-HIT, a very fast program to recruit metagenomic reads to homologous reference genomes. *Bioinformatics* **27**, 1704-1705 (2011).

19. Margulies, M. *et al.* Genome sequencing in microfabricated high-density picolitre reactors. *Nature* **437**, 376-380 (2005).

20. Seemann, T. Prokka: rapid prokaryotic genome annotation. *Bioinformatics* **30**, 2068-2069 (2014).

21. Hyatt, D. *et al.* Prodigal: prokaryotic gene recognition and translation initiation site identification. *BMC Bioinformatics* **11** (2010).

22. Lagesen, K. *et al.* RNAmmer: consistent and rapid annotation of ribosomal RNA genes. *Nucleic Acids Res* **35**, 3100-3108 (2007).

23. Bankevich, A. *et al.* SPAdes: A New Genome Assembly Algorithm and Its Applications to Single-Cell Sequencing. *Journal of Computational Biology* **19**, 455-477 (2012).

24. Wu, Y. W., Tang, Y. H., Tringe, S. G., Simmons, B. A. & Singer, S. W. MaxBin: an automated binning method to recover individual genomes from metagenomes using an expectation-maximization algorithm. *Microbiome* **2** (2014).

25. Parks, D. H., Imelfort, M., Skennerton, C. T., Hugenholtz, P. & Tyson, G. W. CheckM: assessing the quality of microbial genomes recovered from isolates, single cells, and metagenomes. *Genome Res* **25**, 1043-1055 (2015).

26. Ishii, K., Fukui, M. & Takii, S. Microbial succession during a composting process as evaluated by denaturing gradient gel electrophoresis analysis. *Journal of Applied Microbiology* **89**, 768-777 (2000).

27. Ishii, K. & Takii, S. Comparison of microbial communities in four different composting processes as evaluated by denaturing gradient gel electrophoresis analysis. *Journal of Applied Microbiology* **95**, 109-119 (2003).

28. Contreras-Moreira, B. & Vinuesa, P. GET_HOMOLOGUES, a Versatile Software Package for Scalable and Robust Microbial Pangenome Analysis. *Appl Environ Microbiol* **79**, 7696-7701 (2013).

29. Edgar, R. C. MUSCLE: multiple sequence alignment with high accuracy and high throughput. *Nucleic Acids Res* **32**, 1792-1797 (2004).

30. Penn, O. *et al.* GUIDANCE: a web server for assessing alignment confidence scores. *Nucleic Acids Res* **38**, W23-W28 (2010).

31. Kuck, P. & Meusemann, K. FASconCAT: Convenient handling of data matrices. *Mol Phylogenet Evol* **56**, 1115-1118 (2010).

32. Lanfear, R., Calcott, B., Ho, S. Y. W. & Guindon, S. PartitionFinder: Combined Selection of Partitioning Schemes and Substitution Models for Phylogenetic Analyses. *Mol Biol Evol* **29**, 1695-1701 (2012).

33. Nguyen, L. T., Schmidt, H. A., von Haeseler, A. & Minh, B. Q. IQ-TREE: a fast and effective stochastic algorithm for estimating maximum-likelihood phylogenies. *Mol Biol Evol* **32**, 268-274 (2015).

34. Li, H. & Durbin, R. Fast and accurate short read alignment with Burrows-Wheeler transform. *Bioinformatics* **25**, 1754-1760 (2009).
